# Combining in vitro and in silico approaches to model neural tube patterning and isthmic organizer formation

**DOI:** 10.1038/s41540-026-00813-0

**Published:** 2026-08-21

**Authors:** Fredrik Bertilsson, Alexander Degener, Paulina Ibek, Gaurav Singh Rathore, Emil Andersson, Pedro Rifes, Agnete Kirkeby, Victor Olariu

**Affiliations:** 1 Computational Science for Health and Environment, Department of Earth and Environmental Sciences, Lund University, Lund, Sweden; 2 Department of Experimental Medical Science, Lund University, Lund, Sweden; 3 Department of Neuroscience, University of Copenhagen, Copenhagen, Denmark; 4 Developmental and Regenerative Neurobiology, Department of Experimental Medical Science, Lund University, Lund, Sweden; 5 Wallenberg Neuroscience Center and Lund Stem Cell Center, Lund University, Lund, Sweden

**Keywords:** Computational biology and bioinformatics, Developmental biology, Neuroscience, Systems biology

## Abstract

During early embryonic development, the human neural tube is formed and patterned through spatial regionalization of cell identity, driven by gene regulatory responses to morphogen gradients. However, many of the underlying mechanisms remain unclear. Here, we integrate single-cell RNA sequencing data from in vitro emulation of neural tube patterning to develop computational models of rostral-caudal and dorsal-ventral patterning. By embedding these models in a 3D geometry, we reveal how transient morphogen signals induce irreversible patterns consistent with developmental biology and experimental data. Notably, our framework accurately captures the formation and maintenance of the isthmic organizer at the mid-hindbrain boundary, providing a realistic and mechanistic picture of neural tube patterning. This integrated approach bridges in vitro experimentation and computational modeling to uncover fundamental principles of neural development.

## Introduction

The human central nervous system (CNS) consists of a highly diverse range of cell types. Comprising the brain and spinal cord, the CNS is formed during embryonic development from the ectodermal neural plate. This tissue subsequently folds in on itself to form the neural tube. Early stages of CNS development occur as stem cells within the neural tube differentiate into increasingly specific cell fates. This results in a patterned neural tube as different regions of the forming CNS contain different types of cells. Thus, the differentiation steps must happen in a spatially distinct manner. One of the first neural tube patterning events happens along the rostral-caudal (R-C) tube axis a few weeks into development. This results in formation of the forebrain (FB), midbrain (MB), and hindbrain (HB) regions. Roughly at the same time, a similar kind of regionalization occurs along the dorsal-ventral (D-V) tube axis^1^.

Patterning is a complex and concerted process, and many of the mechanisms involved are not well understood. However, patterning mechanisms are likely to involve the regulation of gene expression and the presence of spatial concentration gradients of morphogens. The morphogen concentrations affect the gene regulatory mechanisms controlling the cell differentiation process. Therefore, cells choose different fates depending on where in the developing tissue they are located^2^.

Spatial concentration gradients of molecular signaling substances known to influence the expression of genes involved in key developmental pathways have been observed in both vertebrate^3^ and other embryos^4^. Such gradients can be established via diffusion or other mechanisms of inter-cellular transport^5^. Taken together, these experimental results suggest that patterning might indeed occur as the gene regulatory responses to morphogenic gradients.

**Fig. 1:**
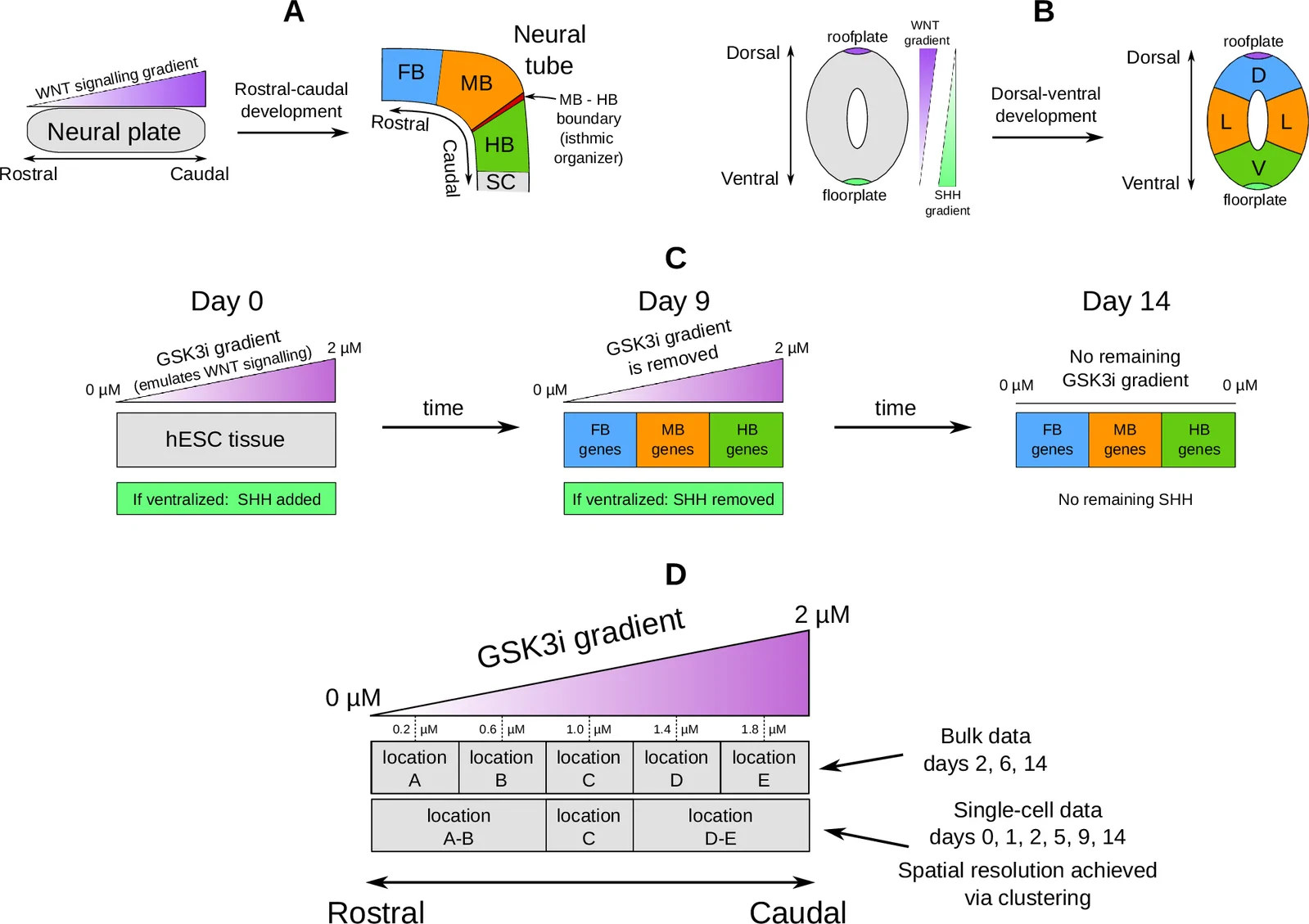
Schematic representation of early neural tube patterning in vivo (A, B) as well as in the in vitro MiSTR experiment (C, D). **A** The rostral-caudal patterning axis is established already at the neural plate stage as response to an early WNT signaling gradient from adjacent tissue. The isthmic organizer forms at the emerging midbrain- hindbrain boundary. **B** Opposing gradients of WNT and SHH from mainly the roof plate and floor plate contribute to regionalization in the dorsal-ventral direction, with a lateral (L) region forming in between dorsal (D) and ventral (V) regions. **C** Time-course of the MiSTR experiment, wherein a slab of human embryonic stem cell (hESC) tissue is patterned over a time span of multiple days. A GSK3 inhibitor (GSK3i) gradient emulates the in vivo rostral-caudal WNT signaling gradient, and is present during the first 9 days of the experiment. The emerging rostral-caudal pattern remains at day 14, 5 days after GSK3i removal. SHH is either not present at all, or present during days 0–9 to stimulate development of ventral cell fates. **D** The scheme for collecting cells for bulk and single-cell RNA sequencing (scRNA-seq) in MiSTR. Each location **A**–**E** from which cells were collected for bulk sequencing corresponds to a specific average level of GSK3i. Spatial resolution for cells collected for scRNA-seq at three locations **A**–**E** was achieved via data processing.

Wingless-Int (WNT), Fibroblast Growth Factor (FGF), and Sonic Hedgehog (SHH) are morphogen substances acting in the neural tube development. They are all encoded from genes with the same names. A WNT signaling gradient in the R-C direction has been shown to be important for inducing the FB-MB-HB regionalization^6^. WNT signaling is therefore also likely to play a role in development of the isthmic organizer (IsO) at the midbrain-hindbrain boundary (MHB). The isthmic organizer is then thought to act as a secondary source of both WNT and FGF morphogens^7,8^. Together with SHH, WNT also plays a role in D-V regionalization^9^. The key R-C and D-V neural tube patterning events are schematically illustrated in Fig. 1A, B.

Here, we use computational modeling to obtain a clearer picture of how the neural tube is patterned. We explain how genes being regulated by WNT, FGF, and SHH morphogen gradients can result in the R-C and D-V patterns, which are observed a few weeks into human development, Fig. 1A, B. We focus on irreversibility i.e., how developmental patterns can persist long after removal of the morphogen substances which induced them.

Computational studies of neural tube patterning in humans are challenging due to the scarcity of gene expression data that can be directly compared with simulation outcomes. Since in vivo experiments are not feasible, we propose a novel framework that leverages data from in vitro experiments involving the artificial patterning of differentiating human embryonic stem cells (hESCs). These data are used to develop computational single-cell models, which are then integrated into a 3D geometry to create a multiscale model for neural tube patterning.

Using our microfluidics-based device, MiSTR (microfluidic stem cell regionalization), we investigate both rostrocaudal (RC) and dorsoventral (DV) neural tube patterning^10^. Specifically, we analyze how a WNT signaling gradient drives early rostrocaudal regionalization in an elongated human embryonic stem cell (hESC) tissue, which serves as a model for the neural plate. Additionally, MiSTR is employed to study DV patterning: ventral cell fates are induced by adding Sonic Hedgehog (SHH) to the hESC tissue, while dorsal cell fates emerge in its absence.

To capture these processes, we collected both bulk and single-cell RNA sequencing (scRNA-seq) data from distinct regions of the tissue at multiple time points (Fig. 1D). This approach generated RNA-level time series datasets for genes critical to neural tube patterning.

We investigated how the dynamics of gene regulatory networks (GRNs), influenced by WNT, FGF, and SHH morphogens, drive the transcriptional regulation underlying early neural tube regionalization (Fig. 1A, B). Using gene expression data from MiSTR experiments, we optimized computational GRN models to explore the mechanisms that establish and maintain the isthmic organizer (IsO) at the midbrain-hindbrain boundary (MHB) and pattern the adjacent midbrain (MB) region. Capturing this recursive patterning process is critical, as morphogen gradients can induce secondary organizers, like the IsO, which further refine the initial pattern.

We began by analyzing scRNA-seq datasets from MiSTR experiments to confirm that the observed gene expression patterns align with in vivo data^11^ and to extract time-series information for model optimization. Our initial GRN model^12^ proved insufficient for MiSTR data, prompting us to develop a more complex model that incorporates both isthmic organizer dynamics and patterning into forebrain (FB), MB, and hindbrain (HB) regions.

To address dorsoventral (D-V) patterning, we evaluated a model from the literature^13^, but found it inconsistent with our MiSTR data. This led us to create a new D-V patterning model guided by our scRNA-seq results. Finally, we integrated our experimentally validated rostrocaudal (R-C) and D-V GRN models to simulate in vivo patterning of the anterior neural tube in a realistic 3D voxel geometry. This complete 3D model successfully reproduced R-C and D-V neural tube patterns observed during early development, with the isthmic organizer emerging at the correct position.

Here, we use MiSTR-derived gene expression dynamics to build GRN models for both the R-C and D-V axes and embed them in a realistic 3D neural tube geometry. The integrated framework reproduces tissue-level neural tube patterning, including forebrain, midbrain, hindbrain, dorsal, lateral, and ventral domains, and correctly positions the isthmic organizer at the midbrain-hindbrain boundary. Mechanistically, it includes an expanded R-C model capturing OTX2-GBX2 boundary formation and IsO dynamics, as well as a new D-V model in which GLI-mediated regulation and regional self-activation reproduce stable dorsal-lateral-ventral patterning. Together, this provides a framework for dissecting how morphogen-driven GRNs and tissue geometry shape early neural tube development. This is the first 3D neural tube model to correctly position the isthmic organizer and recapitulate patterning of the tube in both directions correctly, based on gene expression and morphogen dynamics.

## Results

### Time-resolved scRNA-seq gene expression data exhibits distinct and irreversible rostral-caudal and dorsal-ventral patterns

We began by analyzing co-expression patterns in data from in vitro MiSTR experiments (Fig. 1C). Each experiment ran for several weeks, with a glycogen synthase kinase 3 inhibitor (GSK3i) and Sonic Hedgehog (SHH) removed at day 9 to investigate whether the induced rostrocaudal (R-C) and dorsoventral (D-V) regionalization persisted in the absence of these signals. GSK3i, used in conjunction with neural induction factors, emulates the effects of canonical WNT signaling by disrupting the β-catenin destruction complex, of which GSK3 is a constituent^14^.

Our analysis focused on genes known to play key roles in neural tube differentiation and as distinct markers of specific cell fates. We optimized our models using scRNA-seq data, achieving improved temporal resolution compared to bulk sequencing data (Fig. 1D). The models were refined by comparing simulation outcomes with data from cells exposed to varying levels of GSK3i signaling during days 0–9.

However, the scRNA-seq data lacked inherent spatial resolution, as the position of each cell along the GSK3i gradient was unknown. To address this, we inferred the spatial origin of cells, categorizing them as originating from regions with low, intermediate, or high GSK3i levels (locations A-B, C, or D-E in Fig. 1D). This allowed us to generate scRNA-seq time series data for each of these three regions, mirroring the structure of the bulk sequencing data for location A-E (Fig. 1D). Cells were assigned to these regions using a data processing pipeline that included UMAP dimensionality reduction, followed by clustering based on known marker genes (see METHODS: Processing and analysis of experimental data).

To validate this positional reconstruction, we compared the inferred scRNA-seq regions with independent spatial information from MiSTR experiments. First, we showed in ref.^10^ that a controlled WNT/GSK3i gradient reproducibly generates progressive rostro-caudal patterning from forebrain to midbrain to hindbrain, including IsO-like characteristics. These transcriptomic patterns recapitulate early rostro-caudal neural plate development. To confirm regionally ordered expression of markers such as FOXG1, OTX2, FGF17, and HOXB1, and mapping MiSTR tissues to corresponding axes of the early human neural tube, we used our new temporal MiSTR atlas^11^. This is based on physical sub-dissection into five regions across the gradient axis together with CITE-seq hashtagging to preserve positional information. Together, these results support the use of marker-based clustering to reconstruct coarse positional domains in our scRNA-seq data. This approach reconstructs broad spatial regions with sufficient spatial resolution to distinguish broad R-C domains.

**Fig. 2:**
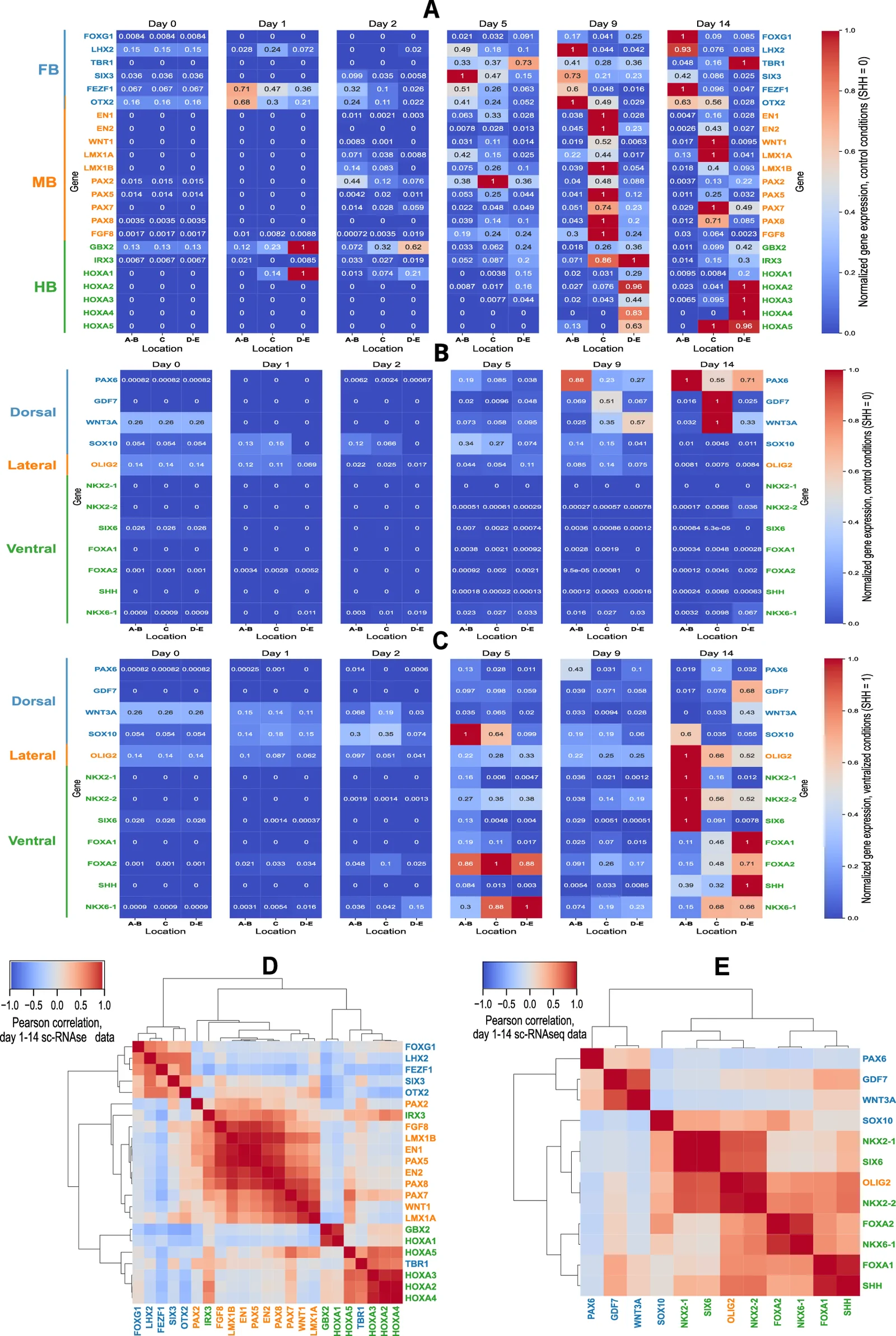
scRNA-seq gene expression datasets and clustering of genes relevant for rostral-caudal and dorsal-ventral patterning, with color-coded gene names. **A** scRNA-seq data for rostral-caudal genes. Note that OTX2 is classified as both an FB and an MB gene. **B** scRNA-seq data for dorsal-ventral genes under normal conditions (day 0–9 SHH is not present). **C** scRNA- seq data for dorsal-ventral genes under ventralized conditions (day 0–9 SHH is present). **D** Hierarchical clustering of the rostral-caudal scRNA-seq dataset in (**A**). The three top-level clusters are in close agreement with the FB, MB, and HB gene categories. **E** Hierarchical clustering of the dorsal-ventral scRNA-seq datasets in (**B** and **C**). The two top-level clusters are in close agreement with the dorsal and lateral/ventral gene categories.

Figure 2A presents the final single-cell RNA-seq gene expression profiles for core genes relevant to rostrocaudal (R-C) patterning. Gene names are color-coded based on their association with forebrain (FB), midbrain (MB), or hindbrain (HB) regions. Genes characteristic of FB localization are predominantly expressed in regions A-B, MB-specific genes in region C, and HB-associated genes in regions D-E (Fig. 2A). These expression patterns align with findings from previous in vitro patterning studies^15^.

Additionally, the spatio-temporal gene expression patterns observed in Fig. 2A demonstrate strong concordance with the R-C bulk sequencing dataset presented in Fig. S1E. This consistency between datasets validates our methodology for achieving spatial resolution in scRNA-seq data.

Notably, genes identified as markers of early R-C regionalization, such as: OTX2, FEZF1, GBX2, and HOXA1, exhibit high expression levels as early as days 1–2. In contrast, genes associated with later stages of R-C development, including FOXG1, EN1, and HOXA3-HOXA5, show significant upregulation only by day 9. Importantly, the overall expression patterns remain largely stable between days 9 and 14, despite the withdrawal of GSK3i at day 9 (Fig. 2B), suggesting irreversible effects of GSK3i exposure on human embryonic stem cells (hESCs).

To quantitatively assess the similarity of gene expression profiles, we computed the covariance between all spatio-temporal profiles depicted in Fig. 2A. A bottom-up hierarchical correlation clustering analysis was then performed on the resulting normalized covariance matrix (Fig. 2D; METHODS). The clustering results revealed three primary clusters that closely corresponded to the assigned FB, MB, and HB gene labels. Furthermore, early and late markers were segregated into distinct sub-clusters, exemplified by the separation of early HB markers (e.g., GBX2, HOXA1) from late HB markers (e.g., HOXA3, HOXA4, HOXA5).

The single-cell RNA-seq gene expression profiles for core genes involved in dorsoventral (D-V) patterning are illustrated in Fig. 2B (normal conditions) and Fig. 2C (ventralized conditions). Both datasets were jointly normalized to facilitate direct comparison between the two experimental conditions. A prominent observation in the D-V datasets is the absence of significant ventral gene expression under normal conditions, with these genes exhibiting marked upregulation exclusively during ventralization. This pattern underscores the essential role of Sonic Hedgehog (SHH) in driving differentiation toward ventral cell fates.

Consistent with this finding, dorsal markers generally display reduced expression under ventralized conditions compared to normal conditions, with SOX10 representing a notable exception. The lateral gene OLIG2, typically expressed in the intermediate region between dorsal and ventral domains, behaves similarly to ventral genes under ventralized conditions but shows higher expression levels than ventral genes under normal conditions. The expression patterns across locations A–E exhibit greater heterogeneity compared to the distinction between ventralized and non-ventralized conditions, with substantial variability observed among genes within the same category. Importantly, the clear demarcation between dorsal and ventral expression profiles persists through day 14, indicating that transient exposure to SHH induces lasting cellular changes.

Figure 2E presents correlation clustering of the normalized covariance matrices for the D-V gene datasets, performed analogously to the analysis of the R-C gene dataset in Fig. 2D. This clustering analysis reveals a distinct separation between dorsal and ventral gene expression profiles. Furthermore, the clustering distinguishes between rostrally and caudally expressed dorsal genes (e.g., PAX6, GDF7, WNT3A) and lateral + ventral genes (e.g., NKX2-1, SIX6, OLIG2, NKX2-2, FOXA2, NKX6-1, FOXA1, SHH).

In summary, the scRNA-seq datasets for both rostro-caudal and dorso-ventral patterning provide valuable insights into the dynamics of in vitro neural patterning. These datasets exhibit strong concordance with the R-C and D-V gene expression profiles observed during in vivo neural development. Critically, the high-resolution temporal and spatial information contained within these datasets facilitates the development of quantitative computational models of neural patterning. By systematically optimizing model parameters and comparing simulation outcomes with the empirically derived time-series data, we can refine our understanding of the underlying regulatory mechanisms.

### A rostral-caudal gene regulatory model containing a tri-stable network motif correctly reproduces patterning into forebrain, midbrain, and hindbrain regions

**Fig. 3:**
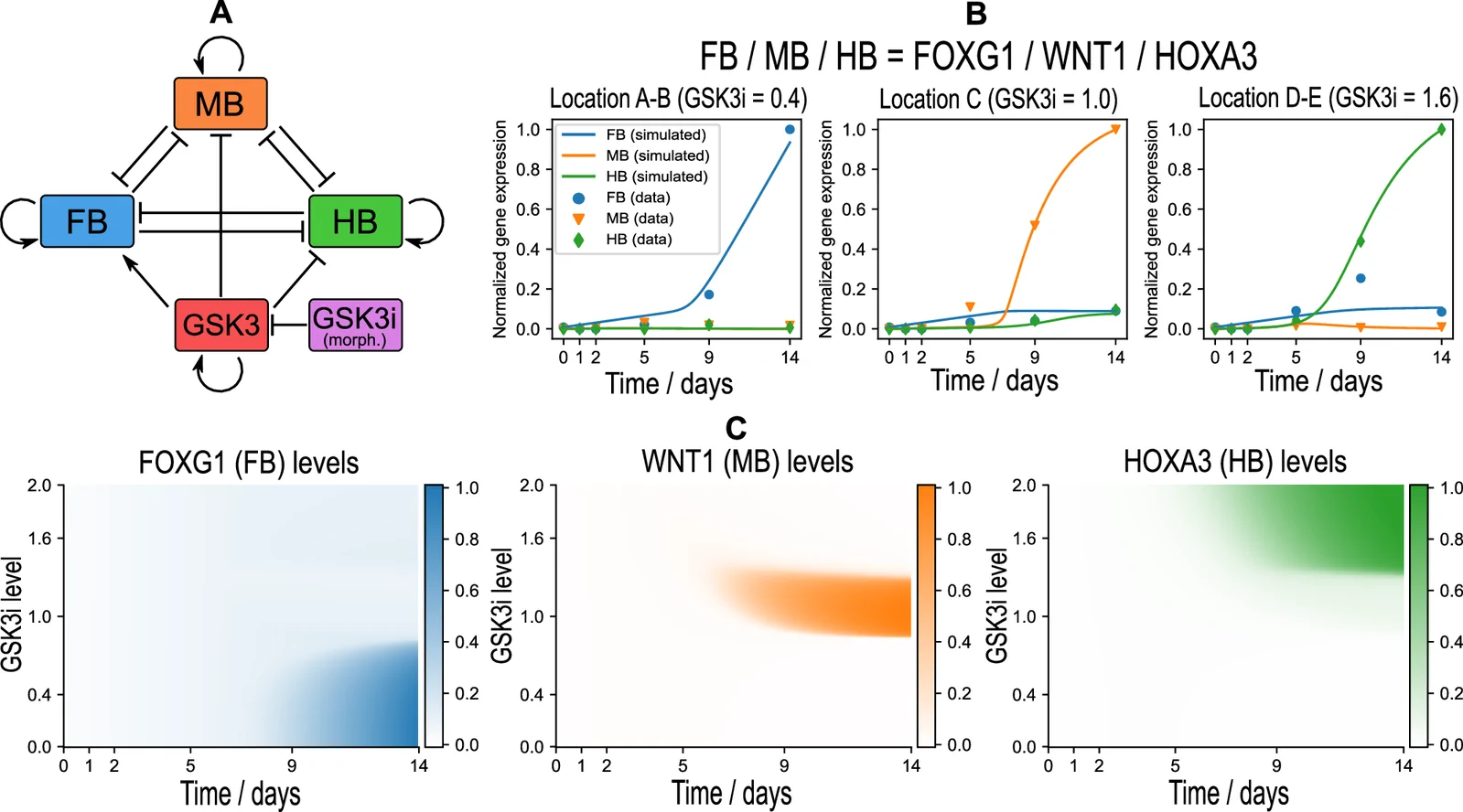
The tri-stable rostral-caudal patterning model. **A** The tri-stable rostral-caudal GRN. GSK3i emulates the in vivo morphogenic activity of early WNT signaling. Ordinary arrows (”→”) depict activation and blunt arrows (“→”) depict repression; we adhere to this convention throughout all GRN schematics shown in this paper. **B** Simulation of gene regulatory network (GRN) dynamics using the optimized parameter set for (FB, MB, HB) = (FOXG1, WNT1, HOXA3). The simulated trajectories are plotted alongside scRNA-seq data for the three GSK3 inhibitor (GSK3i) levels observed from day 0 to day 9, which were used to optimize the model. **C** GRN model dynamics for a continuous GSK3i input demonstrate that the model is capable of producing a continuous tri-stable pattern, which remains present after GSK3i is removed at day 9. The irreversibility of the induced FB, MB, and HB patterning arises from the toggle switch mechanisms embedded within the GRN model. These switches stabilize cell fate decisions, ensuring that once a specific regional identity is established, it is robustly maintained.

To advance gene regulatory patterning models, we first evaluated the strengths and limitations of existing frameworks using our MiSTR patterning data. We focused on the computational model proposed by Brambach et al. ^12^, which describes a gene regulatory network (GRN) for rostrocaudal (R-C) patterning (Fig. 3A). This model simulates the differentiation of forebrain (FB), midbrain (MB), and hindbrain (HB) regions through explicit inhibition of glycogen synthase kinase 3 (GSK3). Low GSK3i levels promote FB differentiation, while higher levels favor MB and HB fates, consistent with our observations (Fig. 1A). The FB, MB, and HB nodes in the model mutually cross-repress each other while reinforcing their own states, forming a “tri-stable” GRN composed of three bi-stable switch motifs. This toggle switch mechanism is known for its robustness in maintaining stationary states in GRNs^16^ and plays a critical role in differentiation processes^17,18^.

In the tri-stable model, the FB-MB-HB pattern emerges as follows:

- Low GSK3i levels: FB is predominantly induced, suppressing MB and HB via repression.
- Intermediate GSK3i levels: MB is predominantly induced, suppressing FB and HB via repression.
- High GSK3i levels: HB is predominantly induced, suppressing FB and MB via repression.

Simulations of the tri-stable GRN^12^ align with these dynamics and are consistent with gene expression data from earlier GSK3i-mediated in vitro patterning experiments^15^. However, the original model was optimized only against a single, final time point. Re-optimizing the model using our time-resolved MiSTR data (Fig. 2A) allows us to assess its accuracy in capturing the dynamics of FB, MB, and HB marker genes. Additionally, the removal of GSK3i at day 9 in our MiSTR experiments (Fig. 1C) enables us to test whether the tri-stable model can maintain the induced FB-MB-HB pattern after inhibitor withdrawal.

We optimized the parameters of an improved tri-stable model (METHODS: The tri-stable rostral-caudal model equations) against MiSTR data for all FB, MB, and HB gene combinations (Fig. 2A). For details on our general framework for simulating GRN dynamics and optimizing models from experimental data, see METHODS: Deterministic simulations of gene regulation via the Shea-Ackers formalism and METHODS: Optimization of models against experimental data.

Our optimized tri-stable model simulations closely matched the data for a wide range of FB, MB, and HB gene combinations (Fig. S3A-F). Figure 3B illustrates the simulation outcomes using the best-optimized parameters for a specific combination of scRNA-seq data: FOXG1 (FB), WNT1 (MB), and HOXA3 (HB). The model accurately reproduces the spatial expression dynamics observed experimentally, with FOXG1 highly expressed in rostral regions, WNT1 in central regions, and HOXA3 in caudal regions. Notably, the simulated tri-stable pattern persists after GSK3i removal at day 9. A sensitivity analysis of the optimized model further elucidated the impact of each parameter on model dynamics (Fig. S6A).

To assess the model’s generalization capability, we simulated gene expression dynamics across a continuum of GSK3i levels (0 to 2.0) to mimic the continuous GSK3i gradient in MiSTR (Fig. 1C). The model successfully generated a smooth FB → MB → HB pattern along the simulated gradient, which persisted after day 9 (Fig. 3C).

In summary, our optimized tri-stable model effectively predicts patterns consistent with MiSTR data, maintaining the FB-MB-HB pattern through toggle switch motifs even after GSK3i removal. This model provides a robust foundation for understanding basic R-C regionalization in response to a WNT signaling gradient.

### An expanded rostral-caudal model distinguishes between early and late patterning, and accurately captures isthmic organizer dynamics

The model in the previous section, while useful, inherently overlooks critical aspects of rostrocaudal (R-C) patterning, including the formation and maintenance of the isthmic organizer (IsO) as a secondary morphogen source, as well as the multiphasic nature of R-C patterning. Early genes (e.g., OTX2, GBX2) play distinct roles from later-expressed genes (e.g., FOXG1, HOXA3). To address these complexities, we developed a more detailed and mechanistically accurate GRN model for R-C patterning. This model aims to capture IsO dynamics, differentiate between early and late patterning phases, and retain the simplicity of the tri-stable model while maintaining strong agreement with MiSTR data.

To construct this expanded R-C model, we combined two complementary regulatory frameworks. We retained the tri-stable FB-MB-HB patterning motif proposed by Brambach et al., which was the topology that best fitted the experimental data. Both here and in Brambach et al. WNT/GSK3 signaling controls mutually repressive forebrain, midbrain and hindbrain gene modules, allowing stable regional specification along the rostro-caudal axis^12^. We then incorporated the experimentally established OTX2-GBX2 boundary mechanism, where anterior OTX2 and posterior GBX2 domains mutually repress each other to position the midbrain-hindbrain boundary (MHB)^19,20^. This boundary is closely linked to the emergence of the isthmic organizer, where WNT1 and FGF8 signaling contribute to maintenance of the MHB and induction of adjacent midbrain identity^19^. This resulted in a minimal, biologically motivated model that explains the observed MiSTR dynamics. Early WNT/GSK3 signaling first positions the OTX2-GBX2 boundary, after which organizer-derived WNT1 and FGF8 promote midbrain specification while OTX2- and GBX2-associated inputs contribute to later forebrain and hindbrain identity.

R-C patterning does not initially establish the three distinct FB, MB, and HB regions. Instead, the neural tube is first patterned into two broad domains: a rostral region (future FB + MB) characterized by high OTX2 expression, and a caudal region (future HB) marked by high GBX2 expression^21,22^. OTX2 and GBX2 not only serve as markers of these regions but also regulate each other via a toggle switch motif, defining the midbrain-hindbrain boundary (MHB)^23^. Following this initial regionalization, the IsO emerges at the MHB and acts as a secondary organizer. The IsO secretes morphogens such as WNT1 and FGF8, which refine the pattern by inducing MB fate in adjacent tissues^1,24^. MB is characterized by high expression of EN and PAX genes (e.g., EN1, EN2, PAX2, PAX5), while late FB and HB genes (e.g., FOXG1, LHX2, HOXA3-HOXA5) are expressed in the rostral and caudal regions, respectively^25–27^.

The early R-C patterning phase, corresponding to the first few days of MiSTR experiments, aligns with the OTX2 and GBX2 expression patterns observed in our data (Figs. 2A, S1E). The later R-C patterning phase, spanning days 5–14, involves the expression of IsO, FB, MB, and HB genes, consistent with literature. This alignment enabled us to use MiSTR data to guide the development of an expanded R-C patterning GRN model.

**Fig. 4:**
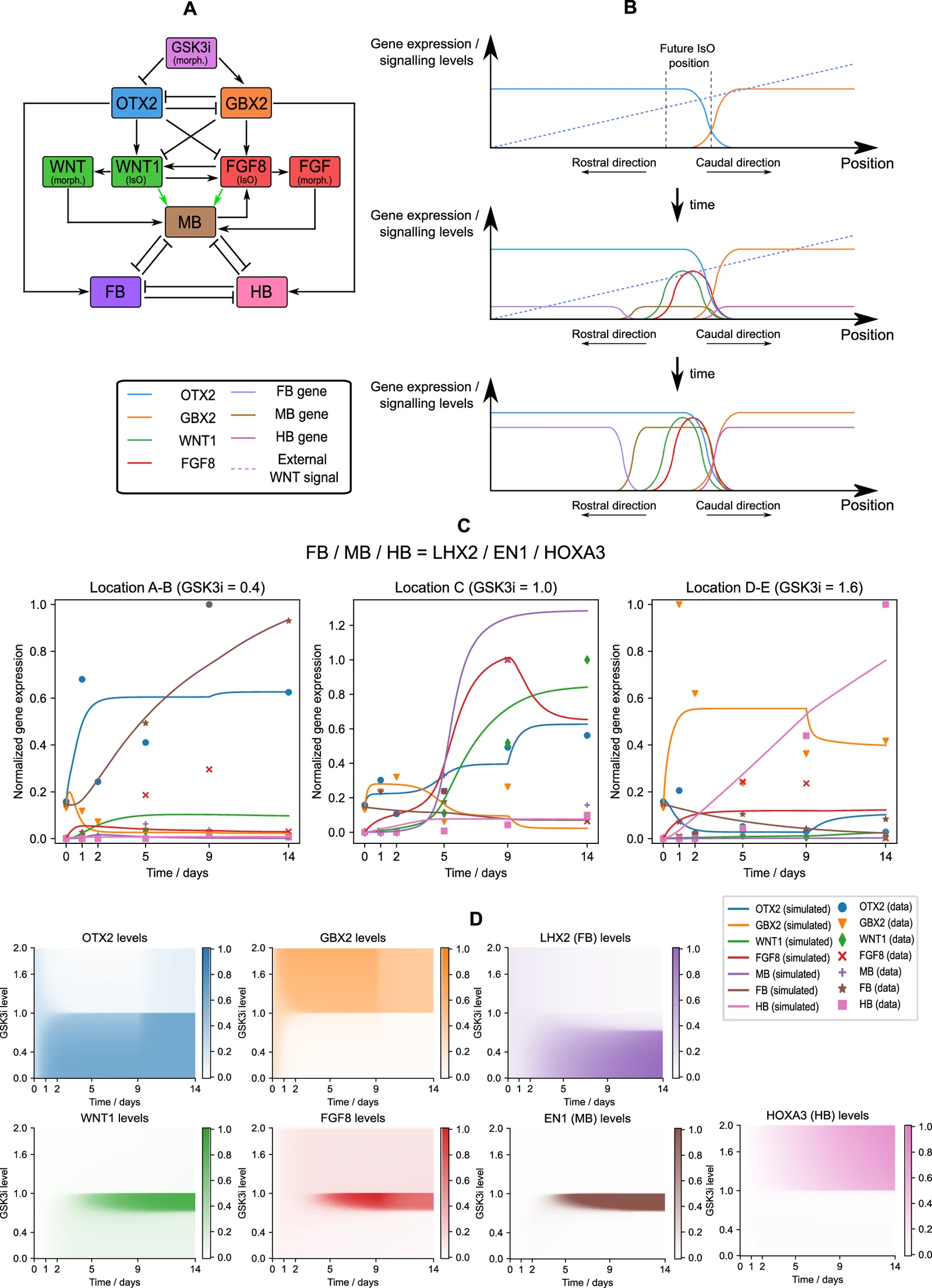
Expanded model capturing both isthmic organizer development and tri-stable rostral- caudal patterning. **A** The gene regulatory network (GRN) model is structured into two interconnected sub-networks: the upper sub-network (comprising OTX2, GBX2, WNT1, and FGF8) models the early development of the Isthmic Organizer (IsO). This sub-network captures how OTX2 and GBX2 respond to a rostral-caudal WNT signaling gradient, establishing the initial patterning cues. The lower sub-network (FB, MB, HB) represents a tri-stable model adapted from Fig. 3, where FB and HB identities are now directly induced by the earlier rostral and caudal genes OTX2 and GBX2, respectively. Meanwhile, MB specification is driven by WNT and FGF morphogens originating from the IsO. Importantly, MB feeds back into the IsO, a mechanism that may be crucial for sustaining IsO activity even after withdrawal of the external WNT signal. For the simulations shown in panels C and D, the model uses direct induction pathways (green arrows) from WNT1 and FGF8 to MB, rather than routing through the broader WNT and FGF morphogen nodes, to simplify model optimization. **B** This schematic illustrates the key developmental events underlying Isthmic Organizer (IsO) formation and rostral-caudal patterning, as modeled by the gene regulatory network (GRN) in (**A**). Initially, the OTX2-GBX2 boundary establishes the position of the midbrain-hindbrain boundary (MHB), which defines the caudal limit of the IsO. Following this, WNT1 and FGF8 are expressed just rostral to the MHB. Their signaling activity then promotes the development of the MB region adjacent to the IsO. Subsequently, FB and HB genes begin to be expressed rostral and caudal to the MB region, respectively, completing the rostral-caudal patterning process. **C** Simulations of the GRN model dynamics using the optimal parameter set for the markers (FB, MB, HB) = (LHX2, EN1, HOXA3). These simulations are plotted alongside scRNA-seq data across six time points (days 0, 1, 2, 5, 9, and 14) under three different levels of GSK3 inhibition (GSK3i). **D** GRN model dynamics with the same parameter set as in panel C, but under continuous GSK3i input. The model successfully generates an IsO region rostral to the MHB, as defined by the OTX2-GBX2 boundary. Additionally, it establishes a stable tri-regional pattern of FB, MB, and HB identities. Notably, these patterns persist even after withdrawal of GSK3i at day 9, indicating the robustness of the established patterning.

The OTX2-GBX2 toggle switch is essential for correctly positioning the MHB^23^. Experimental evidence supports the role of early WNT signaling in activating GBX2 and inhibiting OTX2^28,29^. We incorporated these insights into our expanded GRN model (Fig. 4A; STAR METHODS: Expanded rostral-caudal model), which captures early R-C regionalization, IsO formation, MB induction, and later FB and HB patterning. The model suggests that early WNT signaling regulates the OTX2-GBX2 switch, positioning the MHB.

The GRN governing IsO formation, MB induction, and WNT1/FGF8 dynamics is based on established studies^22,30^. For simplicity, EN and PAX genes, which share identical functional roles, were grouped into a single MB node. The tri-stable motif from the previous model was retained but adapted to represent MB and later FB/HB regions, with self-interactions removed as they were no longer necessary.

Figure 4B schematically illustrates the gene expression dynamics inferred from experimental observations. To validate the model, we optimized its parameters against MiSTR scRNA-seq data and simulated gene expression across a continuum of GSK3i levels. Simulations (Fig. 4C) demonstrate that OTX2 and GBX2 establish early rostral and caudal regions, respectively, with the IsO and MB genes (WNT1, FGF8, EN1) induced in location C. This pattern persists after GSK3i removal at day 9, reflecting the irreversibility of the OTX2-GBX2 toggle switch^31^. FB and HB genes are induced in locations A-B and D-E, respectively, consistent with MiSTR data.

A sensitivity analysis (Fig. S6B) revealed that the model is most sensitive to parameters involving OTX2, GBX2, and their regulation by GSK3i, underscoring their central role in positioning the IsO. Simulations across a GSK3i gradient (Fig. 4D) further confirmed the model’s ability to recapitulate the spatio-temporal patterns observed experimentally.

In summary, our expanded GRN model integrates IsO formation^22^ and R-C patterning^12^ into a unified framework. The model demonstrates that WNT signaling positions the MHB by regulating OTX2 and GBX2, leading to irreversible IsO maintenance and correct FB, MB, and HB regionalization. Simulations align with MiSTR scRNA-seq data, capturing:

- Early R-C regionalization,
- IsO positioning and maintenance,
- MB induction by the IsO,
- Irreversible maintenance of the IsO,
- Correct spatial induction of late FB and HB genes.

This model provides a robust and minimal representation of early R-C neural tube patterning, rooted in experimental observations and mechanistic insights.

### A new dorsal-ventral model developed from our scRNA-seq data produces correct, sustained patterns

In the experimentally validated framework of dorsoventral (D-V) neural tube patterning, Sonic Hedgehog (SHH), secreted by the notochord and ventral floor plate, functions as a long-range morphogen^32^. The resulting ventral-to-dorsal SHH gradient orchestrates D-V patterning through modulation of the GLI transcription factors: GLI1, GLI2, and GLI3, whereby high SHH concentrations induce ventral cell fates^33^. Concurrently, a WNT gradient emanating from the roof plate antagonizes the ventralizing effects of SHH^9^.

Neuronal subtypes along the D-V axis are broadly categorized into dorsal (D), ventral (V), and lateral (L) domains, with L types positioned intermediate to D and V (Fig. 1B). These regions are molecularly defined by the high expression of the transcription factors NKX2-2, PAX6, and OLIG2, respectively, which interact within a gene regulatory network (GRN) to reciprocally regulate each other^13^.

To develop a gene regulatory network (GRN) model of dorsoventral (D-V) patterning, we optimized all model components using experimental data, building upon the framework proposed by ref.^12^. Our objective was to refine this model to generate a D-L-V pattern consistent with the schematic in Fig. 1B and aligned with our MiSTR dataset.

**Fig. 5:**
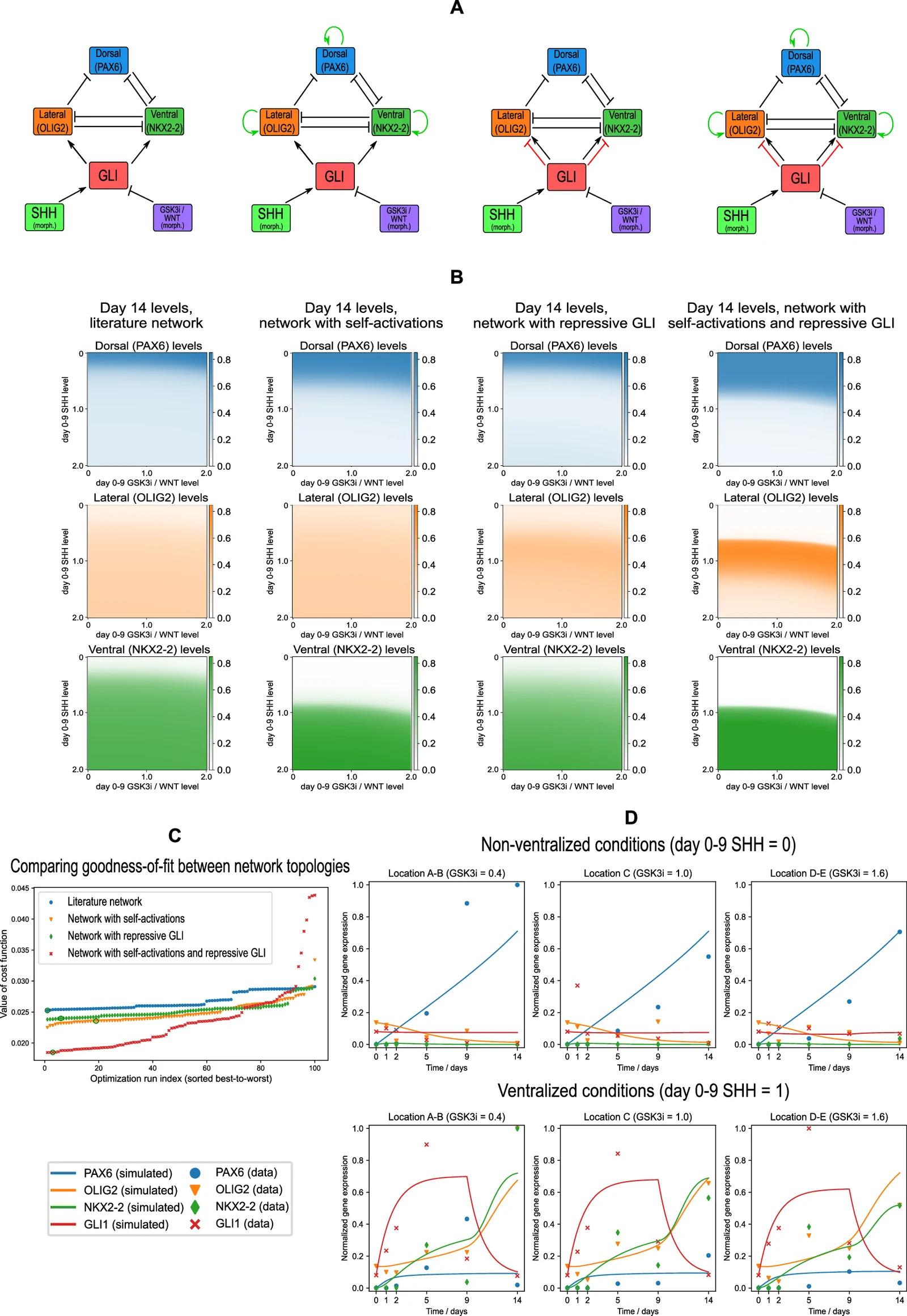
The dorsal-ventral patterning models. **A** Four gene regulatory network (GRN) model topologies evaluated in this study. From left to right: the literature topology^13^; a topology with added self-activations (green) for the dorsal, ventral, and lateral nodes; a topology where GLI both activates and represses (red) the lateral and ventral nodes; and a combined topology incorporating both modifications (green and red connections). **B** Simulated day 14 expression levels of dorsal, ventral, and lateral GRN nodes across a continuous 2D input space of WNT and SHH levels from days 0 to 9. Simulations use parameter sets corresponding to the circled cost function values in (**C**), with each plot aligned below its respective topology in (**A**). Only the combined-modification topology produces distinct spatial dominance of dorsal, ventral, and lateral nodes. **C** Goodness-of-fit comparison across the four topologies, shown as sorted cost function values (lower is better) from 100 optimization runs per topology. The combined-modification topology consistently outperforms the others, with circled values indicating the parameter sets used in (**B**). **D** Model dynamics simulated using the optimal parameter set for the combined-modification topology, overlaid with scRNA-seq data for three GSK3i levels and two SHH levels. Dorsal dominance occurs without SHH (no GLI induction), while ventral dominance over lateral nodes requires low WNT levels in the presence of SHH, as higher WNT dampens GLI induction by SHH.

The original D-V model from ref.^12^ is depicted in the leftmost panel of Fig. 5A. We evaluated its performance by optimizing its parameters against MiSTR data, as detailed in the METHODS section. Simulations were conducted to assess the expression levels of D, L, and V genes at day 14 across a range of Sonic Hedgehog (SHH) and glycogen synthase kinase 3 inhibitor (GSK3i)/WNT concentrations. Notably, SHH and GSK3i/WNT were present only during the initial 9 days of the simulation. For the model to replicate an in vivo-like dorsal-lateral-ventral pattern, dorsal gene expression should peak at low SHH/high WNT levels, ventral gene expression at high SHH/low WNT levels, and lateral gene expression at intermediate levels.

However, the resulting day 14 expression distribution, shown in the leftmost panel of Fig. 5B, failed to produce a qualitatively accurate pattern. Specifically, the model did not generate a distinct lateral (L)-dominant region between the dorsal (D)-dominant and ventral (V)-dominant regions, a feature observed in vivo^13^.

To address this limitation, we modified the model’s topology in three distinct ways and re-optimized each variant against the MiSTR data. Our goal was to identify a topology capable of generating a qualitatively accurate day 14 pattern while accurately capturing the MiSTR data dynamics. During optimization of the original model, we observed that either the L or V genes did not sustain expression following SHH removal at day 9. To mitigate this, we first tested a topology incorporating self-activations for the D, L, and V nodes (Fig. 5A, second panel). Additionally, the optimized model dynamics rarely aligned with the MiSTR data at days 5 or 9. To resolve this, we introduced flexibility into the GLI effects on V and L nodes, allowing GLI to function as either an activator or a repressor (Fig. 5A, third panel). Finally, we considered a combined topology featuring both self-activations and GLI repressions (Fig. 5A, rightmost panel).

Simulations using the optimized parameters for the model with added self-activations did not yield a qualitatively accurate day 14 D-L-V pattern (Fig. 5B, second panel). Similarly, the model with repressive GLI did not produce a qualitatively accurate pattern (Fig. 5B, third panel). However, simulations with the model incorporating both self-activations and GLI repressions resulted in a qualitatively accurate pattern (Fig. 5B, rightmost panel), characterized by a distinct L-dominant region at intermediate SHH levels. Variations in WNT levels had a lesser impact on the day 14 pattern compared to SHH, although higher WNT levels expanded the D-dominant region, consistent with previous findings^9^.

In comparison to the MiSTR data, the model featuring both self-activations and GLI repressions demonstrated superior performance across a substantial proportion of optimization runs (Fig. 5C). The full dynamics of the best-performing model, utilizing the parameter set that produced the rightmost day 14 pattern in Fig. 5B, are illustrated in Fig. 5D. Under non-ventralized conditions (top panel), only the D gene exhibited significant expression in both the model and data, as GSK3i/WNT effectively repressed GLI in the absence of SHH, thereby suppressing the V and L nodes. Conversely, under ventralized conditions (bottom panel), the dorsal gene was repressed by the L and V genes, which were activated by GLI in response to SHH. Importantly, both L and V genes maintained high expression levels at day 14, persisting after SHH removal at day 9. V genes dominated at low GSK3i/WNT levels, while L genes prevailed at high GSK3i/WNT levels, consistent with the pattern depicted in Fig. 1B.

Sensitivity analysis of the optimized D-V model showed that parameters associated with the lateral and ventral nodes had the strongest influence on model performance (Figure S6C). Among the newly introduced interactions, this included the self-activation terms that stabilize induced L and V expression after SHH withdrawal, as well as GLI-mediated regulatory terms that tune the balance between lateral and ventral fate. This supports the topology comparison in Fig. 5B, C, where neither self-activation nor repressive GLI alone was sufficient to generate the desired D-L-V pattern, whereas their combination improved both pattern formation and agreement with MiSTR data.

Our refined gene regulatory network (GRN) model for dorsoventral (D-V) patterning incorporates self-activations for dorsal (D), lateral (L), and ventral (V) nodes, alongside a repressive function of GLI. This enhanced model effectively generates a qualitatively accurate D-L-V pattern by day 14. The inclusion of self-interactions ensures the persistence of induced gene expression patterns following the withdrawal of Sonic Hedgehog (SHH) at day 9. Furthermore, the introduction of repressive GLI activity on D and V nodes reflects the complexity of their regulatory mechanisms, which is essential for achieving biologically plausible patterning outcomes. The model’s dynamics robustly capture the sustained expression of D, L, and V genes, thereby providing a comprehensive representation of D-V patterning.

### 3D simulations with expanded rostral-caudal and dorsal-ventral models generate realistic in vivo-like patterns

We developed refined gene regulatory network (GRN) models for rostrocaudal (R-C) and dorsoventral (D-V) patterning, both of which align with MiSTR data and accurately reproduce gene expression patterns across varying morphogen signaling levels. To evaluate the capacity of these models to generate biologically plausible R-C and D-V patterns in a three-dimensional context, we conducted patterning simulations within a realistic 3D voxel geometry.

Our simulations assumed that spatial gradients of morphogens drive patterning in the 3D geometry, with the temporal evolution of R-C and D-V GRNs in each voxel influenced by local morphogen concentrations. We simulated GRN dynamics over 14 days using optimized parameter sets, with initial conditions derived from MiSTR scRNA-seq datasets. A detailed description of the simulation methodology is provided in the METHODS section.

**Fig. 6:**
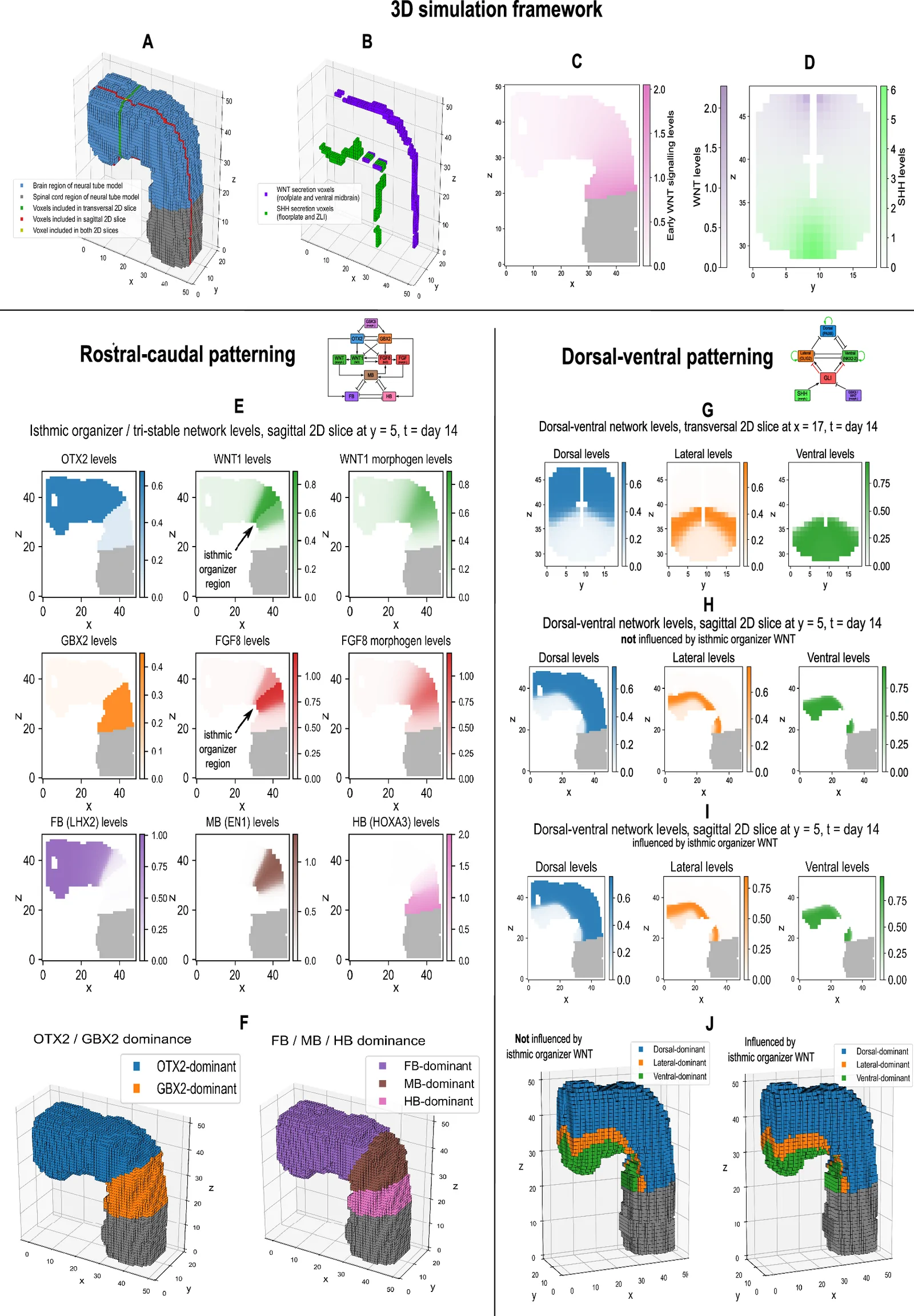
The 3D voxel simulation framework, together with results of simulating rostral-caudal and dorsal-ventral patterning in 3D. **A** Neural tube voxel model, including 2D transverse and sagittal slices used to present simulation results. The gray region represents the spinal cord, which is not modeled in this study. **B** Voxels designated as WNT and SHH secretion sources for dorsal-ventral patterning. **C** Early WNT signaling gradient driving rostral-caudal patterning, visualized in the sagittal slice from (**A**). **D** Dorsal-ventral patterning gradients of WNT and SHH, resulting from diffusion from the voxels in (**B**), shown in the transverse slice from (**A**). **E** Day 14 patterns of the full network model (IsO and tri-stable) with WNT and FGF morphogen nodes. This results in a broad organizer region. **F** Day 14 3D surface patterns of the rostral-caudal model, illustrating the spatial dominance of OTX2/GBX2 and FB/MB/HB gene groups, which exhibit nearly mutually exclusive expression profiles. **G** Day 14 dorsal-ventral model patterns in the transverse 2D slice. **H** Day 14 dorsal-ventral model patterns in the sagittal 2D slice. **I** Day 14 dorsal-ventral patterns in the sagittal slice, integrating WNT1 from the rostral-caudal model (**E**) as an extra input. This reveals a slight expansion of the dorsal domain near the tube bend due to isthmic organizer signaling. **J** Day 14 3D surface dominance of dorsal, ventral, and lateral nodes, showing that coupling the models via WNT1 (**E**) subtly expands the dorsal region only at the tube bend.

The 3D simulation framework is illustrated in Fig. 6A-D. The voxel geometry (Fig. 6A) and the WNT/SHH secretion voxels (Fig. 6B) were adapted from Brambach et al. ^12^. The spinal cord (gray voxels) was excluded from simulations, as its patterning process is distinct from that of the forebrain (FB), midbrain (MB), and hindbrain (HB)^34^. For visualization, we primarily analyzed patterns along transversal (green) and sagittal (red) 2D slices within the 3D geometry. Figure 6C depicts the early R-C WNT signaling gradient, which drives R-C patterning^6^, while Fig. 6D shows the WNT/SHH gradient driving D-V patterning. Both morphogen gradients were modeled to persist from day 0 to day 9, consistent with the MiSTR experimental conditions, although this duration does not fully replicate in vivo dynamics. Further details on gradient modeling are provided in the METHODS section.

We first evaluated R-C and D-V patterning independently, beginning with the R-C model. The 3D simulations utilized the full GRN depicted in Fig. 4A, incorporating diffusing WNT and FGF morphogens. The results of the R-C simulations are presented as a distribution of day 14 gene expression levels in the sagittal 2D slice (Fig. 6E). To visualize the OTX2-GBX2 boundary and the relative sizes of the FB, MB, and HB regions, we also mapped the dominant gene in each voxel at the surface of the 3D geometry (Fig. 6F). The observed patterns correspond to those in Fig. 4D, with a distinct OTX2-GBX2 boundary at the bend of the tube, IsO gene expression around this boundary, and overlap between the MB region and the IsO. Notably, the model predicts a more rostral expression of WNT1 within the IsO compared to FGF8, which is expressed more caudally. This prediction is supported by experimental observations of the IsO^35^. Given the identical parameters for WNT and FGF morphogens, the observed spatial asymmetry likely arises from differences in the relative strengths of GRN connections for WNT1 and FGF8.

For D-V patterning, simulated independently of R-C patterning, the resulting pattern is shown in the transversal 2D slice in Fig. 6G. This pattern is consistent with Fig. 1B and aligns with both in vivo and in silico results from Balaskas et al. ^13^. It should be noted that Balaskas et al. validate their model using non-human vertebrate data, including mouse embryo expression patterns, therefore mainly providing qualitative support through this comparison. Figure 6H demonstrates that the relative sizes of the D-V regions vary along the R-C axis, potentially due to the differential density of SHH secretion voxels along the R-C axis (Fig. 6B), the ventral midbrain source of WNT, and the influence of the curved geometry on the shapes of the SHH and WNT gradients.

Finally, we hypothesized that WNT morphogens secreted from the IsO might influence the D-V pattern. To investigate this, we simulated a fully coupled model of simultaneous R-C and D-V patterning. In this model, the combined effect of the WNT gradient from Fig. 6D and the dynamically evolving WNT gradient from the IsO was considered to impact GLI in the D-V GRN. The resulting pattern is shown in the sagittal slice in Fig. 6I, revealing a subtle but discernible effect on the overall pattern at the bend of the tube, where the IsO is located. Compared to Fig. 6H, the D region exhibits a slight expansion at the expense of the L and V regions. This effect is further illustrated in Fig. 6J, which maps the dominant genes in each voxel at the tube surface.

Our 3D patterning simulations utilized optimized rostrocaudal (R-C) and dorsoventral (D-V) gene regulatory networks (GRNs) within a realistic neural tube geometry, enabling the generation of R-C and D-V patterns consistent with in vivo observations. Notably, the isthmic organizer (IsO) emerged autonomously in its correct anatomical position, demonstrating the model’s capacity to recapitulate key developmental processes. Additionally, the model predicted an asymmetry in the distribution of WNT1 and FGF8 within the IsO, a finding that aligns with experimental validation.

A significant outcome of our simulations is the ability of both the R-C and D-V components of the model to irreversibly maintain their respective patterns at day 14, five days after the withdrawal of morphogen gradients (Fig. 6C, D). By integrating the R-C and D-V 3D models, we further demonstrated the utility of our 3D simulation framework for exploring hypotheses related to potential coupling mechanisms between patterning processes in different anatomical directions. This integrated approach provides a robust platform for investigating the interplay between R-C and D-V patterning during neural tube development.

## Discussion

In this study, we developed gene regulatory network (GRN) models of neural tube patterning by integrating microfluidic stem cell regionalization (MiSTR) in vitro patterning data with in silico modeling. The combination of in vitro and in silico approaches enables the investigation of complex patterning phenomena that are challenging or even impossible to study directly in vivo. The MiSTR platform provides a highly controlled environment for studying tissue patterning by morphogen gradients, allowing the collection of gene expression data across multiple time points and spatial positions along these gradients. This in vitro system successfully induced rostral-caudal and dorsal-ventral patterns that closely resemble in vivo gene expression dynamics and were maintained for several days after morphogen withdrawal under the tested MiSTR conditions. By using these experimental data, our in silico models offer a powerful framework for dissecting the mechanisms underlying neural tube patterning.

A limitation of our approach is that the reconstructed A-B, C, and D-E regions capture broad positional domains rather than all spatial heterogeneity within the MiSTR tissue. Finer variation, including transitional states, local differences in morphogen response, or asynchronous differentiation, may therefore not be explicitly represented in the GRN models. While the simulations reproduce the main qualitative R-C patterning dynamics, they do not match every expression value in Figs. 3B and 4C. These deviations likely reflect that the GRN models are simplified representations of heterogeneous in vitro data, using a limited number of regulatory nodes and parameters. The models should therefore be interpreted as capturing the dominant patterning logic rather than all quantitative variation in the scRNA-seq data.

Direct comparison between model outputs and in vivo single-cell expression is limited by the scarcity of early human neural tube datasets with matching temporal and spatial resolution. Existing human fetal brain atlases provide useful references, but generally cover broader or later developmental windows than the MiSTR time course^11,36,37^. Mouse embryonic datasets offer higher temporal resolution, but are less direct comparators due to species differences and developmental timing^38^. We therefore use available in vivo data mainly to support qualitative marker-domain consistency, rather than as direct point-wise targets for model fitting.

We initially assessed a simple rostral-caudal GRN model^12^, which successfully maintained an induced forebrain-midbrain-hindbrain (FB-MB-HB) pattern after removal of the WNT-emulating GSK3i morphogen. This maintenance of regional identity, mediated by toggle switches in the GRN topology, suggests a plausible mechanism for stabilizing rostral-caudal patterns after morphogen withdrawal. However, the predicted reversibility could be challenged by in-vitro limitations such as GSK inhibition potentially failing to completely wash off, and therefore the possibility of pathway activation still being affected by the drug. Nevertheless, our framework is not developed to be an overfitted in-vivo representation. Although the simplicity of this tri-stable model still limited its capacity to capture critical details of rostral-caudal patterning, particularly the formation and maintenance of the isthmic organizer (IsO).

To address these limitations, we developed an expanded rostral-caudal GRN model that integrates the tri-stable topology with a toggle switch motif between the rostral OTX2 and caudal GBX2 genes. This switch is pivotal in positioning the midbrain-hindbrain boundary (MHB) and, consequently, the IsO^30^. Experimental studies^28,29^ suggest that the initial rostral-caudal WNT gradient acts as an input to the OTX2-GBX2 switch, ensuring the correct positioning of the MHB. Feedback mechanisms between the IsO genes WNT1 and FGF8 and midbrain (MB) genes further stabilize the IsO in a well-defined position. Our optimized expanded model accurately predicted the sequential steps of early and late R-C patterning and the precise localization of the IsO, demonstrating strong alignment with experimental data.

For dorsoventral patterning, we utilized an established GRN model^13^ and found that it failed to sustain a reasonable dorsal-lateral-ventral pattern at day 14 of patterning or reproduce the irreversibility observed in MiSTR data. To rectify this, we refined the model to include self-activations and repressive GLI actions, which enabled it to maintain a biologically plausible dorsoventral pattern after morphogen withdrawal.

A key insight from our study is the critical role of morphogen gradients in inducing specific patterns, while the GRN topology is essential for maintaining these patterns after morphogen withdrawal. We extended our analysis to a realistic 3D voxel geometry, where we simulated the dynamic induction of diffusible IsO morphogens WNT1 and FGF8. Our 3D simulations revealed an asymmetry in the expression of these morphogens within the IsO, with WNT1 predominantly expressed in the rostral end and FGF8 in the caudal end, consistent with biological observations^1^. This result underscores the capability of our model to capture nuanced, experimentally validated details of IsO dynamics that are difficult to study in vivo.

In simulating dorsoventral patterning in 3D, we observed a sustained dorsal-lateral-ventral pattern post-morphogen removal, with regional sizes varying along the rostral-caudal axis. Although the influence of WNT from the IsO on dorsoventral patterning was modest, our 3D framework demonstrated its utility in exploring hypotheses about the coupling between patterning processes along different neural tube axes.

Overall, our GRN models represent significant advancements over previous efforts. They were optimized against scRNA-seq data, providing a mechanistic explanation for the irreversible maintenance of patterns induced by transient morphogen signaling. Importantly, our rostral-caudal model captures the emergence of a secondary morphogen source, the IsO, highlighting the model’s ability to elucidate complex biological phenomena that are experimentally inaccessible. Our 3D simulation framework further proved instrumental in investigating hypotheses about the interplay between patterning along different axes and the influence of morphogen dynamics on patterning processes.

While we acknowledge that neural tube patterning involves additional layers of complexity such as cell division, tissue growth, and non-transcriptional gene regulation, our transcriptional models successfully capture many essential features of these processes. Moving forward, our systems biology approach can be further refined with data from advanced in vitro patterning experiments producing complementary experimental data, offering deeper insights into the mechanisms underlying neural tube patterning.

## Methods

### MiSTR culture, differentiation, and RNA-seq data generation

Experimental generation of the MiSTR scRNA-seq datasets followed the protocol described by Rathore et al. ^11^, summarized here for reproducibility. MiSTR tissues were generated from H9 human embryonic stem cells (hESCs; hPSCReg: WAe009-A, RRID: CVCL_9773, WiCell), following the previously established MiSTR protocol with minor adaptations^10,11^. hESCs were maintained in StemMACS iPS Brew XF medium on Matrigel-coated plates (20 µg/cm²) and dissociated using 0.5 mM UltraPure EDTA before MiSTR seeding^11^. Cells were seeded onto a phenol-red-free Matrigel bed on a polydimethylsiloxane (PDMS) bottom layer in iPS Brew medium supplemented with ROCK inhibitor, assembled into the MiSTR device, and incubated overnight at 37 °C^11^.

MiSTR differentiation was performed under continuous medium flow at 160 μl/h using neural progenitor medium (NPM), consisting of a 1:1 mixture of DMEM/F12 and NeuroMedium supplemented with N2 supplement (1:200), NeuroBrew-21 without vitamin A (1:100), GlutaMAX (1:200), SB431542 (10 μM), and recombinant human Noggin (100 ng/ml)^10,11^. Rostro-caudal patterning was induced from day 0 to day 9 using a microfluidic CHIR99021/GSK3i gradient generated by two syringes containing NPM with or without GSK3i^10,11^. For ventralized rostro-caudal MiSTR conditions, SHH-C24II (200 ng/ml) and purmorphamine (0.5 μM) were added during the patterning phase^10,11^. On day 9, the medium was changed to basal NPM without GSK3i, SHH, or purmorphamine, and cultures were maintained until day 14^10,11^. Only MiSTR data up to day 14 were used in this study.

The scRNA-seq datasets used here were collected from R/C dorsal and R/C ventral MiSTR tissues at days 0, 1, 2, 5, 9, and 14. Previously generated libraries from Rifes et al. were used for days 0, 1, 2, and 14^10^, while additional MiSTR tissues were processed using the same experimental approach as described by Rathore et al.^11^. For day 5 and day 9 tissues, MiSTR samples were dissected along the rostro-caudal gradient axis into five regions, A–E^11^. One half of each tissue was used for qRT-PCR quality control, while the remaining half was dissociated using the Neural Dissociation Kit (P), cryopreserved in CryoStor CS10, and stored at −150 °C^11^. After quality control, cells from two to three biological replicates per time point, MiSTR model, and region were thawed, pooled in equal proportions, and labelled with region-specific TotalSeq-A hashtag antibodies^11^. Cells from all five regions were then pooled, and ~25,000 cells were loaded onto a 10x Genomics lane using Chromium v3.1 chemistry^11^.

Raw cDNA libraries were aligned to the GRCh38-2020 human reference genome using CellRanger, and hashtag libraries were processed using CITE-seq-Count to generate hashtag oligo count matrices^11^. The resulting gene expression count matrices were then used for the downstream processing and model-input generation described below. Bulk RNA expression data used for comparison were obtained from regions A–E of 36 independent control MiSTR runs at days 2, 6, and 14^10^.

### Processing and analysis of experimental data

Raw single-cell RNA sequencing (scRNA-seq) gene expression data from MiSTR experiments were processed to generate time series (Fig. 2A, B, and D) for use in optimizing the gene regulatory network (GRN) models. Similarly, raw bulk gene expression data were processed to produce comparable time series (Fig. S1E). The detailed processing steps are described below.

#### Processing steps for bulk gene expression data

Gene expression levels, E, derived from bulk measurements of cells in a specific MiSTR experiment run, can be structured as a three-dimensional array, E_g,t,l_. Here, the indices g, t, and l denote a specific gene, time point, and spatial location (A–E, as shown in Fig. 1D), respectively. Data were collected on days 2, 6, and 14 for each experimental run. For this analysis, bulk gene expression data from 36 independent MiSTR experiment runs, which were conducted under control conditions (without added SHH) and were averaged for a curated set of genes relevant to early neural tube development (as displayed in Fig. 2A). All bulk data processing was performed using Python 3 with the NumPy library^39^.

The mean expression levels $\bar{E}$ across all N=36 experiment runs were calculated via an element-wise average of the corresponding *E*, s $\bar{E}=\frac{1}{N}\mathop{\sum }\limits_{n=1}^{N}{E}^{(n)}$. To create time series, $\bar{E}$ was normalized across time t and space l, separately for each gene, resulting in the final, fully processed data array ${\bar{E}}^{({\rm{norm}}.)}.$ The normalization was made by dividing all expression levels for a particular gene by the maximum level ${\bar{E}}_{\max }$ of that gene across all times and locations: ${\bar{E}}^{({\rm{norm}}.)}=\bar{E}/{\bar{E}}_{\max }$.

This normalization scales the expression levels to the interval [0, 1], facilitating direct comparison of the spatio-temporal dynamics of different genes within a unified heatmap or plot. The normalized bulk gene expression time series are presented in Figure S1E.

#### Processing steps for scRNA-seq gene expression data

During the MiSTR experiments, single-cell RNA sequencing (scRNA-seq) measurements were conducted on days 0, 1, 2, 5, 9, and 14 under both control and ventralized conditions. For each time point and condition, the scRNA-seq dataset is represented as a count matrix C_gc_, where rows g correspond to genes and columns c correspond to individual cells. Each entry in the matrix signifies the number of detections of a particular gene’s mRNA within a cell during sequencing.

The count matrices were pre-processed in R using the Seurat package^40^ to correct for confounding factors, including batch effects and cell-cycle heterogeneity. Principal component analysis (PCA) was performed, followed by clustering using a combination of shared nearest neighbors (SNN) and the Louvain algorithm^41^. To ensure robust clustering, only the 2000 most variable genes for each time point were selected, thereby minimizing the formation of spurious clusters.

We further processed the count matrices to generate time-series data for clustering and GRN model optimization. To align with the structure of bulk data time series, the created time series should contain gene expression levels for multiple distinct spatial locations within the MiSTR setup. These locations correspond to varying levels of GSK3i along its gradient.

The spatial origin of each cell in C_gc_ was inferred from its gene expression profile for known marker genes. The spatial grouping of cells was performed in R, following this procedure:

First, the 2000-dimensional data was reduced to two dimensions using the Uniform Manifold Approximation and Projection (UMAP) algorithm^42^, which preserves both local and global data structure more effectively than other dimensionality reduction techniques such as t-SNE. As an example, Fig. S1A illustrates the cell clusters in 2D UMAP space for the day 9 non-ventralized dataset.

Since forebrain (FB), midbrain (MB), and hindbrain (HB) regions of the neural tube are characterized by high expression levels of specific marker genes at particular times, clusters in Fig. S1A could be assigned FB, MB, or HB identities. For instance, Fig. S1B-D shows the distribution of expression levels for FEZF1 (FB marker), WNT1 (MB marker), and HOXA2 (HB marker) for the day 9 non-ventralized dataset. The rostral-caudal axis can be inferred to extend approximately from right to left in this UMAP visualization. Consequently, cluster 3 in Figure S1A can be identified as predominantly consisting of midbrain cells, likely originating from the central region of the MiSTR tissue where the GSK3i level is intermediate.

Using this reasoning and appropriate marker genes for each time point, cells were categorized into three distinct groups for each time point and condition (ventralized/non-ventralized). These groups were assumed to correspond to cells originating from locations A-B, C, and D-E along the GSK3i gradient, with GSK3i levels of 0.4, 1.0, and 1.6, respectively (see Fig. 1D). Since the MiSTR tissue was spatially uniform at day 0, as the cells were initially human embryonic stem cells (hESCs), it was not possible to group the day 0 data into separate spatial locations.

The marker genes used for the assignment of location cell groups were as follows.

- For day 1 and 2:

- Rostral markers: OTX2, FEZF1, SIX3
- Caudal markers: GBX2, HOXA1, HOXB1

- For day 5 and 9:

- Forebrain markers: OTX2, FEZF1, SIX3, LHX2
- Midbrain markers: OTX2, EN1, PAX2
- Hindbrain markers: GBX2, IRX3, HOXA2, HOXA3

- For day 14:

- Forebrain markers: FOXG1, LHX2, TBR1, SIX3, FEZF1, OTX2
- Midbrain markers: EN1, EN2, WNT1, LMX1A, PAX5, PAX8
- Hindbrain markers: GBX2, IRX3, HOXA2, HOXA3, HOXA5

Following the spatial assignment of cells, gene expression levels were averaged for each location group, separately for each gene associated with rostral-caudal and dorsal-ventral patterning. The resulting averaged values were interpreted as the mean gene expression levels for the respective spatial locations. These mean expression levels were subsequently used to generate normalized time series in Python 3 using the NumPy library^39^, following the same methodology applied to the bulk data, as previously described.

#### Hierarchical clustering of scRNA-seq data sets

For both the rostrocaudal (R-C) and dorsoventral (D-V) gene datasets, hierarchical clustering was conducted using the normalized covariance matrix C, defined as the matrix of standard Pearson correlation coefficients derived from the respective datasets. Each element C_ij_ of this matrix represents the Pearson correlation coefficient between the expression profiles of genes i and j.

For the R-C genes, only the non-ventralized dataset was relevant. Therefore, C_ij_ was computed by considering gene expression levels across all time points (days 0, 1, 2, 5, 9, and 14) and spatial locations (A-B, C, and D-E) for each gene pair. In contrast, for the D-V genes, both ventralized and non-ventralized datasets were relevant, and C_ij_ was calculated by incorporating expression levels from all time points and locations across both datasets.

Hierarchical clustering was implemented in Python using the clustermap function from the Seaborn library^43^.

### Deterministic simulations of gene regulation via the Shea-Ackers formalism

This section gives a justification of the form of our GRN model equations. Given a GRN and expression levels $[{\rm{G}}]({t}_{0})=[{{\rm{G}}}_{0}]$ of its N constituent genes $G=({g}_{1},{g}_{2},...,{g}_{N})$ for some initial time ${t}_{0}$, we are interested in simulating $[{\rm{G}}](t)$ for times $t > {t}_{0}$. With the assumption of continuous time and levels, this problem is suitably viewed as that of solving a set of ordinary differential equations (ODEs).

1 $$\frac{d[{\rm{G}}](t)}{{dt}}=f\left(\left[{\rm{G}}\right]\left(t\right)\right)\,{\rm{subject}}\,{\rm{to}}\,[{\rm{G}}]({t}_{0})=[{\rm{G}}{]}_{0}$$

where f is the function of the current GRN expression levels, [G](t). We desire f to be an accurate model of the GRN dynamics. To this end, we assume that mRNA is produced via transcription, and that mRNA levels degrade at a rate proportional to $[{\rm{G}}]$. Protein levels are assumed to follow the corresponding mRNA levels. This allows us to write f as the difference between an mRNA production term P and an mRNA degradation term. For a given gene ${{\rm{g}}}_{i}$ in the GRN, f can then be written as:

2 $$f([{{\rm{g}}}_{i}])={P}_{i}-{\delta }_{i}[{{\rm{g}}}_{i}],$$

where ${\delta }_{i}$ is the exponential decay/degradation constant, or degradation rate, of mRNA i. The degradation rate relates to the mRNA half-life ${t}_{1/2}$ via the relationship ${t}_{1/2}=\frac{\mathrm{ln}2}{\delta }$.

The form of P should take into account that mRNA is produced faster / slower if a gene is activated/repressed by transcription factors (TFs). An intuitive scheme for writing down a production rate for this situation is the Shea-Ackers (S-A) formalism, where gene regulation is viewed through a statistical mechanical lens^44^. The S-A production rate for a specific gene ${{\rm{g}}}_{i}$ in a GRN is written as a fraction, which in a partition function-like manner, yields the transcription rate from the probability of the gene being in a state of active transcription. For a single gene ${g}_{i}$ which is being regulated by ${N}_{A}$ and ${N}_{R}$ *independently* acting activator TFs A and repressor TFs R, respectively, ${P}_{i,{\rm{S}}-{\rm{A}}}$ is obtained as in Eq. 3 below, where ${n}_{j}$ and ${n}_{k}$ are cooperativity parameters for activator j and repressor k, respectively. If n>1, this is interpreted as that multiple TFs of the same kind must jointly bind to DNA to influence the rate of transcription. ${c}_{j}$ and $c{{\prime} }_{k}$ are parameters which describe the strength of a given regulatory connection, and p is the maximum rate of transcription at full activation / no repression.

3 $${P}_{i,{\rm{S}}-{\rm{A}}}=p\cdot \frac{\mathop{\sum }\nolimits_{j=1}^{{N}_{A}}{c}_{j}{[{{\rm{A}}}_{j}]}^{{n}_{j}}}{1+\mathop{\sum }\nolimits_{j=1}^{{N}_{A}}{c}_{j}{[{{\rm{A}}}_{j}]}^{{n}_{j}}+\mathop{\sum }\nolimits_{k=1}^{{N}_{R}}{c}_{k}^{{\prime} }{[{{\rm{R}}}_{k}]}^{{n}_{k}^{{\prime} }}}$$

If transcription factors (TFs) do not act independently, the functional form of P_i,S−A_ will deviate from that described in Eq. 3^45^. However, in the absence of data specifying the regulatory logic of the processes we modeled, we assumed independent TF action as a reasonable initial approximation.

Following the approach outlined by Merlevede et al. ^46^, we slightly modified Eq. 3 to explicitly incorporate a time-independent background regulation of gene expression by genes not included in our model gene regulatory networks (GRNs). This modification assumes that some activator and repressor terms in Eq. 3 remain constant over time. Consequently, their contributions can be aggregated into basal activation and basal repression terms, denoted as a and r, respectively. With this adjustment, Eq. 3 can be reformulated into its final version, which we employ to define our models:

4 $${P}_{i,{\rm{S}}-{\rm{A}}}=p\cdot \frac{a+\mathop{\sum }\nolimits_{j=1}^{{N}_{A}}{c}_{j}{[{{\rm{A}}}_{j}]}^{{n}_{j}}}{1+a+r+\mathop{\sum }\nolimits_{j=1}^{{N}_{A}}{c}_{j}{[{{\rm{A}}}_{j}]}^{{n}_{j}}+\mathop{\sum }\nolimits_{k=1}^{{N}_{R}}{c}_{k}^{{\prime} }{[{{\rm{R}}}_{k}]}^{{n}_{k}^{{\prime} }}}.$$

#### The tri-stable rostral-caudal model equations

The rostral-caudal gene regulatory network (GRN) model depicted in Fig. 3A was adapted from Brambach et al. ^12^. However, the original equations presented in ref.^12^ were modified to align with our generalized Shea-Ackers (S-A) simulation framework. By applying Eq. 4 to the tri-stable rostral-caudal GRN, we derived the following S-A equations to describe its production and degradation dynamics.

5 $$\begin{array}{llll}\frac{d[{\rm{FB}}]}{{dt}}&=&{p}_{1}\cdot \frac{{a}_{1}+{c}_{1}{[{\rm{GSK}}3]}^{{n}_{1}}+{c}_{2}{[{\rm{FB}}]}^{{n}_{2}}}{1+{a}_{1}+{r}_{1}+{c}_{1}{[{\rm{GSK}}3]}^{{n}_{1}}+{c}_{2}{[{\rm{FB}}]}^{{n}_{2}}+{c}_{3}{[{\rm{MB}}]}^{{n}_{3}}+{c}_{4}{[{\rm{HB}}]}^{{n}_{4}}}-{\delta }_{1}[{\rm{FB}}],\\ \frac{d[{\rm{MB}}]}{{dt}}&=&{p}_{2}\cdot \frac{{a}_{2}+{c}_{6}{[{\rm{MB}}]}^{{n}_{6}}}{1+{a}_{2}+{r}_{2}+{c}_{5}{[{\rm{GSK}}3]}^{{n}_{5}}+{c}_{6}{[{\rm{MB}}]}^{{n}_{6}}+{c}_{7}{[{\rm{FB}}]}^{{n}_{7}}+{c}_{8}{[{\rm{HB}}]}^{{n}_{8}}}-{\delta }_{2}[{\rm{MB}}],\\ \frac{d[{\rm{HB}}]}{{dt}}&=&{p}_{3}\cdot \frac{{a}_{3}+{c}_{10}{[{\rm{HB}}]}^{{n}_{10}}}{1+{a}_{3}+{r}_{3}+{c}_{9}{[{\rm{GSK}}3]}^{{n}_{9}}+{c}_{10}{[{\rm{HB}}]}^{{n}_{10}}+{c}_{11}{[{\rm{FB}}]}^{{n}_{11}}+{c}_{12}{[{\rm{MB}}]}^{{n}_{12}}}-{\delta }_{3}[{\rm{HB}}],\\ \frac{d[{\rm{GSK}}3]}{{dt}}&=&{p}_{4}\cdot \frac{{a}_{4}+{c}_{13}{[{\rm{GSK}}3]}^{{n}_{13}}}{1+{a}_{4}+{r}_{4}+{c}_{13}{[{\rm{GSK}}3]}^{{n}_{13}}+{c}_{14}{[{\rm{GSK}}3{\rm{i}}]}^{{n}_{14}}}-{\delta }_{4}[{\rm{GSK}}3],\\ \frac{d[{\rm{GSK}}3{\rm{i}}]}{{dt}}&=&0.\end{array}$$

#### The expanded rostral-caudal model equations

The expanded rostral-caudal GRN in Fig. 4A was obtained by combining the tri-stable rostral-caudal model in Fig. 3A with a modified version of the GRN in ref.^22^, where the latter describes isthmic organizer formation due to how OTX2 and GBX2 respond to WNT (GSK3i) signaling. The following S-A equations were used during the optimization and data comparison stage for this expanded GRN model:

6 $$\begin{array}{llll}\frac{d[{\rm{OTX}}2]}{{dt}}&=&{p}_{1}\cdot \frac{{a}_{1}}{1+{a}_{1}+{r}_{1}+{c}_{1}{[{\rm{GSK}}3{\rm{i}}]}^{{n}_{1}}+{c}_{2}{[{\rm{GBX}}2]}^{{n}_{2}}}-{\delta }_{1}[{\rm{OTX}}2],\\ \frac{d[{\rm{GBX}}2]}{{dt}}&=&{p}_{2}\cdot \frac{{a}_{2}+{c}_{3}{[{\rm{GSK}}3{\rm{i}}]}^{{n}_{3}}}{1+{a}_{2}+{r}_{2}+{c}_{3}{[{\rm{GSK}}3{\rm{i}}]}^{{n}_{3}}+{c}_{4}{[{\rm{OTX}}2]}^{{n}_{4}}}-{\delta }_{2}[{\rm{GBX}}2],\\ \frac{d[{\rm{WNT}}1]}{{dt}}&=&{p}_{3}\cdot \frac{{a}_{3}+{c}_{5}{[{\rm{OTX}}2]}^{{n}_{5}}+{c}_{6}{[{\rm{FGF}}8]}^{{n}_{6}}}{1+{a}_{3}+{r}_{3}+{c}_{5}{[{\rm{OTX}}2]}^{{n}_{5}}+{c}_{6}{[{\rm{FGF}}8]}^{{n}_{6}}+{c}_{7}{[{\rm{GBX}}2]}^{{n}_{7}}}-{\delta }_{3}[{\rm{WNT}}1],\\ \frac{d[{\rm{FGF}}8]}{{dt}}&=&{p}_{4}\cdot \frac{{a}_{4}+{c}_{8}{[{\rm{GBX}}2]}^{{n}_{8}}+{c}_{9}{[{\rm{WNT}}1]}^{{n}_{9}}+{c}_{10}{[{\rm{MB}}]}^{{n}_{10}}}{1+{a}_{4}+{r}_{4}+{c}_{8}{[{\rm{GBX}}2]}^{{n}_{8}}+{c}_{9}{[{\rm{WNT}}1]}^{{n}_{9}}+{c}_{10}{[{\rm{MB}}]}^{{n}_{10}}+{c}_{11}{[{\rm{OTX}}2]}^{{n}_{11}}}-{\delta }_{4}[{\rm{FGF}}8],\\ \frac{d[{\rm{MB}}]}{{dt}}&=&{p}_{5}\cdot \frac{{a}_{5}+{c}_{12}{[{\rm{WNT}}1]}^{{n}_{12}}+{c}_{13}{[{\rm{FGF}}8]}^{{n}_{13}}}{1+{a}_{5}+{r}_{5}+{c}_{12}{[{\rm{WNT}}1]}^{{n}_{12}}+{c}_{13}{[{\rm{FGF}}8]}^{{n}_{13}}+{c}_{14}{[{\rm{FB}}]}^{{n}_{14}}+{c}_{15}{[{\rm{HB}}]}^{{n}_{15}}}-{\delta }_{5}[{\rm{MB}}],\\ \frac{d[{\rm{FB}}]}{{dt}}&=&{p}_{6}\cdot \frac{{a}_{6}+{c}_{16}{[{\rm{OTX}}2]}^{{n}_{16}}}{1+{a}_{6}+{r}_{6}+{c}_{16}{[{\rm{OTX}}2]}^{{n}_{16}}+{c}_{17}{[{\rm{MB}}]}^{{n}_{17}}+{c}_{18}{[{\rm{HB}}]}^{{n}_{18}}}-{\delta }_{6}[{\rm{FB}}],\\ \frac{d[{\rm{HB}}]}{{dt}}&=&{p}_{7}\cdot \frac{{a}_{7}+{c}_{19}{[{\rm{GBX}}2]}^{{n}_{19}}}{1+{a}_{7}+{r}_{7}+{c}_{19}{[{\rm{GBX}}2]}^{{n}_{19}}+{c}_{20}{[{\rm{MB}}]}^{{n}_{20}}+{c}_{21}{[{\rm{FB}}]}^{{n}_{21}}}-{\delta }_{7}[{\rm{HB}}],\\ \frac{d[{\rm{GSK}}3{\rm{i}}]}{{dt}}&=&0.\end{array}$$

Equation 6 corresponds to the GRN with green arrows in Fig. 4A, where MB activation by WNT1 and FGF8 morphogens has been replaced by the *direct* activations of MB via the WNT1 and FGF8 genes. This simplification of the GRN, where the WNT and FGF morphogen nodes are omitted, was necessary to make model optimization tractable: it is not feasible to solve the diffusion equations which govern the spread of the WNT1 and FGF8 morphogens during optimization.

#### The dorsal-ventral model equations

The foundational dorsal-ventral gene regulatory network (GRN) model topology, illustrated on the far left in Fig. 5A, was adapted from Balaskas et al. ^13^. Rather than employing the original equations from Balaskas et al.^13^, we derived the model equations using our generalized framework, as described in Eq. 4. The fully refined dorsal-ventral GRN topology, which incorporates self-activations and GLI-mediated repression (Fig. 5A), is governed by the following Shea-Ackers (S-A) equations, where D, L, and V denote the dorsal, lateral, and ventral nodes, respectively.

7 $$\begin{array}{llll}\frac{d[{\rm{D}}]}{{dt}}&=&{p}_{1}\cdot \frac{{a}_{1}+{c}_{10}{[{\rm{D}}]}^{{n}_{10}}}{1+{a}_{1}+{r}_{1}+{c}_{1}{[{\rm{V}}]}^{{n}_{1}}+{c}_{2}{[{\rm{L}}]}^{{n}_{2}}+{c}_{10}{[{\rm{D}}]}^{{n}_{10}}}-{\delta }_{1}[{\rm{D}}],\\ \frac{d[{\rm{L}}]}{{dt}}&=&{p}_{2}\cdot \frac{{a}_{2}+{c}_{11}{[{\rm{L}}]}^{{n}_{11}}+{c}_{3}{[{\rm{GLI}}]}^{{n}_{3}}}{1+{a}_{2}+{r}_{2}+{c}_{11}{[{\rm{L}}]}^{{n}_{11}}+{c}_{13}{[{\rm{GLI}}]}^{{n}_{13}}+{c}_{3}{[{\rm{GLI}}]}^{{n}_{3}}+{c}_{4}{[{\rm{V}}]}^{{n}_{4}}}-{\delta }_{2}[{\rm{L}}],\\ \frac{d[{\rm{V}}]}{{dt}}&=&{p}_{3}\cdot \frac{{a}_{3}+{c}_{12}{[{\rm{V}}]}^{{n}_{12}}+{c}_{5}{[{\rm{GLI}}]}^{{n}_{5}}}{1+{a}_{3}+{r}_{3}+{c}_{12}{[{\rm{V}}]}^{{n}_{12}}+{c}_{14}{[{\rm{GLI}}]}^{{n}_{14}}+{c}_{5}{[{\rm{GLI}}]}^{{n}_{5}}+{c}_{6}{[{\rm{L}}]}^{{n}_{6}}+{c}_{7}{[{\rm{D}}]}^{{n}_{7}}}-{\delta }_{3}[{\rm{V}}],\\ \frac{d[{\rm{GLI}}]}{{dt}}&=&{p}_{4}\cdot \frac{{a}_{4}+{c}_{8}{[{\rm{SHH}}]}^{{n}_{8}}}{1+{a}_{4}+{r}_{4}+{c}_{8}{[{\rm{SHH}}]}^{{n}_{8}}+{c}_{9}{[{\rm{GSK}}3{\rm{i}}]}^{{n}_{9}}}-{\delta }_{4}[{\rm{GLI}}],\\ \frac{d[{\rm{GSK}}3{\rm{i}}]}{{dt}}&=&0,\\ \frac{d[{\rm{SHH}}]}{{dt}}&=&0.\end{array}$$

The topology without self-interactions is defined by removing the terms that contain parameters number 10–12 from Eq. 7, and the topology without GLI repressions is similarly defined by removing terms containing parameter numbers 13–14 from the same equation. The “basic” unmodified literature topology is defined by removing both of these groups of terms.

### Optimization of models against experimental data

Below, we outline our general Python-based workflow for optimizing gene regulatory network (GRN) models against experimental data. The optimization procedure is graphically summarized in the flow chart presented in Fig. S2A.

Our objective was to align the dynamic behavior of a given GRN model as closely as possible with single-cell RNA sequencing (scRNA-seq) data for the genes included in the GRN. This involved numerically solving the Shea-Ackers ordinary differential equations (ODEs) for the GRN, using day 0 scRNA-seq data as initial conditions. We then compared the simulated gene expression levels with the scRNA-seq data at days 1, 2, 5, 9, and 14 of the simulation timeline. Since gene expression levels were available for three spatial locations: A-B, C, and D-E, each characterized by distinct levels of GSK3i (see Fig. 1D), our comparison incorporated data from all three conditions. The optimization process was framed as a search for an optimal set of model parameters, ppp, that maximized the agreement between simulations and experimental data. This was accomplished by minimizing a cost function, C(p).

8 $${\displaystyle{C}{\bf{p}}}=\mathop{\sum}\limits_{g}\mathop{\sum}\limits_{l}\mathop{\sum }\limits_{t}{\left({s}_{{gl}}({\bf{p}},t)-{d}_{{gl}}(t)\right)}^{2}+\mathop{\sum}\limits_{g}\mathop{\sum}\limits_{l}\alpha {\left({\frac{|{{s}_{{gl}}}({\bf{p}},t)}{\partial t}|_{t={\mathrm{day}}14}}\right)}^{2}$$

The cost function, C(p), comprises two distinct types of terms. The first set of terms consists of standard least-squares components, designed to minimize the differences between simulated gene expression levels, s_gl_(p,t), and experimental data, d_gl_(t), for each gene ggg in the gene regulatory network (GRN) model. The summations over t and l span all time points t ≠ day 0 for which single-cell RNA sequencing (scRNA-seq) data are available, as well as all experimental conditions, i.e., spatial locations l (or equivalently, average GSK3i levels at each location). The second set of terms enforces steady-state constraints, ensuring that physically unrealistic simulation outcomes are excluded from consideration as optimal solutions. The hyperparameter α, which balances these terms, was manually tuned to *α* ≈ 0.0392.

Optimization of the GRN models against experimental data was conducted in a two-step process. Initially, fuzzy self-tuning particle swarm optimization (FST-PSO), which is a global optimization algorithm^47^, was employed to broadly explore the parameter space and identify promising local minima in the cost function C(p). Subsequently, the limited memory Broyden-Fletcher-Goldfarb-Shanno box-constrained (L-BFGS-B)^48^, which is a local optimization algorithm, was utilized to refine the parameters corresponding to the most promising minimum identified by FST-PSO, using the FST-PSO results as initial conditions.

During the optimization process, ordinary differential equations (ODEs) were numerically integrated over a total time of 336 h, corresponding to the full 14-day duration of the MiSTR experiment. The ODEs were solved using the “odeint” routine from the SciPy library^49^, selected for its efficiency and automatic detection of ODE stiffness, which allows it to switch between solvers as needed. Additionally, the Python library Numba^50^ was employed to accelerate the ODE solving process by approximately an order of magnitude. The integration time step was set to 1/5000 of the total integration time, i.e., 0.0028 h. The upper and lower bounds for parameter searches in parameter space were determined empirically and are specified as follows for each of the six parameter types (expressed as [lower bound, upper bound]):

$$\begin{array}{c}c:\left[{10}^{-4},{10}^{4}\right]\,n:\left[1,4\right]\,a:\left[{10}^{-4},{10}^{1}\right]\,r:\left[{10}^{-4},{10}^{1}\right]\\ p:\left[{10}^{-4},{10}^{0}\right]\,\delta :[{10}^{-4},{10}^{0}]\end{array}$$

Other values than those above were used for two of the dorsal-ventral GRN topologies; see below. Parameter values were uniformly sampled in log_10_-spaces made up of the upper and lower bounds to ensure an even coverage of the parameter space. FST-PSO was run using a population size of 150 particles and was run for 200 iterations in a given optimization run. Finally, L-BFGS-B was run with a budget of ${10}^{5}$ evaluations of $C({\bf{p}})$.

It should be emphasized that GSK3i and SHH levels were only present during the first 9 days of simulation time during optimization: these signals were always turned off (i.e., put = 0) after day 9 (at *t* = 216 h) such that optimization was performed under conditions which replicated the experimental MiSTR conditions as closely as possible.

#### Optimizing the tri-stable rostral-caudal model

All 420 combinations of forebrain (FB), midbrain (MB), and hindbrain (HB) genes depicted in Fig. 2A were evaluated during the optimization of this model. A total of 15 optimization runs were conducted for each combination, with the resulting cost function values presented in Fig. S3A-F. Since gene expression data for GSK3 were unavailable, the initial condition was set to [GSK3] = 1, consistent with the initial condition used in Brambach et al. ^12^.

The optimal parameter set, utilized for the simulation shown in Fig. 3B, was selected based on two criteria: achieving a low cost function value and producing a continuous gene expression pattern, as illustrated in Fig. 3C. This pattern distinctly exhibited three regions of gene expression: FB dominance at low GSK3i levels, MB at intermediate levels, and HB at high GSK3i levels.

#### Optimizing the expanded rostral-caudal model

The midbrain (MB) gene regulatory network (GRN) node was constrained to represent the gene EN1 during optimization, as Engrailed (EN) genes are integral to defining the MB region within the GRN framework^22^, which informed the isthmic organizer component of our model. EN1 is widely recognized as a reliable marker of MB cell types^51^.

Given the critical role of the OTX2-GBX2 response to GSK3i in positioning the isthmic organizer, the parameters associated with this segment of the GRN were optimized independently prior to the optimization of the remaining GRN parameters. This sequential approach was feasible due to the absence of feedback loops from other GRN nodes to OTX2 or GBX2, effectively decoupling this sub-network from the rest of the GRN and simplifying the overall optimization problem.

With MB = EN1, we conducted 100 optimization runs for each of the 30 combinations of forebrain (FB) and hindbrain (HB) genes from Fig. 2A. The cost function values for these runs are displayed in Figure S4A. Genes that already served specific roles as other nodes in the GRN (e.g., OTX2 and GBX2) were excluded from consideration during optimization. For this GRN, the optimal parameter set, illustrated in Fig. 4C, was primarily selected based on its ability to produce a continuous gene expression pattern that closely matched the schematic representation in Fig. 4B. The cost function value was a secondary consideration. Particular emphasis was placed on ensuring that the continuous pattern along the GSK3i gradient (Fig. 4D) at day 9 remained essentially unchanged at day 14, five days after the removal of the GSK3i gradient. For reference, a simulation using the parameter set that yielded the lowest cost function value, but did not produce a pattern in as good agreement with Fig. 4B, is shown in Fig. S4B.

#### Optimizing the dorsal-ventral models

The dorsal-ventral models, as defined in Eq. 7, were systematically optimized against experimental data under both control (day 0–9: SHH = 0) and ventralized (day 0–9: SHH = 1) conditions. Parameters associated with the GLI gene regulatory network (GRN) node response to Sonic Hedgehog (SHH) and Wingless/Integrated (WNT) signaling were optimized independently prior to the optimization of the dorsal, ventral, and lateral nodes. Optimization targeting GLI1 yielded satisfactory results, whereas optimization targeting GLI2 or GLI3 did not. This discrepancy likely arises from the observation in the MiSTR dataset that only GLI1, and not GLI2 or GLI3, exhibits high expression in the presence of SHH and low expression in its absence, a finding consistent with earlier studies^52^. Consequently, the optimal parameter set derived from GLI1 response optimization was consistently employed during the optimization of the dorsal, ventral, and lateral nodes.

For each of the four model topologies, 100 optimization runs were conducted. The optimal parameter sets, selected to generate the plots in Fig. 5B, were chosen based on two primary criteria. First, the parameter sets were required to demonstrate strong agreement with experimental data, as evidenced by low cost function values. Second, the simulated day 14 pattern in (SHH, WNT)-space was required to be qualitatively accurate, exhibiting at least dorsally and ventrally dominant regions, and preferably also a laterally dominant region at intermediate day 0–9 WNT/SHH values. This latter criterion was only satisfied by the doubly modified topology, for which the 20 best parameter sets and their corresponding cost function values are presented in Fig. S5.

To prevent parameter values from being constrained by the predefined bounds, some of the standard parameter bounds were adjusted. Specifically, for the literature topology and the topology incorporating repressive GLI, the following revised parameter bounds were applied:

$$\begin{array}{c}c:\left[{10}^{-4},{10}^{4}\right]\,n:\left[1,4\right]\,a:\left[{10}^{-6},{10}^{0}\right]\,r:\left[{10}^{-5},{10}^{1}\right]\\ p:\left[{10}^{-5},{10}^{1}\right]\,\delta :[{10}^{-4},{10}^{0}\end{array}$$

### Model sensitivity analysis

A sensitivity analysis was conducted for the optimal parameter set of each gene regulatory network (GRN) model. For each model, the analysis was performed as follows: For every parameter p_i_ in the system of ordinary differential equations (ODEs) defining the GRN, the parameter value was independently perturbed by +10% and -10%. While maintaining all other model parameters at their baseline values, the model dynamics were simulated to evaluate the cost function C(**p**) for these two modified parameter sets. The average relative change in C(**p**), compared to its baseline value, was subsequently computed.

9 $${\rm{Relative\; change\; in\; cost}}=\frac{C({\bf{p}},{p}_{i}=+10 \% )+C({\bf{p}},{p}_{i}=-10 \% )}{2C({\bf{p}})}$$

and is a measure of how sensitive the model is to changes in ${p}_{i}$. The results of the sensitivity analyses are shown in Figure S6.

### Simulations of patterning in 3D geometry with optimized models

The goal of the spatial 3D simulations was to find the time-evolution of patterning GRN model genes in a 3D voxel geometry which represented the early in vivo neural tube, given one or several morphogen distributions $[{\rm{M}}](x,y,z)$ throughout this geometry. The voxel geometry and morphogen secretion regions Fig. 6A, B were obtained from^12^. Our general 3D simulation framework is described below.

#### The general 3D modeling framework

To simulate patterning within a given geometry, we begin with a steady-state morphogen gradient [M](x,y,z), where the time evolution of the patterning gene regulatory network (GRN) is computed for each voxel in the geometry using the local [M] as input. However, if morphogens are dynamically produced from specific source locations, then the morphogen concentration [M](x,y,z) becomes time-dependent. Additionally, if morphogens can diffuse between voxels, then [M](x,y,z,t) evolves in a manner that is both complex and specific to the geometry. We assume that morphogen transport occurs via passive diffusion. Since diffusion between voxels is constrained to directions depending on the presence of neighboring voxels, it is not uniformly possible in all directions. Given the intricate structure of the neural tube geometry, tracking how possible diffusion directions vary between voxels presents a significant challenge. Nevertheless, accurately modeling these directional dependencies is crucial for simulating the temporal evolution of [M](x,y,z,t).

To address the challenges arising from the approach above, we reformulated the 3D morphogen diffusion problem into a 1D diffusion problem. This transformation allowed us to encode all information regarding each voxel’s neighboring voxels into a matrix A, which we then used to determine the possible diffusion directions for each voxel. To express the diffusion problem in terms of this “neighbor matrix,” we mapped each voxel’s (x,y,z) coordinate to a unique site number s_i_, serving as a distinct identifier for the voxel’s position. The number s_i_ thus corresponds to the i-th site in the site space s. Given such a mapping (x,y,z)↦s for the entire 3D geometry, the A-matrix is defined as follows:

- diagonal elements ${A}_{{s}_{i}{s}_{i}}$, equal to minus the number of nearest neighbors of ${s}_{i}$ in (x,y,z)-space,
- off-diagonal elements ${A}_{{s}_{i}{s}_{j}}$, i≠j, equal to one if ${s}_{j}$ neighbors ${s}_{i}$ in (x,y,z)-space, zero otherwise.

The diagonal terms of the A-matrix specify the number of adjacent sites from which diffusion occurs, while the off-diagonal terms indicate the sites to which diffusion can take place. This formulation inherently ensures that morphogens do not diffuse outside the simulation geometry, as no-flux (Neumann) boundary conditions are automatically enforced at the geometry edges. To illustrate the s-labeling scheme and the structure of the A-matrix, Fig. S2C presents a simplified example of a 3D geometry, including its (x,y,z) coordinates, the mapped s-values (in purple), and the corresponding A-matrix.

The advantage of formulating the diffusion problem using the A-matrix becomes evident when considering its application. By employing an explicit Euler method, the discretized 3D diffusion equation—governing the forward evolution of morphogen levels [M] over discrete time steps n and spatial coordinates x,y,z—can be expressed as follows:

10 $$[{\rm{M}}{]}_{x,y,z}^{(n+1)}=[{\rm{M}}{]}_{x,y,z}^{(n)}+{D}_{{\rm{M}}}\cdot \varDelta t\frac{[{\rm{M}}{]}_{x+1,y,z}^{(n)}+[{\rm{M}}{]}_{x-1,y,z}^{(n)}+[{\rm{M}}{]}_{x,y+1,z}^{(n)}+[{\rm{M}}{]}_{x,y-1,z}^{(n)}+[{\rm{M}}{]}_{x,y,z+1}^{(n)}+[{\rm{M}}{]}_{x,y,z-1}^{(n)}-6[{\rm{M}}{]}_{x,y,z}^{(n)}}{{a}^{2}}$$

where a is the voxel spacing, Δt is the time step, and ${D}_{{\rm{M}}}$ is the diffusion constant of the morphogen. The constant 6 describes the number of nearest neighbors that M can diffuse to in 3D. However, this constant is <6 for the sites where diffusion is not possible in all directions. In this case, terms in the numerator of Eq. 10 corresponding to diffusion from non-existing sites should be omitted, and so we have a cumbersome, site-dependent diffusion equation to solve. Our ${\rm{A}}$-matrix formulation makes this problem less cumbersome, as it allows us to write Eq. 10 as a matrix equation. With the notation $[{\rm{M}}]$ for a vector which contains the morphogen levels in s-space for all voxel sites, and ${\rm{I}}$ for the identity matrix, we can write the diffusion equation in terms of ${\rm{A}}$ as:

11 $${[{\rm{M}}]}^{(n+1)}=\left({\rm{I}}+\frac{{D}_{M}\cdot \varDelta t}{{a}^{2}}{\rm{A}}\right){[{\rm{M}}]}^{(n)}$$

Equipped with the ${\bf{A}}$-matrix for our 3D geometry, the relevant morphogen and GRN equations can now be solved for all voxel sites via Eq. 11.

All 3D simulations were performed in Python, making use of the NumPy^35^ and Numba^50^ libraries to improve computational efficiency. As in ref.^12^, we used a voxel spacing of $a=20\mu {\rm{m}}$. Since the explicit Euler formulation of the diffusion equation is only conditionally stable^53^, a sufficiently small Δt had to be chosen when simulating morphogen diffusion. Thus, the time step Δt depended on if both morphogens levels and GRN levels were updated, or if only GRN levels were updated. In the former case, Δt=0.0001 h was used; in the latter case, Δt=0.01 h was deemed sufficient. The diffusion constant value depended on which morphogen was simulated; see the two sections below. The total integration time was always 336 h (14 days), like in the MiSTR experiments. Figure S2B outlines the full patterning simulation scheme in a flow chart.

Our simulations of neural tube patterning involved three distinct classes of morphogens. We have the early rostral-caudal WNT gradient shown in Fig. 6C, the dorsal-ventral gradients of WNT and SHH from the secretion regions in Fig. 6B, and the WNT/FGF morphogens secreted from the isthmic organizer. Morphogen production and degradation were modelled differently depending on which of these morphogen classes were used during the simulations, as is described in the two sections below.

#### Simulations with the expanded rostral-caudal patterning model

The early rostral-caudal WNT signaling gradient shown in Fig. 6C was used as a caudalizing patterning gradient during the first 196 h (9 days) of simulation time; after this, the gradient was removed like in the MiSTR experiments. The gradient was established via diffusion from a group of WNT secretion voxels situated at the caudal-most end of the tube geometry. In these voxels, WNT was assumed to be produced at a constant rate ${p}_{{\rm{WN}}{{\rm{T}}}_{{\rm{early}}}}$. WNT was everywhere assumed to degrade at a rate proportional to the current WNT level, with degradation constant ${\delta }_{{\rm{WN}}{{\rm{T}}}_{{\rm{early}}}}$. Hence, the geometry-wide time-evolution of early rostral-caudal WNT levels, $[{{\rm{WNT}}}_{{\rm{early}}}]$, can be described as follows when both production, degradation, and diffusion is considered:

12 $${[{{\rm{WNT}}}_{{\rm{early}}}]}^{\left(n+1\right)}=\left\{\begin{array}{c}\begin{array}{cc}{p}_{{\rm{WN}}{{\rm{T}}}_{{\rm{early}}}}\cdot \varDelta t+\left((1-{\delta }_{{\rm{WN}}{{\rm{T}}}_{{\rm{early}}}}\cdot \varDelta t)\cdot {\rm{I}}+\frac{{D}_{{\rm{WN}}{{\rm{T}}}_{{\rm{early}}}}\cdot \varDelta t}{{a}^{2}}{\rm{A}}\right){[{{\rm{WNT}}}_{{\rm{early}}}]}^{(n)} & {\rm{within\; secretion\; voxels}},\end{array}\\ \begin{array}{cc}\left((1-{\delta }_{{\rm{WN}}{{\rm{T}}}_{{\rm{early}}}}\cdot \varDelta t)\cdot {\rm{I}}+\frac{{D}_{{\rm{WN}}{{\rm{T}}}_{{\rm{early}}}}\cdot \varDelta t}{{a}^{2}}{\bf{A}}\right){[{{\rm{WNT}}}_{{\rm{early}}}]}^{(n)} & {\rm{outside\; secretion\; voxels}}.\end{array}\end{array}\right.$$

Equation 12 was solved for a total integration time of 2 h, at which time a steady state rostral-caudal WNT gradient had been achieved. To obtain the particular steady-state gradient that was employed during the patterning simulations, we used the following parameter values:

$${p}_{{\rm{WN}}{{\rm{T}}}_{{\rm{early}}}}=81{{\rm{h}}}^{-1},{\delta }_{{\rm{WN}}{{\rm{T}}}_{{\rm{early}}}}=43.2{{\rm{h}}}^{-1},{\rm{and}}{D}_{{\rm{WN}}{{\rm{T}}}_{{\rm{early}}}}=5.4\cdot {10}^{5}{\mu {\rm{m}}}^{2}{{\rm{h}}}^{-1}$$

For generating the early rostral-caudal WNT gradient, diffusion was employed as a computational approach to efficiently establish a smooth gradient within the irregular tube geometry. It is important to note that this method is used for modeling purposes and does not imply that the initial in vivo WNT gradient is formed through a similar diffusion-based mechanism.

The full GRN with isthmic organizer morphogens ${\rm{WN}}{{\rm{T}}}_{{\rm{IsO}}}$ and ${\rm{FG}}{{\rm{F}}}_{{\rm{IsO}}}$ from Fig. 4A was used during the 3D patterning simulations. Hence, the replacements $[{\rm{WNT}}1]\to [{{\rm{WNT}}}_{{\rm{IsO}}}]$ and $[{\rm{FGF}}8]\to [{{\rm{FGF}}}_{{\rm{IsO}}}]$ were made in the MB-equation in Eq. 6. We assumed that isthmic organizer WNT and FGF morphogens were at all times produced at rates proportional to the simulated WNT1 and FGF8 isthmic organizer gene levels, with rate constants ${p}_{{\rm{WN}}{{\rm{T}}}_{{\rm{IsO}}}}$ and ${p}_{{\rm{FG}}{{\rm{F}}}_{{\rm{IsO}}}}$. This ensured that a substantial production of WNT and FGF morphogens occurred only if their respective genes were highly expressed. Because of this, morphogens would only be produced in substantial amounts within the IsO region of the tube. WNT / FGF was assumed to degrade at rates ${\delta }_{{\rm{WN}}{{\rm{T}}}_{{\rm{IsO}}}}$ and ${\delta }_{{\rm{FG}}{{\rm{F}}}_{{\rm{IsO}}}}$. Hence, the time-evolution of WNT and FGF IsO morphogens can be obtained as:

13 $${[{{\rm{WNT}}}_{{\rm{IsO}}}]}^{(n+1)}={p}_{{\rm{WN}}{{\rm{T}}}_{{\rm{IsO}}}}\cdot \varDelta t\cdot {[{\rm{WNT}}1]}^{(n)}+\left((1-{\delta }_{{\rm{WN}}{{\rm{T}}}_{{\rm{IsO}}}}\cdot \varDelta t)\cdot {\rm{I}}+\frac{{D}_{{\rm{WN}}{{\rm{T}}}_{{\rm{IsO}}}}\cdot \varDelta t}{{a}^{2}}{\bf{A}}\right){[{{\rm{WNT}}}_{{\rm{IsO}}}]}^{(n)}$$

14 $${[{{\rm{FGF}}}_{{\rm{IsO}}}]}^{(n+1)}={p}_{{\rm{FG}}{{\rm{F}}}_{{\rm{IsO}}}}\cdot \varDelta t\cdot {[{\rm{FGF}}8]}^{(n)}+\left((1-{\delta }_{{\rm{FG}}{{\rm{F}}}_{{\rm{IsO}}}}\cdot \varDelta t)\cdot {\rm{I}}+\frac{{D}_{{\rm{FG}}{{\rm{F}}}_{{\rm{IsO}}}}\cdot \varDelta t}{{a}^{2}}{\rm{A}}\right){[{{\rm{FGF}}}_{{\rm{IsO}}}]}^{(n)}$$

where $[{\rm{WNT}}1]$ and $[{\rm{FGF}}8]$ are vectors containing the current levels of the WNT1 and FGF genes. We used the following parameter values to simulate the time-evolution of WNT and FGF IsO morphogens:

$$\begin{array}{lll}{p}_{{\rm{WN}}{{\rm{T}}}_{{\rm{IsO}}}}&=&{p}_{{\rm{FG}}{{\rm{F}}}_{{\rm{IsO}}}}=75.6{{\rm{h}}}^{-1},{\delta }_{{\rm{WN}}{{\rm{T}}}_{{\rm{IsO}}}}={\delta }_{{\rm{FG}}{{\rm{F}}}_{{\rm{IsO}}}}=72{{\rm{h}}}^{-1}\\ {D}_{{\rm{WN}}{{\rm{T}}}_{{\rm{IsO}}}}&=&{D}_{{\rm{FG}}{{\rm{F}}}_{{\rm{IsO}}}}=5.4\cdot {10}^{5}{\mu {\rm{m}}}^{2}{{\rm{h}}}^{-1}\end{array}$$

#### Simulations with the modified dorsal-ventral patterning model

The SHH and WNT morphogen gradients (Fig. 6D), established through diffusion from secretion regions located in the dorsal and ventral domains of the neural tube (Fig. 6B), were assumed to govern dorsal-ventral regionalization during the initial 196 h period. This assumption was applied while simulating the temporal evolution of the modified dorsal-ventral patterning model in three dimensions using Eq. 7.

The establishment of these gradients followed a procedure analogous to that used for the early rostral-caudal WNT gradient. Specifically, the gradients were generated through simulations of WNT and SHH production at constant rates from designated secretion voxels, with degradation occurring ubiquitously in proportion to their respective levels. Diffusion between voxels was modeled using Eq. 11. Consequently, the time evolution of dorsal-ventral SHH and WNT concentrations, denoted as $[{{\rm{SHH}}}_{{\rm{DV}}}]$ and $[{{\rm{WNT}}}_{{\rm{DV}}}]$ is described by:

15 $${[{{\rm{SHH}}}_{{\rm{DV}}}]}^{(n+1)}=\left\{\begin{array}{ll}\begin{array}{cc}{p}_{{\rm{SH}}{{\rm{H}}}_{{\rm{DV}}}}\cdot \varDelta t+\left((1-{\delta }_{{\rm{SH}}{{\rm{H}}}_{{\rm{DV}}}}\cdot \varDelta t)\cdot {\rm{I}}+\frac{{D}_{{\rm{SH}}{{\rm{H}}}_{{\rm{DV}}}}\cdot \varDelta t}{{a}^{2}}{\rm{A}}\right){[{{\rm{SHH}}}_{{\rm{DV}}}]}^{(n)} & {\rm{within}}\;{/rm{secretion}}\;{\rm{voxels}},\end{array}\\\begin{array}{lll}\left((1-{\delta }_{{\rm{SH}}{{\rm{H}}}_{{\rm{DV}}}}\cdot \varDelta t)\cdot {\rm{I}}+\frac{{D}_{{\rm{SH}}{{\rm{H}}}_{{\rm{DV}}}}\cdot \varDelta t}{{a}^{2}}{\rm{A}}\right){[{{\rm{SHH}}}_{{\rm{DV}}}]}^{(n)} & {\rm{outside}}\;{\rm{secretion}}\;{\rm{voxels}}.\end{array}\end{array}\right.$$

16 $${[{{\rm{WNT}}}_{{\rm{DV}}}]}^{(n+1)}=\left\{\begin{array}{l}\begin{array}{cc}{p}_{{\rm{WN}}{{\rm{T}}}_{{\rm{DV}}}}\cdot \varDelta t+\left((1-{\delta }_{{\rm{WN}}{{\rm{T}}}_{{\rm{DV}}}}\cdot \varDelta t)\cdot {\rm{I}}+\frac{{D}_{{\rm{WN}}{{\rm{T}}}_{{\rm{DV}}}}\cdot \varDelta t}{{a}^{2}}{\rm{A}}\right){[{{\rm{WNT}}}_{{\rm{DV}}}]}^{(n)} & {\rm{within\; secretion\; voxels}},\end{array}\\ \begin{array}{cc}\left((1-{\delta }_{{\rm{WN}}{{\rm{T}}}_{{\rm{DV}}}}\cdot \varDelta t)\cdot {\rm{I}}+\frac{{D}_{{\rm{WN}}{{\rm{T}}}_{{\rm{DV}}}}\cdot \varDelta t}{{a}^{2}}{\rm{A}}\right){[{{\rm{WNT}}}_{{\rm{DV}}}]}^{(n)} & {\rm{outside\; secretion\; voxels}}.\end{array}\end{array}\right.$$

Equations 15 and 16 were solved for a total integration time of 2 h, at which time steady state dorsal-ventral SHH and WNT gradients had been achieved. To obtain the steady-state gradients that were employed during the patterning simulations, we used the following parameter values:

$$\begin{array}{l}{p}_{{\rm{SH}}{{\rm{H}}}_{{\rm{DV}}}}={p}_{{\rm{WN}}{{\rm{T}}}_{{\rm{DV}}}}=1800{{\rm{h}}}^{-1},{\delta }_{{\rm{SH}}{{\rm{H}}}_{{\rm{DV}}}}={\delta }_{{\rm{WN}}{{\rm{T}}}_{{\rm{DV}}}}=36{{\rm{h}}}^{-1},{D}_{{\rm{SHH}}{{\rm{b}}}_{{\rm{DV}}}}\\\qquad\quad=4.8024\cdot {10}^{5}{\mu {\rm{m}}}^{2}{{\rm{h}}}^{-1},{\rm{and}}{D}_{{\rm{WN}}{{\rm{T}}}_{{\rm{DV}}}}=5.4242\cdot {10}^{5}{\mu {\rm{m}}}^{2}{{\rm{h}}}^{-1}\end{array}$$

The diffusion constants utilized in this study are derived from Brambach et al. ^12^ and are based on estimates for freely diffusing SHH and WNT molecules. While we recognize that this representation likely simplifies the complex mechanisms underlying SHH and WNT transport, it serves as a reasonable and justified initial assumption for our modeling framework.

When simulating dorsal-ventral patterning in isolation, Eq. 7 was applied with $[{\rm{WNT}}]=[{\rm{WN}}{{\rm{T}}}_{{\rm{DV}}}]$ and $[{\rm{SHH}}]=[{\rm{SH}}{{\rm{H}}}_{{\rm{DV}}}]$. To investigate the influence of WNT morphogen levels originating from the isthmic organizer ($[{\rm{WN}}{{\rm{T}}}_{{\rm{IsO}}}]$) on dorsal-ventral patterning, simulations of both rostral-caudal and dorsal-ventral development were conducted in parallel. In these simulations, the WNT concentration was defined as $[{\rm{WNT}}]=[{\rm{WN}}{{\rm{T}}}_{{\rm{DV}}}]+c\cdot [{\rm{WN}}{{\rm{T}}}_{{\rm{IsO}}}]$ in Eq. 7. The scaling constant c was introduced to account for potential variations in the influence of WNT from different sources on GLI activity. For the simulation results presented in Fig. 6I, J, a value of *c* = 20 was employed.

## Supplementary information

Supplementary information

Neural_tube_project_essentials

## Data Availability

Processed MiSTR single-cell and single-nucleus transcriptomic datasets from Rathore et al. are publicly available through the UCSC Cell Browser at https://cells.ucsc.edu/?ds=neural-tube-organoids^11^. In the present study, this resource is relevant to the MiSTR scRNA-seq data used to generate time-resolved expression inputs for model fitting and comparison, specifically the R/C dorsal and R/C ventral MiSTR datasets from days 0, 1, 2, 5, 9, and 14.

## Code availability

The custom computer code, scripts, and simulation workflows developed and used in this study to model neural tube patterning and isthmic organizer formation are publicly available on GitHub at https://github.com/CodeByAD/Model_Neural_Tube_Patterning_and_Isthmic_Organizer_Formation/tree/main.

## References

[1] Sanes, D. H., T. A. Reh, and W. A. Harris. *Development of the Nervous System*, *4th Edition*. (Academic Press, 2019).

[2] Rogers, K. & Schier, A. Morphogen gradients: from generation to interpretation. *Annu. Rev. Cell Dev. Biol.* **27**, 377–407 (2010). 10.1146/annurev-cellbio-092910-154148 21801015

[3] Briscoe, J., and Thérond, P. The mechanisms of hedgehog signaling and its roles in development and disease. *Nat. Rev. Mol. Cell Biol.* **14**. 10.1038/nrm3598. (2013). 10.1038/nrm3598 23719536

[4] Lopes, F. Spatial bistability generates hunchback expression sharpness in the Drosophila embryo. *PLoS Comput. Biol.* **4**, e1000184 (2008). 18818726 10.1371/journal.pcbi.1000184 PMC2527687

[5] Stapornwongkul, K. & Vincent, J.-P. Generation of extracellular morphogen gradients: the case for diffusion. *Nat. Rev. Genet.* **22**, 1–19, 10.1038/s41576-021-00342-y (2021). 33767424 10.1038/s41576-021-00342-y

[6] Nordström, U. Progressive induction of caudal neural character by graded WNT signaling. *Nat. Neurosci.* **5**, 525–532 (2002). 12006981 10.1038/nn0602-854

[7] Gibbs, H. Midbrain-hindbrain boundary morphogenesis: at the intersection of Wnt and Fgf signaling. *Front. Neuroanat.* **11**, 64 (2017). 28824384 10.3389/fnana.2017.00064 PMC5541008

[8] Yu, S. Fgf8 morphogen gradient forms by a source-sink mechanism with freely diffusing molecules. *Nature* **461**, 533–536 (2009). 19741606 10.1038/nature08391

[9] Ulloa, F. & Martí, E. Wnt won the war: antagonistic role of wnt over shh controls dorso-ventral patterning of the vertebrate neural tube. *Dev. Dyn. Off. Publ. Am. Assoc. Anatomists* **239**, 69–76 (2009). 10.1002/dvdy.22058 19681160

[10] Rifes, P., et al. Modeling neural tube development by differentiation of human embryonic stem cells in a microfluidic WNT gradient. *Nat. Biotechnol.* **38**, 1–9. 10.1038/s41587-020-0525-0. (2020). 10.1038/s41587-020-0525-0 PMC7616963 32451506

[11] Rathore, G. S., et al. Mapping early patterning events in human neural development using an in-vitro microfluidic stem cell model. bioRxiv (2025). 10.1101/2025.05.23.655442.

[12] Brambach, M. Neural tube patterning: from a minimal model for rostrocaudal patterning toward an integrated 3D model. *iScience* **24**, 102559 (2021). 34142058 10.1016/j.isci.2021.102559 PMC8184516

[13] Balaskas, N. Gene regulatory logic for reading the sonic hedgehog signaling gradient in the vertebrate neural tube. *Cell* **148**, 273–284 (2012). 22265416 10.1016/j.cell.2011.10.047 PMC3267043

[14] Brafman, D., and Willert, K. Wnt/ beta-catenin signaling during early vertebrate neural development: Wnt signaling in neural development. *Dev. Neurobiol.* **77**. 10.1002/dneu.22517. (2017). 10.1002/dneu.22517 PMC5819875 28799266

[15] Kirkeby, A. Generation of regionally specified neural progenitors and functional neurons from human embryonic stem cells under defined conditions. *Cell Rep.* **1**, 703–714 (2012). 22813745 10.1016/j.celrep.2012.04.009

[16] Gardner, T., Cantor, C. & Collins, J. Construction of a genetic toggle switch in Escherichia Coli. *Nature* **403**, 339–342 (2000). 10659857 10.1038/35002131

[17] Ghaffarizadeh, A., Flann, N. & Podgorski, G. Multistable switches and their role in cellular differentiation networks. *BMC Bioinforma.* **15**, S7 (2014). 10.1186/1471-2105-15-S7-S7 PMC4110729 25078021

[18] Chickarmane, V. Transcriptional dynamics of the embryonic stem cell switch. *PLoS Comput. Biol.* **2**, e123 (2006). 16978048 10.1371/journal.pcbi.0020123 PMC1570179

[19] Millet, S. et al. A role for Gbx2 in repression of Otx2 and positioning the mid/hindbrain organizer. *Nature* **401**, 161–164 (1999). 10490024 10.1038/43664

[20] Joyner, A. L., Liu, A. & Millet, S. Otx2, Gbx2 and Fgf8 interact to position and maintain a mid-hindbrain organizer. *Curr. Opin. Cell Biol.* **12**, 736–741 (2000). 11063941 10.1016/s0955-0674(00)00161-7

[21] Wulst, W. & Bally-Cuif, L. Neural plate patterning: upstream and downstream of the isthmic organizer. *Nat. Rev. Neurosci.* **2**, 99–108 (2001). 11253000 10.1038/35053516

[22] Hock, S. Sharpening of expression domains induced by transcription and MicroRNA regulation within a spatio-temporal model of mid-hindbrain boundary formation. *BMC Syst. Biol.* **7**, 48 (2013). 23799959 10.1186/1752-0509-7-48 PMC4103684

[23] Inoue, F. Gbx2 directly restricts Otx2 expression to forebrain and midbrain, competing with class III POU factors. *Mol. Cell. Biol.* **32**, 2618–2627 (2012). 22566684 10.1128/MCB.00083-12 PMC3434480

[24] Hidalgo-Sánchez, M., Andreu-Cervera, A., Villa-Carballar, S. & Echevarria, D. An update on the molecular mechanism of the vertebrate isthmic organizer development in the context of the neuromeric model. *Front. Neuroanat.* **16**, 826976 (2022). 35401126 10.3389/fnana.2022.826976 PMC8987131

[25] Martynoga, B., Morrison, H., Price, D. J. & Mason, J. O. Foxg1 is required for specification of ventral telencephalon and region-specific regulation of dorsal telencephalic precursor proliferation and apoptosis. *Dev. Biol.* **283**, 113–127 (2005). 15893304 10.1016/j.ydbio.2005.04.005

[26] Hägglund, A.-C., Dahl, L. & Carlsson, L. Lhx2 is required for patterning and expansion of a distinct progenitor cell population committed to eye development. *PloS One* **6**, e23387 (2011). 21886788 10.1371/journal.pone.0023387 PMC3158764

[27] Montavon, T., and Soshnikova, N. Hox gene regulation and timing in embryogenesis. *Semin. Cell Dev. Biol.* **34**. 10.1016/j.semcdb.2014.06.005. (2014). 10.1016/j.semcdb.2014.06.005 24930771

[28] Li, B., Sei Kuriyama, M. M. & Mayor, R. The posteriorizing gene Gbx2 is a direct target of Wnt signalling and the earliest factor in neural crest induction. *Development* **136**, 3267–3278, 10.1242/dev.036954 (2009). 19736322 10.1242/dev.036954 PMC2808295

[29] Elkouby, Y. Mesodermal Wnt signaling organizes the neural plate via Meis3. *Development* **137**, 1531–1541 (2010). 20356957 10.1242/dev.044750 PMC3188567

[30] Wittmann, D. Spatial analysis of expression patterns predicts genetic interactions at the mid-hindbrain boundary. *PLoS Comput. Biol.* **5**, e1000569 (2009). 19936059 10.1371/journal.pcbi.1000569 PMC2774268

[31] Ferrell, J. & Xiong, W. Bistability in cell signaling: how to make continuous processes discontinuous, and reversible processes irreversible. *Chaos* **11**, 227–236 (2001). 12779456 10.1063/1.1349894

[32] Gritli-Linde, A., Lewis, P., McMahon, A. & Linde, A. The whereabouts of a morphogen: direct evidence for short- and graded long-range activity of hedgehog signaling peptides. *Dev. Biol.* **236**, 364–386, 10.1006/dbio.2001.0336 (2001). 11476578 10.1006/dbio.2001.0336

[33] Cohen, M. et al. Ptch1 and Gli regulate Shh signalling dynamics via multiple mechanisms. *Nat. Commun.* **6**, 6709. 10.1038/ncomms7709 (2015). 25833741 10.1038/ncomms7709 PMC4396374

[34] Gouti, M., Metzis, V., and Briscoe, J. The route to spinal cord cell types: a tale of signals and switches. *Trends Genet.* **31**. 10.1016/j.tig.2015.03.001. (2015). 10.1016/j.tig.2015.03.001 25823696

[35] Martínez, S. The isthmic organizer and brain regionalization. *Int. J. Dev. Biol.* **45**, 367–371 (2001). 11291867

[36] Braun, E. et al. Comprehensive cell atlas of the first-trimester developing human brain. *Science* **382**, eadf1226 (2023). 37824650 10.1126/science.adf1226

[37] Zeng, B. et al. The single-cell and spatial transcriptional landscape of human gastrulation and early brain development. *Cell Stem Cell* **30**, 851–866.e7 (2023). 37192616 10.1016/j.stem.2023.04.016 PMC10241223

[38] Pijuan-Sala, B. et al. A single-cell molecular map of mouse gastrulation and early organogenesis. *Nature* **566**, 490–495 (2019). 30787436 10.1038/s41586-019-0933-9 PMC6522369

[39] Harris, C. R. et al. Array programming with NumPy. *Nature* **585**, 357–362 (2020). 32939066 10.1038/s41586-020-2649-2 PMC7759461

[40] Hao, Y., et al. Integrated analysis of multimodal single-cell data. *Cell*. 10.1016/j.cell.2021.04.048. (2021). 10.1016/j.cell.2021.04.048 PMC8238499 34062119

[41] Waltman, L., and van Eck, N. J. A smart local moving algorithm for large-scale modularity-based community detection. *Eur. Phys. J. B* **86**. 10.1140/epjb/e2013-40829-0. (2013).

[42] McInnes, L. UMAP: Uniform manifold approximation and projection. *J. Open Source Softw.* **3**, 861 (2018).

[43] Waskom, M. L. Seaborn: statistical data visualization. *J. Open Source Softw.* **6**, 3021 (2021).

[44] Shea, M. A. & Ackers, G. K. The OR control system of bacteriophage lambda. A physical-chemical model for gene regulation. *J. Mol. Biol.* **181**, 211–230 (1985). 3157005 10.1016/0022-2836(85)90086-5

[45] Sherman, M. & Cohen, B. Thermodynamic state ensemble models of cis-regulation. *PLoS Comput. Biol.* **8**, e1002407 (2012). 22479169 10.1371/journal.pcbi.1002407 PMC3315449

[46] Merlevede A., et al. A quantitative model of cellular decision making in direct neuronal reprogramming. *Sci. Rep.* 11. 10.1038/s41598-021-81089-8. (2021). 10.1038/s41598-021-81089-8 PMC7810861 33452356

[47] Nobile, M., Paolo Cazzaniga, D., Besozzi, R., Colombo, G. M., and G. Pasi Fuzzy self-tuning PSO: a settings-free algorithm for global optimization. *Swarm Evolut. Comput.* **39**. 10.1016/j.swevo.2017.09.001. (2017).

[48] Byrd, R. H., Peihuang L., J., Nocedal, and C., Zhu. A limited memory algorithm for bound constrained optimization. *SIAM J. Sci. Computing* **16**. 10.1137/0916069. (2003).

[49] Virtanen, P. et al. SciPy 1.0: fundamental algorithms for scientific computing in Python. *Nat. Methods* **17**, 261–272 (2020). 32015543 10.1038/s41592-019-0686-2 PMC7056644

[50] Lam, S. A., Pitrou, and S. Seibert. Numba: A LLVM-Based Python JIT Compiler. In *Proceedings of the Second Workshop on the Llvm Compiler Infrastructure in Hpc*, 1–6. 10.1145/2833157.2833162. (2015).

[51] Kim, J., et al. Wnt signal activation induces midbrain specification through direct binding of the beta-catenin/TCF4 complex to the EN1 promoter in human pluripotent stem cells. *Experim. Mol. Med.* **50**. 10.1038/s12276-018-0044-y. (2018). 10.1038/s12276-018-0044-y PMC5938028 29650976

[52] Lee, J., Platt, K., Censullo, P. & Altaba, A. Gli1 is a target of sonic hedgehog that induces ventral neural tube development. *Development* **124**, 2537–2552 (1997). 9216996 10.1242/dev.124.13.2537

[53] Press, W. H., et al. *Numerical Recipes: The Art of Scientific Computing*, *3rd Edition*. (Cambridge University Press, 2007).

