# Supplementary material for: Combining in vitro and in silico approaches to model neural tube patterning and isthmic organizer formation
Source: NPJ Syst Biol Appl. 2026 Aug 21;12:126. doi: 10.1038/s41540-026-00813-0 (PMC13498569; doi:10.1038/s41540-026-00813-0)
Supplement: Supplementary file 1 [file 41540_2026_813_MOESM1_ESM.pdf]

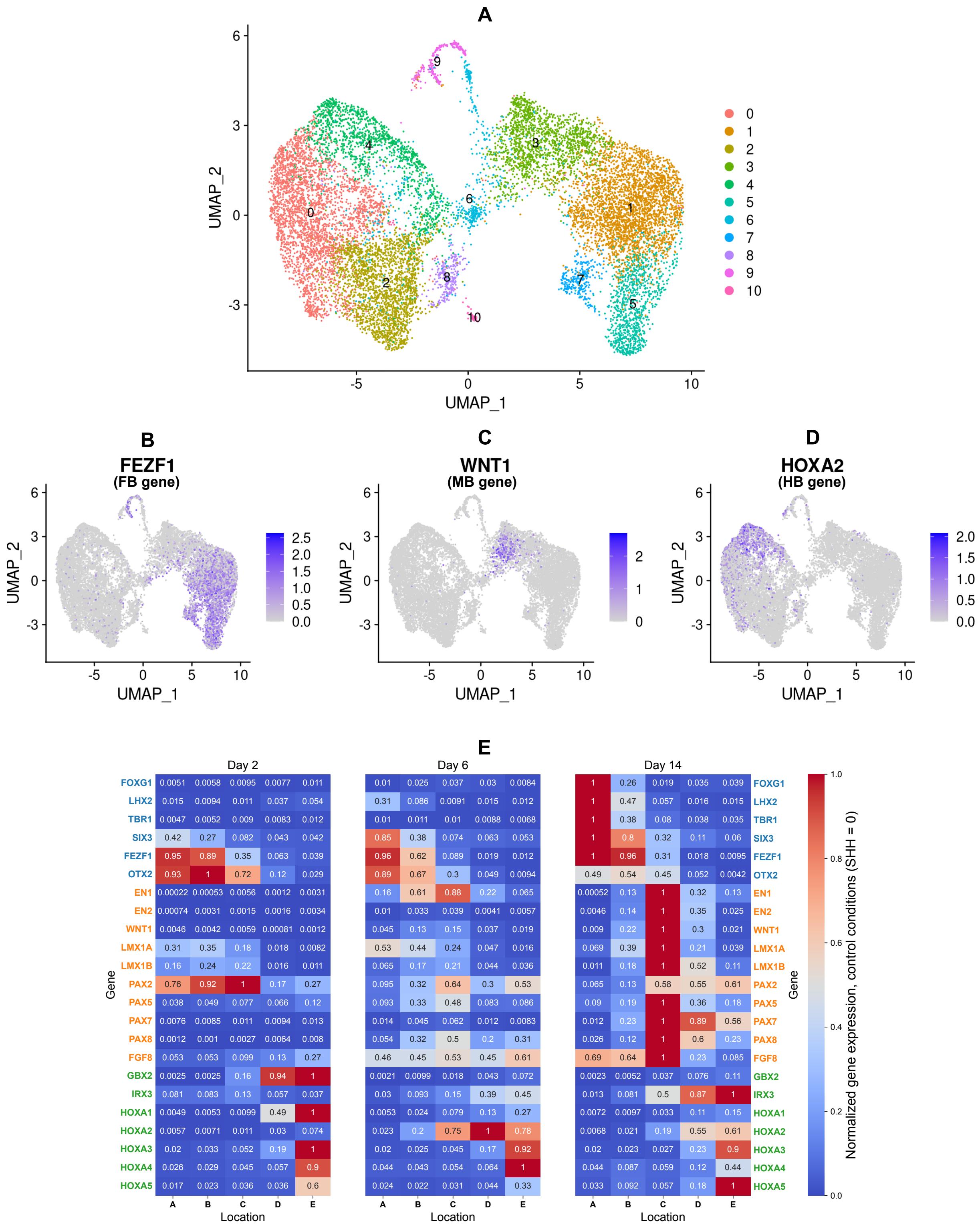

Figure S1, scRNA-seq data processing and bulk data.
(**A**) UMAP distribution of scRNA-seq data from the day 9 non-ventralized dataset, with annotated clusters signifying distinct cell types. (**B**) Expression distribution of the forebrain gene FEZF1. It is mostly expressed to the right, so cells in clusters 1, 5, and 7 in **A** were likely situated in the rostral end of the MiSTR tissue. (**C**) Expression distribution of the midbrain / isthmic organizer gene WNT1. It is mostly expressed in the center, so cells in cluster 3 in **A** were likely situated in the center of the MiSTR tissue. (**D**) Expression distribution of the hindbrain gene HOXA2. It is mostly expressed to the left, so cells in clusters 0, 2, and 4 in **A** were likely situated in the caudal end of the MiSTR tissue. (**E**) Bulk data for the FB, MB, and HB genes. The bulk data exhibit a day 14 A-E expression pattern which is similar to that of the scRNA-seq day 14 data.

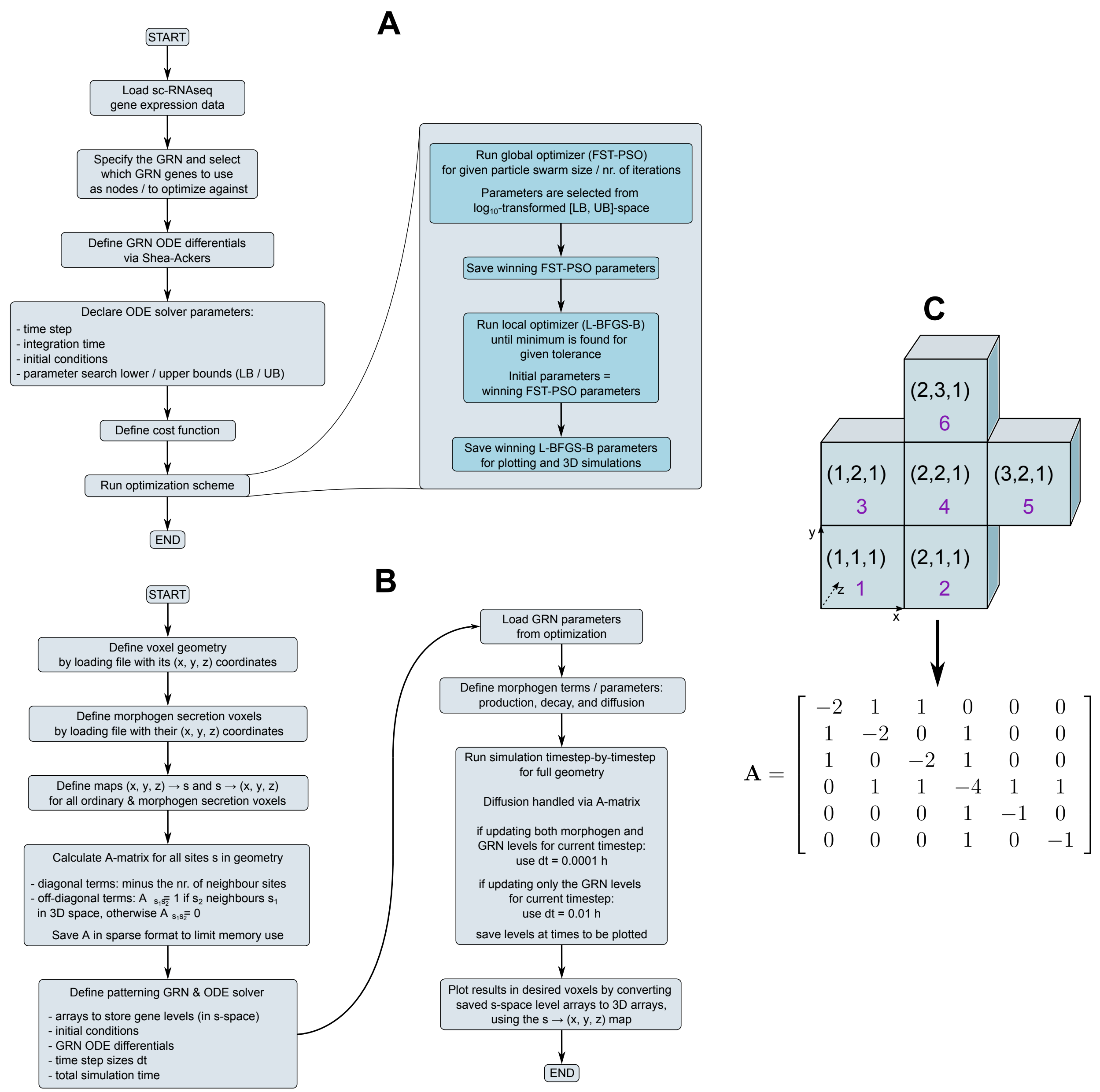

Figure S2, Charts describing optimization and simulation procedures.
(**A**) Flowchart that describes our approach to optimizing GRN model parameters. (**B**) Flowchart that shows our approach to simulating patterning in the 3D voxel geometry. (**C**) A simple example that showcases how the **A**-matrix in equation 11 is calculated for a given 3D geometry, where a particular label s is associated with a particular (x, y, z)-coordinate.

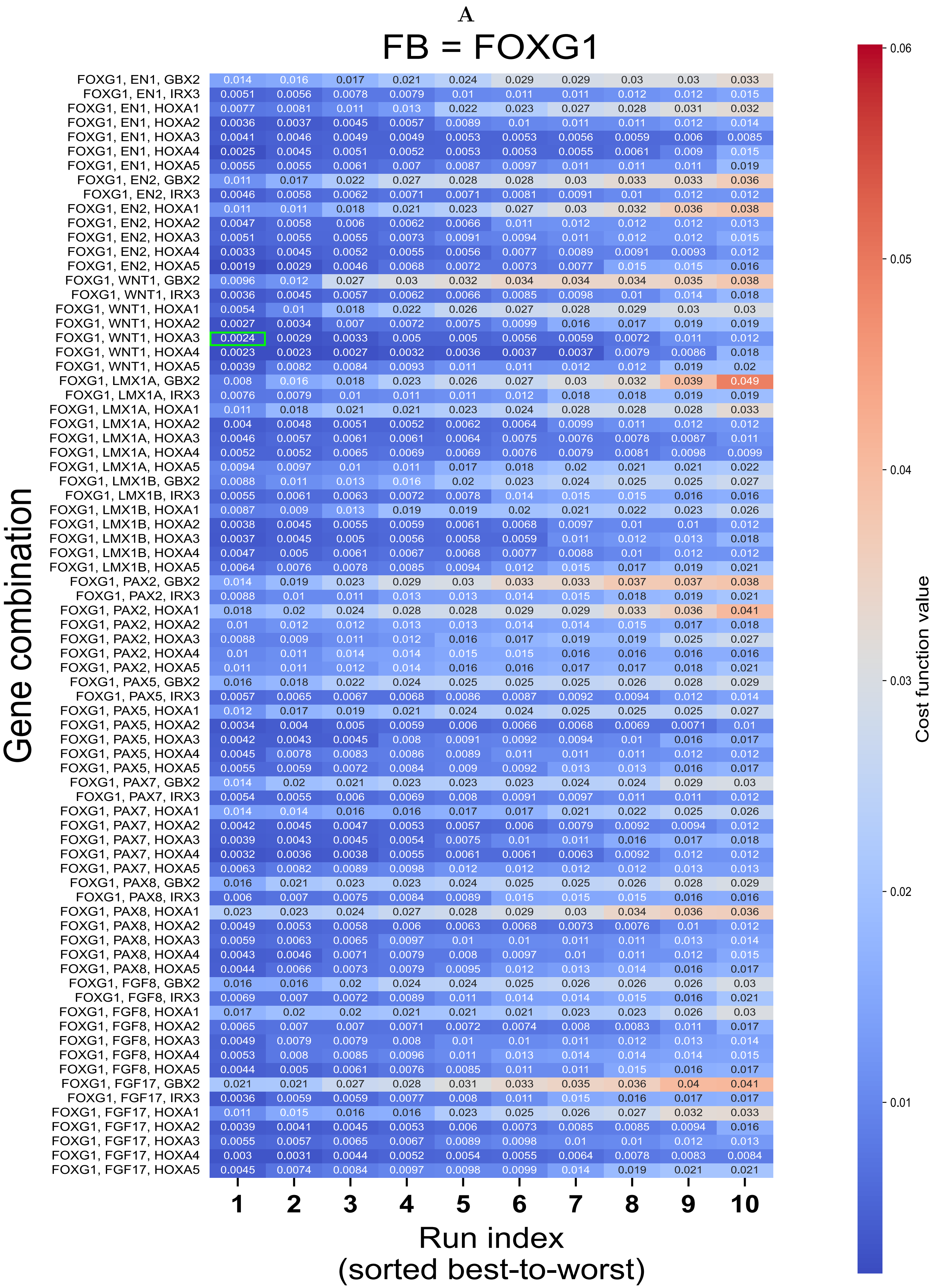
A
FB = FOXG1
FOXG1, EN1, GBX2 0.014 0.016 0.017 0.021 0.024 0.029 0.029 0.03 0.03 0.033
FOXG1, EN1, IRX3 0.0051 0.0056 0.0078 0.0079 0.01 0.011 0.011 0.012 0.012 0.015
FOXG1, EN1, HOXA1 0.0077 0.0081 0.011 0.013 0.022 0.023 0.027 0.028 0.031 0.032
FOXG1, EN1, HOXA2 0.0036 0.0037 0.0045 0.0057 0.0089 0.01 0.011 0.011 0.012 0.014
FOXG1, EN1, HOXA3 0.0041 0.0046 0.0049 0.0049 0.0053 0.0053 0.0056 0.0059 0.006 0.0085
FOXG1, EN1, HOXA4 0.0025 0.0045 0.0051 0.0052 0.0053 0.0053 0.0055 0.0061 0.009 0.015
FOXG1, EN1, HOXA5 0.0055 0.0055 0.0061 0.007 0.0087 0.0097 0.011 0.011 0.011 0.019
FOXG1, EN2, GBX2 0.011 0.017 0.022 0.027 0.028 0.028 0.03 0.033 0.033 0.036
FOXG1, EN2, IRX3 0.0046 0.0058 0.0062 0.0071 0.0071 0.0081 0.0091 0.01 0.012 0.012
FOXG1, EN2, HOXA1 0.011 0.011 0.018 0.021 0.023 0.027 0.03 0.032 0.036 0.038
FOXG1, EN2, HOXA2 0.0047 0.0058 0.006 0.0062 0.0066 0.011 0.012 0.012 0.012 0.013
FOXG1, EN2, HOXA3 0.0051 0.0055 0.0055 0.0073 0.0091 0.0094 0.011 0.012 0.012 0.015
FOXG1, EN2, HOXA4 0.0033 0.0045 0.0052 0.0055 0.0056 0.0077 0.0089 0.0091 0.0093 0.012
FOXG1, EN2, HOXA5 0.0019 0.0029 0.0046 0.0068 0.0072 0.0073 0.0077 0.015 0.015 0.016
FOXG1, WNT1, GBX2 0.0096 0.012 0.027 0.03 0.032 0.034 0.034 0.034 0.035 0.038
FOXG1, WNT1, IRX3 0.0036 0.0045 0.0057 0.0062 0.0066 0.0085 0.0098 0.01 0.014 0.018
FOXG1, WNT1, HOXA1 0.0054 0.01 0.018 0.022 0.026 0.027 0.028 0.029 0.03 0.03
FOXG1, WNT1, HOXA2 0.0027 0.0034 0.007 0.0072 0.0075 0.0099 0.016 0.017 0.019 0.019
FOXG1, WNT1, HOXA3 0.0024 0.0029 0.0033 0.005 0.005 0.0056 0.0059 0.0072 0.011 0.012
FOXG1, WNT1, HOXA4 0.0023 0.0023 0.0027 0.0032 0.0036 0.0037 0.0037 0.0079 0.0086 0.018
FOXG1, WNT1, HOXA5 0.0039 0.0082 0.0084 0.0093 0.011 0.011 0.012 0.012 0.019 0.02
FOXG1, LMX1A, GBX2 0.008 0.016 0.018 0.023 0.026 0.027 0.03 0.032 0.039 0.049
FOXG1, LMX1A, IRX3 0.0076 0.0079 0.01 0.011 0.011 0.012 0.018 0.018 0.019 0.019
FOXG1, LMX1A, HOXA1 0.011 0.018 0.021 0.021 0.023 0.024 0.028 0.028 0.028 0.033
FOXG1, LMX1A, HOXA2 0.004 0.0048 0.0051 0.0052 0.0062 0.0064 0.0099 0.011 0.012 0.012
FOXG1, LMX1A, HOXA3 0.0046 0.0057 0.0061 0.0061 0.0064 0.0075 0.0076 0.0078 0.0087 0.011
FOXG1, LMX1A, HOXA4 0.0052 0.0052 0.0065 0.0069 0.0069 0.0076 0.0079 0.0081 0.0098 0.0099
FOXG1, LMX1A, HOXA5 0.0094 0.0097 0.01 0.011 0.017 0.018 0.02 0.021 0.021 0.022
FOXG1, LMX1B, GBX2 0.0088 0.011 0.013 0.016 0.02 0.023 0.024 0.025 0.025 0.027
FOXG1, LMX1B, IRX3 0.0055 0.0061 0.0063 0.0072 0.0078 0.014 0.015 0.015 0.016 0.016
FOXG1, LMX1B, HOXA1 0.0087 0.009 0.013 0.019 0.019 0.02 0.021 0.022 0.023 0.026
FOXG1, LMX1B, HOXA2 0.0038 0.0045 0.0055 0.0059 0.0061 0.0068 0.0097 0.01 0.01 0.012
FOXG1, LMX1B, HOXA3 0.0037 0.0045 0.005 0.0056 0.0058 0.0059 0.011 0.012 0.013 0.018
FOXG1, LMX1B, HOXA4 0.0047 0.005 0.0061 0.0067 0.0068 0.0077 0.0088 0.01 0.012 0.012
FOXG1, LMX1B, HOXA5 0.0064 0.0076 0.0078 0.0085 0.0094 0.012 0.015 0.017 0.019 0.021
FOXG1, PAX2, GBX2 0.014 0.019 0.023 0.029 0.03 0.033 0.033 0.037 0.037 0.038
FOXG1, PAX2, IRX3 0.0088 0.01 0.011 0.013 0.013 0.014 0.015 0.018 0.019 0.021
FOXG1, PAX2, HOXA1 0.018 0.02 0.024 0.028 0.028 0.029 0.029 0.033 0.036 0.041
FOXG1, PAX2, HOXA2 0.01 0.012 0.012 0.013 0.013 0.014 0.014 0.015 0.017 0.018
FOXG1, PAX2, HOXA3 0.0088 0.009 0.011 0.012 0.016 0.017 0.019 0.019 0.025 0.027
FOXG1, PAX2, HOXA4 0.01 0.011 0.014 0.014 0.015 0.015 0.016 0.016 0.016 0.016
FOXG1, PAX2, HOXA5 0.011 0.011 0.012 0.014 0.016 0.016 0.017 0.017 0.018 0.021
FOXG1, PAX5, GBX2 0.016 0.018 0.022 0.024 0.025 0.025 0.025 0.026 0.028 0.029
FOXG1, PAX5, IRX3 0.0057 0.0065 0.0067 0.0068 0.0086 0.0087 0.0092 0.0094 0.012 0.014
FOXG1, PAX5, HOXA1 0.012 0.017 0.019 0.021 0.024 0.024 0.025 0.025 0.025 0.027
FOXG1, PAX5, HOXA2 0.0034 0.004 0.005 0.0059 0.006 0.0066 0.0068 0.0069 0.0071 0.01
FOXG1, PAX5, HOXA3 0.0042 0.0043 0.0045 0.008 0.0091 0.0092 0.0094 0.01 0.016 0.017
FOXG1, PAX5, HOXA4 0.0045 0.0078 0.0083 0.0086 0.0089 0.011 0.011 0.011 0.012 0.012
FOXG1, PAX5, HOXA5 0.0055 0.0059 0.0072 0.0084 0.009 0.0092 0.013 0.013 0.016 0.017
FOXG1, PAX7, GBX2 0.014 0.02 0.021 0.023 0.023 0.023 0.024 0.024 0.029 0.03
FOXG1, PAX7, IRX3 0.0054 0.0055 0.006 0.0069 0.008 0.0091 0.0097 0.011 0.011 0.012
FOXG1, PAX7, HOXA1 0.014 0.014 0.016 0.016 0.017 0.017 0.021 0.022 0.025 0.026
FOXG1, PAX7, HOXA2 0.0042 0.0045 0.0047 0.0053 0.0057 0.006 0.0079 0.0092 0.0094 0.012
FOXG1, PAX7, HOXA3 0.0039 0.0043 0.0045 0.0054 0.0075 0.01 0.011 0.016 0.017 0.018
FOXG1, PAX7, HOXA4 0.0032 0.0036 0.0038 0.0055 0.0061 0.0061 0.0063 0.0092 0.012 0.012
FOXG1, PAX7, HOXA5 0.0063 0.0082 0.0089 0.0098 0.012 0.012 0.012 0.012 0.013 0.013
FOXG1, PAX8, GBX2 0.016 0.021 0.023 0.023 0.024 0.025 0.025 0.026 0.028 0.029
FOXG1, PAX8, IRX3 0.006 0.007 0.0075 0.0084 0.0089 0.015 0.015 0.015 0.016 0.016
FOXG1, PAX8, HOXA1 0.023 0.023 0.024 0.027 0.028 0.029 0.03 0.034 0.036 0.036
FOXG1, PAX8, HOXA2 0.0049 0.0053 0.0058 0.006 0.0063 0.0068 0.0073 0.0076 0.01 0.012
FOXG1, PAX8, HOXA3 0.0059 0.0063 0.0065 0.0097 0.01 0.01 0.011 0.011 0.013 0.014
FOXG1, PAX8, HOXA4 0.0043 0.0046 0.0071 0.0079 0.008 0.0097 0.01 0.011 0.012 0.015
FOXG1, PAX8, HOXA5 0.0044 0.0066 0.0073 0.0079 0.0095 0.012 0.013 0.014 0.016 0.017
FOXG1, FGF8, GBX2 0.016 0.016 0.02 0.024 0.024 0.025 0.026 0.026 0.026 0.03
FOXG1, FGF8, IRX3 0.0069 0.007 0.0072 0.0089 0.011 0.014 0.014 0.015 0.016 0.021
FOXG1, FGF8, HOXA1 0.017 0.02 0.02 0.021 0.021 0.021 0.023 0.023 0.026 0.03
FOXG1, FGF8, HOXA2 0.0065 0.007 0.007 0.0071 0.0072 0.0074 0.008 0.0083 0.011 0.017
FOXG1, FGF8, HOXA3 0.0049 0.0079 0.0079 0.008 0.01 0.01 0.011 0.012 0.013 0.014
FOXG1, FGF8, HOXA4 0.0053 0.008 0.0085 0.0096 0.011 0.013 0.014 0.014 0.014 0.015
FOXG1, FGF8, HOXA5 0.0044 0.005 0.0061 0.0076 0.0085 0.011 0.011 0.015 0.016 0.017
FOXG1, FGF17, GBX2 0.021 0.021 0.027 0.028 0.031 0.033 0.035 0.036 0.04 0.041
FOXG1, FGF17, IRX3 0.0036 0.0059 0.0059 0.0077 0.008 0.011 0.015 0.016 0.017 0.017
FOXG1, FGF17, HOXA1 0.011 0.015 0.016 0.016 0.023 0.025 0.026 0.027 0.032 0.033
FOXG1, FGF17, HOXA2 0.0039 0.0041 0.0045 0.0053 0.006 0.0073 0.0085 0.0085 0.0094 0.016
FOXG1, FGF17, HOXA3 0.0055 0.0057 0.0065 0.0067 0.0089 0.0098 0.01 0.01 0.012 0.013
FOXG1, FGF17, HOXA4 0.003 0.0031 0.0044 0.0052 0.0054 0.0055 0.0064 0.0078 0.0083 0.0084
FOXG1, FGF17, HOXA5 0.0045 0.0074 0.0084 0.0097 0.0098 0.0099 0.014 0.019 0.021 0.021
Gene combination
1 2 3 4 5 6 7 8 9 10
Run index
(sorted best-to-worst)
0.06
0.05
0.04
0.03
0.02
0.01
Cost function value

B

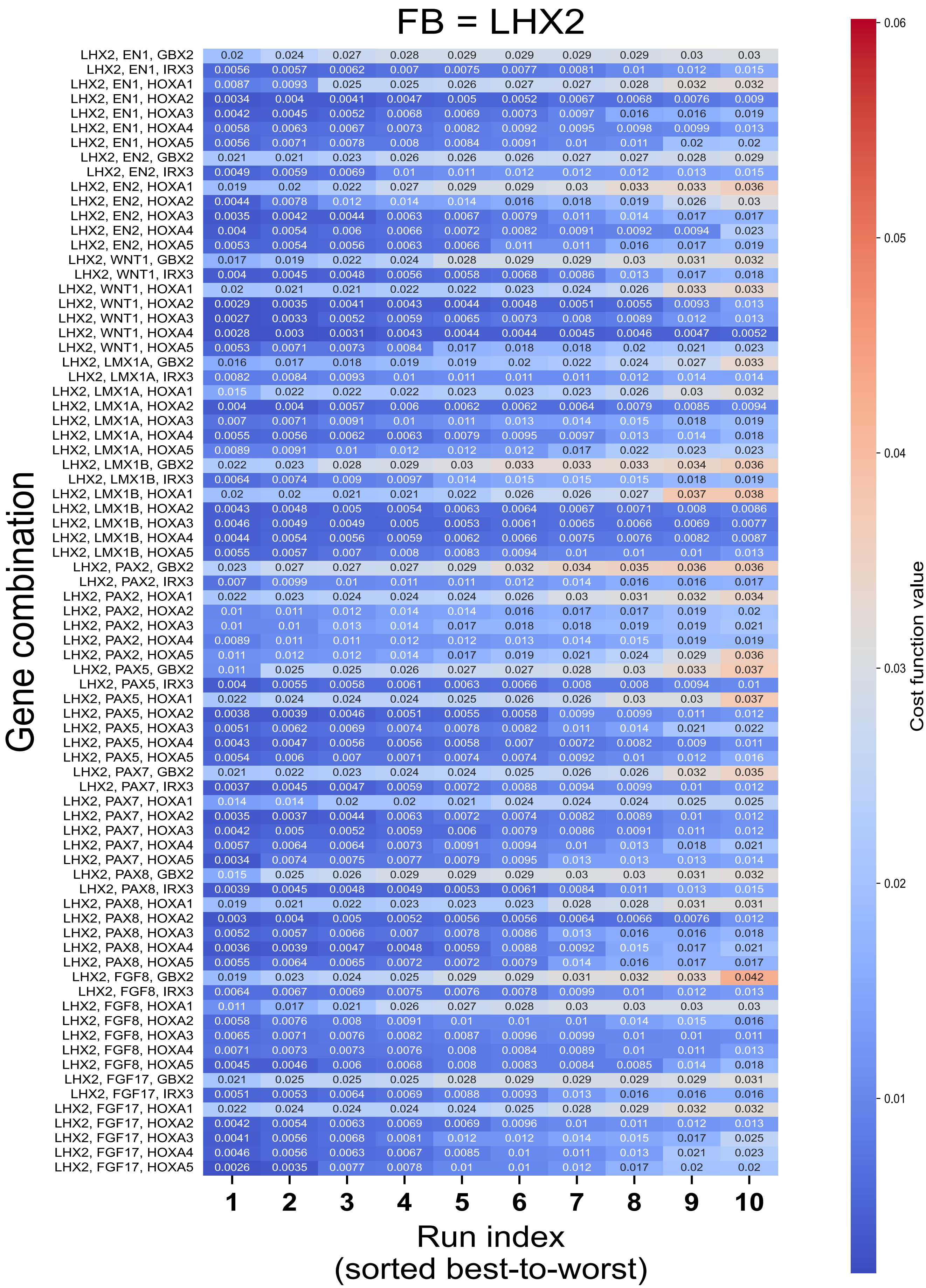
FB = LHX2
Gene combination
Run index
(sorted best-to-worst)
1 2 3 4 5 6 7 8 9 10
Cost function value
0.06
0.05
0.04
0.03
0.02
0.01
LHX2, EN1, GBX2 0.02 0.024 0.027 0.028 0.029 0.029 0.029 0.029 0.03 0.03
LHX2, EN1, IRX3 0.0056 0.0057 0.0062 0.007 0.0075 0.0077 0.0081 0.01 0.012 0.015
LHX2, EN1, HOXA1 0.0087 0.0093 0.025 0.025 0.026 0.027 0.027 0.028 0.032 0.032
LHX2, EN1, HOXA2 0.0034 0.004 0.0041 0.0047 0.005 0.0052 0.0067 0.0068 0.0076 0.009
LHX2, EN1, HOXA3 0.0042 0.0045 0.0052 0.0068 0.0069 0.0073 0.0097 0.016 0.016 0.019
LHX2, EN1, HOXA4 0.0058 0.0063 0.0067 0.0073 0.0082 0.0092 0.0095 0.0098 0.0099 0.013
LHX2, EN1, HOXA5 0.0056 0.0071 0.0078 0.008 0.0084 0.0091 0.01 0.011 0.02 0.02
LHX2, EN2, GBX2 0.021 0.021 0.023 0.026 0.026 0.026 0.027 0.027 0.028 0.029
LHX2, EN2, IRX3 0.0049 0.0059 0.0069 0.01 0.011 0.012 0.012 0.012 0.013 0.015
LHX2, EN2, HOXA1 0.019 0.02 0.022 0.027 0.029 0.029 0.03 0.033 0.033 0.036
LHX2, EN2, HOXA2 0.0044 0.0078 0.012 0.014 0.014 0.016 0.018 0.019 0.026 0.03
LHX2, EN2, HOXA3 0.0035 0.0042 0.0044 0.0063 0.0067 0.0079 0.011 0.014 0.017 0.017
LHX2, EN2, HOXA4 0.004 0.0054 0.006 0.0066 0.0072 0.0082 0.0091 0.0092 0.0094 0.023
LHX2, EN2, HOXA5 0.0053 0.0054 0.0056 0.0063 0.0066 0.011 0.011 0.016 0.017 0.019
LHX2, WNT1, GBX2 0.017 0.019 0.022 0.024 0.028 0.029 0.029 0.03 0.031 0.032
LHX2, WNT1, IRX3 0.004 0.0045 0.0048 0.0056 0.0058 0.0068 0.0086 0.013 0.017 0.018
LHX2, WNT1, HOXA1 0.02 0.021 0.021 0.022 0.022 0.023 0.024 0.026 0.033 0.033
LHX2, WNT1, HOXA2 0.0029 0.0035 0.0041 0.0043 0.0044 0.0048 0.0051 0.0055 0.0093 0.013
LHX2, WNT1, HOXA3 0.0027 0.0033 0.0052 0.0059 0.0065 0.0073 0.008 0.0089 0.012 0.013
LHX2, WNT1, HOXA4 0.0028 0.003 0.0031 0.0043 0.0044 0.0044 0.0045 0.0046 0.0047 0.0052
LHX2, WNT1, HOXA5 0.0053 0.0071 0.0073 0.0084 0.017 0.018 0.018 0.02 0.021 0.023
LHX2, LMX1A, GBX2 0.016 0.017 0.018 0.019 0.019 0.02 0.022 0.024 0.027 0.033
LHX2, LMX1A, IRX3 0.0082 0.0084 0.0093 0.01 0.011 0.011 0.011 0.012 0.014 0.014
LHX2, LMX1A, HOXA1 0.015 0.022 0.022 0.022 0.023 0.023 0.023 0.026 0.03 0.032
LHX2, LMX1A, HOXA2 0.004 0.004 0.0057 0.006 0.0062 0.0062 0.0064 0.0079 0.0085 0.0094
LHX2, LMX1A, HOXA3 0.007 0.0071 0.0091 0.01 0.011 0.013 0.014 0.015 0.018 0.019
LHX2, LMX1A, HOXA4 0.0055 0.0056 0.0062 0.0063 0.0079 0.0095 0.0097 0.013 0.014 0.018
LHX2, LMX1A, HOXA5 0.0089 0.0091 0.01 0.012 0.012 0.012 0.017 0.022 0.023 0.023
LHX2, LMX1B, GBX2 0.022 0.023 0.028 0.029 0.03 0.033 0.033 0.033 0.034 0.036
LHX2, LMX1B, IRX3 0.0064 0.0074 0.009 0.0097 0.014 0.015 0.015 0.015 0.018 0.019
LHX2, LMX1B, HOXA1 0.02 0.02 0.021 0.021 0.022 0.026 0.026 0.027 0.037 0.038
LHX2, LMX1B, HOXA2 0.0043 0.0048 0.005 0.0054 0.0063 0.0064 0.0067 0.0071 0.008 0.0086
LHX2, LMX1B, HOXA3 0.0046 0.0049 0.0049 0.005 0.0053 0.0061 0.0065 0.0066 0.0069 0.0077
LHX2, LMX1B, HOXA4 0.0044 0.0054 0.0056 0.0059 0.0062 0.0066 0.0075 0.0076 0.0082 0.0087
LHX2, LMX1B, HOXA5 0.0055 0.0057 0.007 0.008 0.0083 0.0094 0.01 0.01 0.01 0.013
LHX2, PAX2, GBX2 0.023 0.027 0.027 0.027 0.029 0.032 0.034 0.035 0.036 0.036
LHX2, PAX2, IRX3 0.007 0.0099 0.01 0.011 0.011 0.012 0.014 0.016 0.016 0.017
LHX2, PAX2, HOXA1 0.022 0.023 0.024 0.024 0.024 0.026 0.03 0.031 0.032 0.034
LHX2, PAX2, HOXA2 0.01 0.011 0.012 0.014 0.014 0.016 0.017 0.017 0.019 0.02
LHX2, PAX2, HOXA3 0.01 0.01 0.013 0.014 0.017 0.018 0.018 0.019 0.019 0.021
LHX2, PAX2, HOXA4 0.0089 0.011 0.011 0.012 0.012 0.013 0.014 0.015 0.019 0.019
LHX2, PAX2, HOXA5 0.011 0.012 0.012 0.014 0.017 0.019 0.021 0.024 0.029 0.036
LHX2, PAX5, GBX2 0.011 0.025 0.025 0.026 0.027 0.027 0.028 0.03 0.033 0.037
LHX2, PAX5, IRX3 0.004 0.0055 0.0058 0.0061 0.0063 0.0066 0.008 0.008 0.0094 0.01
LHX2, PAX5, HOXA1 0.022 0.024 0.024 0.024 0.025 0.026 0.026 0.03 0.03 0.037
LHX2, PAX5, HOXA2 0.0038 0.0039 0.0046 0.0051 0.0055 0.0058 0.0099 0.0099 0.011 0.012
LHX2, PAX5, HOXA3 0.0051 0.0062 0.0069 0.0074 0.0078 0.0082 0.011 0.014 0.021 0.022
LHX2, PAX5, HOXA4 0.0043 0.0047 0.0056 0.0056 0.0058 0.007 0.0072 0.0082 0.009 0.011
LHX2, PAX5, HOXA5 0.0054 0.006 0.007 0.0071 0.0074 0.0074 0.0092 0.01 0.012 0.016
LHX2, PAX7, GBX2 0.021 0.022 0.023 0.024 0.024 0.025 0.026 0.026 0.032 0.035
LHX2, PAX7, IRX3 0.0037 0.0045 0.0047 0.0059 0.0072 0.0088 0.0094 0.0099 0.01 0.012
LHX2, PAX7, HOXA1 0.014 0.014 0.02 0.02 0.021 0.024 0.024 0.024 0.025 0.025
LHX2, PAX7, HOXA2 0.0035 0.0037 0.0044 0.0063 0.0072 0.0074 0.0082 0.0089 0.01 0.012
LHX2, PAX7, HOXA3 0.0042 0.005 0.0052 0.0059 0.006 0.0079 0.0086 0.0091 0.011 0.012
LHX2, PAX7, HOXA4 0.0057 0.0064 0.0064 0.0073 0.0091 0.0094 0.01 0.013 0.018 0.021
LHX2, PAX7, HOXA5 0.0034 0.0074 0.0075 0.0077 0.0079 0.0095 0.013 0.013 0.013 0.014
LHX2, PAX8, GBX2 0.015 0.025 0.026 0.029 0.029 0.029 0.03 0.03 0.031 0.032
LHX2, PAX8, IRX3 0.0039 0.0045 0.0048 0.0049 0.0053 0.0061 0.0084 0.011 0.013 0.015
LHX2, PAX8, HOXA1 0.019 0.021 0.022 0.023 0.023 0.023 0.028 0.028 0.031 0.031
LHX2, PAX8, HOXA2 0.003 0.004 0.005 0.0052 0.0056 0.0056 0.0064 0.0066 0.0076 0.012
LHX2, PAX8, HOXA3 0.0052 0.0057 0.0066 0.007 0.0078 0.0086 0.013 0.016 0.016 0.018
LHX2, PAX8, HOXA4 0.0036 0.0039 0.0047 0.0048 0.0059 0.0088 0.0092 0.015 0.017 0.021
LHX2, PAX8, HOXA5 0.0055 0.0064 0.0065 0.0072 0.0072 0.0079 0.014 0.016 0.017 0.017
LHX2, FGF8, GBX2 0.019 0.023 0.024 0.025 0.029 0.029 0.031 0.032 0.033 0.042
LHX2, FGF8, IRX3 0.0064 0.0067 0.0069 0.0075 0.0076 0.0078 0.0099 0.01 0.012 0.013
LHX2, FGF8, HOXA1 0.011 0.017 0.021 0.026 0.027 0.028 0.03 0.03 0.03 0.03
LHX2, FGF8, HOXA2 0.0058 0.0076 0.008 0.0091 0.01 0.01 0.01 0.014 0.015 0.016
LHX2, FGF8, HOXA3 0.0065 0.0071 0.0076 0.0082 0.0087 0.0096 0.0099 0.01 0.01 0.011
LHX2, FGF8, HOXA4 0.0071 0.0073 0.0073 0.0076 0.008 0.0084 0.0089 0.01 0.011 0.013
LHX2, FGF8, HOXA5 0.0045 0.0046 0.006 0.0068 0.008 0.0083 0.0084 0.0085 0.014 0.018
LHX2, FGF17, GBX2 0.021 0.025 0.025 0.025 0.028 0.029 0.029 0.029 0.029 0.031
LHX2, FGF17, IRX3 0.0051 0.0053 0.0064 0.0069 0.0088 0.0093 0.013 0.016 0.016 0.016
LHX2, FGF17, HOXA1 0.022 0.024 0.024 0.024 0.024 0.025 0.028 0.029 0.032 0.032
LHX2, FGF17, HOXA2 0.0042 0.0054 0.0063 0.0069 0.0069 0.0096 0.01 0.011 0.012 0.013
LHX2, FGF17, HOXA3 0.0041 0.0056 0.0068 0.0081 0.012 0.012 0.014 0.015 0.017 0.025
LHX2, FGF17, HOXA4 0.0046 0.0056 0.0063 0.0067 0.0085 0.01 0.011 0.013 0.021 0.023
LHX2, FGF17, HOXA5 0.0026 0.0035 0.0077 0.0078 0.01 0.01 0.012 0.017 0.02 0.02

C

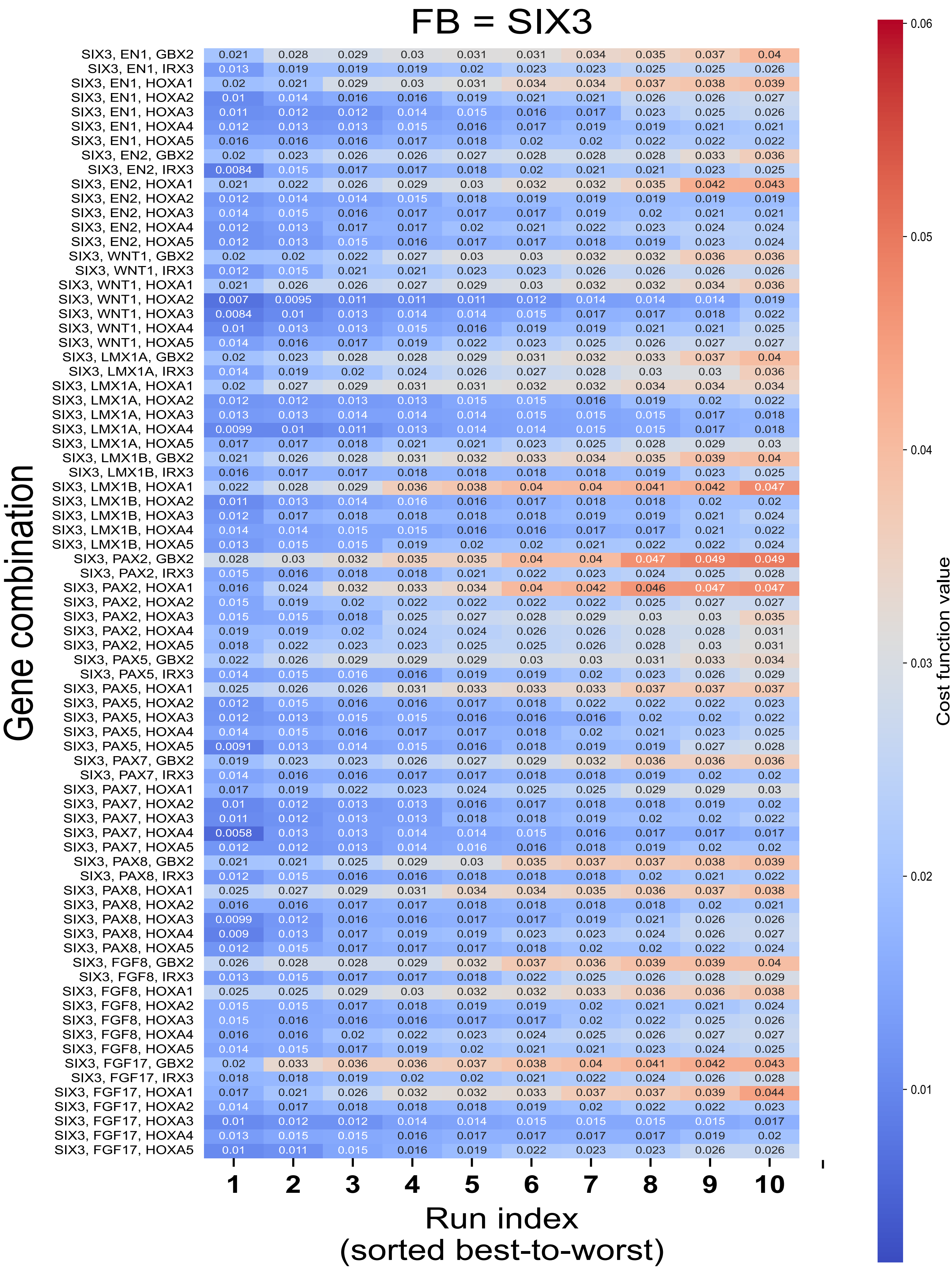
FB = SIX3
Gene combination
Run index
(sorted best-to-worst)
Cost function value
0.06
0.05
0.04
0.03
0.02
0.01
1 2 3 4 5 6 7 8 9 10
SIX3, EN1, GBX2 0.021 0.028 0.029 0.03 0.031 0.031 0.034 0.035 0.037 0.04
SIX3, EN1, IRX3 0.013 0.019 0.019 0.019 0.02 0.023 0.023 0.025 0.025 0.026
SIX3, EN1, HOXA1 0.02 0.021 0.029 0.03 0.031 0.034 0.034 0.037 0.038 0.039
SIX3, EN1, HOXA2 0.01 0.014 0.016 0.016 0.019 0.021 0.021 0.026 0.026 0.027
SIX3, EN1, HOXA3 0.011 0.012 0.012 0.014 0.015 0.016 0.017 0.023 0.025 0.026
SIX3, EN1, HOXA4 0.012 0.013 0.013 0.015 0.016 0.017 0.019 0.019 0.021 0.021
SIX3, EN1, HOXA5 0.016 0.016 0.016 0.017 0.018 0.02 0.02 0.022 0.022 0.022
SIX3, EN2, GBX2 0.02 0.023 0.026 0.026 0.027 0.028 0.028 0.028 0.033 0.036
SIX3, EN2, IRX3 0.0084 0.015 0.017 0.017 0.018 0.02 0.021 0.021 0.023 0.025
SIX3, EN2, HOXA1 0.021 0.022 0.026 0.029 0.03 0.032 0.032 0.035 0.042 0.043
SIX3, EN2, HOXA2 0.012 0.014 0.014 0.015 0.018 0.019 0.019 0.019 0.019 0.019
SIX3, EN2, HOXA3 0.014 0.015 0.016 0.017 0.017 0.017 0.019 0.02 0.021 0.021
SIX3, EN2, HOXA4 0.012 0.013 0.017 0.017 0.02 0.021 0.022 0.023 0.024 0.024
SIX3, EN2, HOXA5 0.012 0.013 0.015 0.016 0.017 0.017 0.018 0.019 0.023 0.024
SIX3, WNT1, GBX2 0.02 0.02 0.022 0.027 0.03 0.03 0.032 0.032 0.036 0.036
SIX3, WNT1, IRX3 0.012 0.015 0.021 0.021 0.023 0.023 0.026 0.026 0.026 0.026
SIX3, WNT1, HOXA1 0.021 0.026 0.026 0.027 0.029 0.03 0.032 0.032 0.034 0.036
SIX3, WNT1, HOXA2 0.007 0.0095 0.011 0.011 0.011 0.012 0.014 0.014 0.014 0.019
SIX3, WNT1, HOXA3 0.0084 0.01 0.013 0.014 0.014 0.015 0.017 0.017 0.018 0.022
SIX3, WNT1, HOXA4 0.01 0.013 0.013 0.015 0.016 0.019 0.019 0.021 0.021 0.025
SIX3, WNT1, HOXA5 0.014 0.016 0.017 0.019 0.022 0.023 0.025 0.026 0.027 0.027
SIX3, LMX1A, GBX2 0.02 0.023 0.028 0.028 0.029 0.031 0.032 0.033 0.037 0.04
SIX3, LMX1A, IRX3 0.014 0.019 0.02 0.024 0.026 0.027 0.028 0.03 0.03 0.036
SIX3, LMX1A, HOXA1 0.02 0.027 0.029 0.031 0.031 0.032 0.032 0.034 0.034 0.034
SIX3, LMX1A, HOXA2 0.012 0.012 0.013 0.013 0.015 0.015 0.016 0.019 0.02 0.022
SIX3, LMX1A, HOXA3 0.013 0.013 0.014 0.014 0.014 0.015 0.015 0.015 0.017 0.018
SIX3, LMX1A, HOXA4 0.0099 0.01 0.011 0.013 0.014 0.014 0.015 0.015 0.017 0.018
SIX3, LMX1A, HOXA5 0.017 0.017 0.018 0.021 0.021 0.023 0.025 0.028 0.029 0.03
SIX3, LMX1B, GBX2 0.021 0.026 0.028 0.031 0.032 0.033 0.034 0.035 0.039 0.04
SIX3, LMX1B, IRX3 0.016 0.017 0.017 0.018 0.018 0.018 0.018 0.019 0.023 0.025
SIX3, LMX1B, HOXA1 0.022 0.028 0.029 0.036 0.038 0.04 0.04 0.041 0.042 0.047
SIX3, LMX1B, HOXA2 0.011 0.013 0.014 0.016 0.016 0.017 0.018 0.018 0.02 0.02
SIX3, LMX1B, HOXA3 0.012 0.017 0.018 0.018 0.018 0.018 0.019 0.019 0.021 0.024
SIX3, LMX1B, HOXA4 0.014 0.014 0.015 0.015 0.016 0.016 0.017 0.017 0.021 0.022
SIX3, LMX1B, HOXA5 0.013 0.015 0.015 0.019 0.02 0.02 0.021 0.022 0.022 0.024
SIX3, PAX2, GBX2 0.028 0.03 0.032 0.035 0.035 0.04 0.04 0.047 0.049 0.049
SIX3, PAX2, IRX3 0.015 0.016 0.018 0.018 0.021 0.022 0.023 0.024 0.025 0.028
SIX3, PAX2, HOXA1 0.016 0.024 0.032 0.033 0.034 0.04 0.042 0.046 0.047 0.047
SIX3, PAX2, HOXA2 0.015 0.019 0.02 0.022 0.022 0.022 0.022 0.025 0.027 0.027
SIX3, PAX2, HOXA3 0.015 0.015 0.018 0.025 0.027 0.028 0.029 0.03 0.03 0.035
SIX3, PAX2, HOXA4 0.019 0.019 0.02 0.024 0.024 0.026 0.026 0.028 0.028 0.031
SIX3, PAX2, HOXA5 0.018 0.022 0.023 0.023 0.025 0.025 0.026 0.028 0.03 0.031
SIX3, PAX5, GBX2 0.022 0.026 0.029 0.029 0.029 0.03 0.03 0.031 0.033 0.034
SIX3, PAX5, IRX3 0.014 0.015 0.016 0.016 0.019 0.019 0.02 0.023 0.026 0.029
SIX3, PAX5, HOXA1 0.025 0.026 0.026 0.031 0.033 0.033 0.033 0.037 0.037 0.037
SIX3, PAX5, HOXA2 0.012 0.015 0.016 0.016 0.017 0.018 0.022 0.022 0.022 0.023
SIX3, PAX5, HOXA3 0.012 0.013 0.015 0.015 0.016 0.016 0.016 0.02 0.02 0.022
SIX3, PAX5, HOXA4 0.014 0.015 0.016 0.017 0.017 0.018 0.02 0.021 0.023 0.025
SIX3, PAX5, HOXA5 0.0091 0.013 0.014 0.015 0.016 0.018 0.019 0.019 0.027 0.028
SIX3, PAX7, GBX2 0.019 0.023 0.023 0.026 0.027 0.029 0.032 0.036 0.036 0.036
SIX3, PAX7, IRX3 0.014 0.016 0.016 0.017 0.017 0.018 0.018 0.019 0.02 0.02
SIX3, PAX7, HOXA1 0.017 0.019 0.022 0.023 0.024 0.025 0.025 0.029 0.029 0.03
SIX3, PAX7, HOXA2 0.01 0.012 0.013 0.013 0.016 0.017 0.018 0.018 0.019 0.02
SIX3, PAX7, HOXA3 0.011 0.012 0.013 0.013 0.018 0.018 0.019 0.02 0.02 0.022
SIX3, PAX7, HOXA4 0.0058 0.013 0.013 0.014 0.014 0.015 0.016 0.017 0.017 0.017
SIX3, PAX7, HOXA5 0.012 0.012 0.013 0.014 0.016 0.016 0.018 0.019 0.02 0.02
SIX3, PAX8, GBX2 0.021 0.021 0.025 0.029 0.03 0.035 0.037 0.037 0.038 0.039
SIX3, PAX8, IRX3 0.012 0.015 0.016 0.016 0.018 0.018 0.018 0.02 0.021 0.022
SIX3, PAX8, HOXA1 0.025 0.027 0.029 0.031 0.034 0.034 0.035 0.036 0.037 0.038
SIX3, PAX8, HOXA2 0.016 0.016 0.017 0.017 0.018 0.018 0.018 0.018 0.02 0.021
SIX3, PAX8, HOXA3 0.0099 0.012 0.016 0.016 0.017 0.017 0.019 0.021 0.026 0.026
SIX3, PAX8, HOXA4 0.009 0.013 0.017 0.019 0.019 0.023 0.023 0.024 0.026 0.027
SIX3, PAX8, HOXA5 0.012 0.015 0.017 0.017 0.017 0.018 0.02 0.02 0.022 0.024
SIX3, FGF8, GBX2 0.026 0.028 0.028 0.029 0.032 0.037 0.036 0.039 0.039 0.04
SIX3, FGF8, IRX3 0.013 0.015 0.017 0.017 0.018 0.022 0.025 0.026 0.028 0.029
SIX3, FGF8, HOXA1 0.025 0.025 0.029 0.03 0.032 0.032 0.033 0.036 0.036 0.038
SIX3, FGF8, HOXA2 0.015 0.015 0.017 0.018 0.019 0.019 0.02 0.021 0.021 0.024
SIX3, FGF8, HOXA3 0.015 0.016 0.016 0.016 0.017 0.017 0.02 0.022 0.025 0.026
SIX3, FGF8, HOXA4 0.016 0.016 0.02 0.022 0.023 0.024 0.025 0.026 0.027 0.027
SIX3, FGF8, HOXA5 0.014 0.015 0.017 0.019 0.02 0.021 0.021 0.023 0.024 0.025
SIX3, FGF17, GBX2 0.02 0.033 0.036 0.036 0.037 0.038 0.04 0.041 0.042 0.043
SIX3, FGF17, IRX3 0.018 0.018 0.019 0.02 0.02 0.021 0.022 0.024 0.026 0.028
SIX3, FGF17, HOXA1 0.017 0.021 0.026 0.032 0.032 0.033 0.037 0.037 0.039 0.044
SIX3, FGF17, HOXA2 0.014 0.017 0.018 0.018 0.018 0.019 0.02 0.022 0.022 0.023
SIX3, FGF17, HOXA3 0.01 0.012 0.012 0.014 0.014 0.015 0.015 0.015 0.015 0.017
SIX3, FGF17, HOXA4 0.013 0.015 0.015 0.016 0.017 0.017 0.017 0.017 0.019 0.02
SIX3, FGF17, HOXA5 0.01 0.011 0.015 0.016 0.019 0.022 0.023 0.023 0.026 0.026

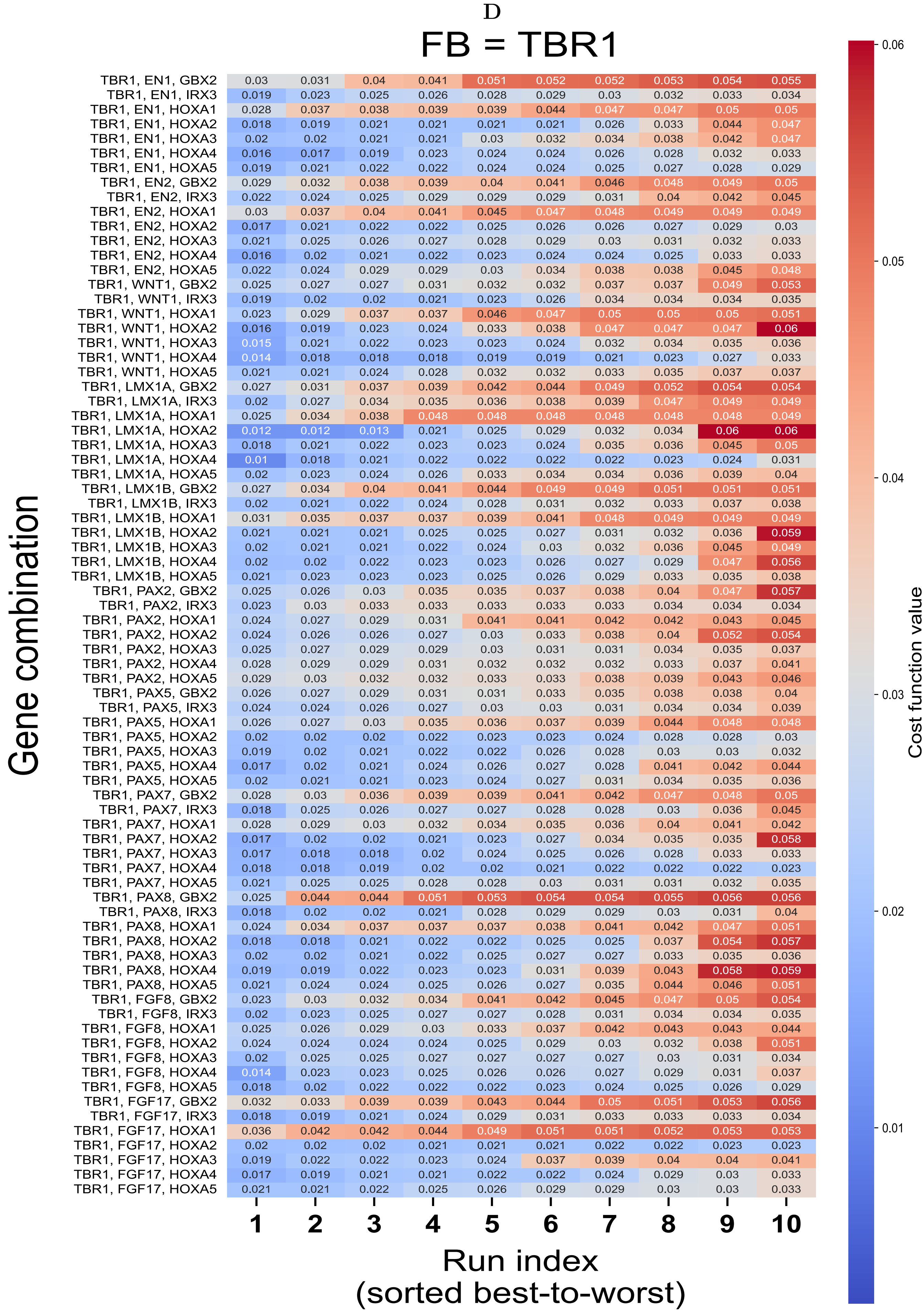
D
FB = TBR1
Gene combination
TBR1, EN1, GBX2 0.03 0.031 0.04 0.041 0.051 0.052 0.052 0.053 0.054 0.055
TBR1, EN1, IRX3 0.019 0.023 0.025 0.026 0.028 0.029 0.03 0.032 0.033 0.034
TBR1, EN1, HOXA1 0.028 0.037 0.038 0.039 0.039 0.044 0.047 0.047 0.05 0.05
TBR1, EN1, HOXA2 0.018 0.019 0.021 0.021 0.021 0.021 0.026 0.033 0.044 0.047
TBR1, EN1, HOXA3 0.02 0.02 0.021 0.021 0.03 0.032 0.034 0.038 0.042 0.047
TBR1, EN1, HOXA4 0.016 0.017 0.019 0.023 0.024 0.024 0.026 0.028 0.032 0.033
TBR1, EN1, HOXA5 0.019 0.021 0.022 0.022 0.024 0.024 0.025 0.027 0.028 0.029
TBR1, EN2, GBX2 0.029 0.032 0.038 0.039 0.04 0.041 0.046 0.048 0.049 0.05
TBR1, EN2, IRX3 0.022 0.024 0.025 0.029 0.029 0.029 0.031 0.04 0.042 0.045
TBR1, EN2, HOXA1 0.03 0.037 0.04 0.041 0.045 0.047 0.048 0.049 0.049 0.049
TBR1, EN2, HOXA2 0.017 0.021 0.022 0.022 0.025 0.026 0.026 0.027 0.029 0.03
TBR1, EN2, HOXA3 0.021 0.025 0.026 0.027 0.028 0.029 0.03 0.031 0.032 0.033
TBR1, EN2, HOXA4 0.016 0.02 0.021 0.022 0.023 0.024 0.024 0.025 0.033 0.033
TBR1, EN2, HOXA5 0.022 0.024 0.029 0.029 0.03 0.034 0.038 0.038 0.045 0.048
TBR1, WNT1, GBX2 0.025 0.027 0.027 0.031 0.032 0.032 0.037 0.037 0.049 0.053
TBR1, WNT1, IRX3 0.019 0.02 0.02 0.021 0.023 0.026 0.034 0.034 0.034 0.035
TBR1, WNT1, HOXA1 0.023 0.029 0.037 0.037 0.046 0.047 0.05 0.05 0.05 0.051
TBR1, WNT1, HOXA2 0.016 0.019 0.023 0.024 0.033 0.038 0.047 0.047 0.047 0.06
TBR1, WNT1, HOXA3 0.015 0.021 0.022 0.023 0.023 0.024 0.032 0.034 0.035 0.036
TBR1, WNT1, HOXA4 0.014 0.018 0.018 0.018 0.019 0.019 0.021 0.023 0.027 0.033
TBR1, WNT1, HOXA5 0.021 0.021 0.024 0.028 0.032 0.032 0.033 0.035 0.037 0.037
TBR1, LMX1A, GBX2 0.027 0.031 0.037 0.039 0.042 0.044 0.049 0.052 0.054 0.054
TBR1, LMX1A, IRX3 0.02 0.027 0.034 0.035 0.036 0.038 0.039 0.047 0.049 0.049
TBR1, LMX1A, HOXA1 0.025 0.034 0.038 0.048 0.048 0.048 0.048 0.048 0.048 0.049
TBR1, LMX1A, HOXA2 0.012 0.012 0.013 0.021 0.025 0.029 0.032 0.034 0.06 0.06
TBR1, LMX1A, HOXA3 0.018 0.021 0.022 0.023 0.023 0.024 0.035 0.036 0.045 0.05
TBR1, LMX1A, HOXA4 0.01 0.018 0.021 0.022 0.022 0.022 0.022 0.023 0.024 0.031
TBR1, LMX1A, HOXA5 0.02 0.023 0.024 0.026 0.033 0.034 0.034 0.036 0.039 0.04
TBR1, LMX1B, GBX2 0.027 0.034 0.04 0.041 0.044 0.049 0.049 0.051 0.051 0.051
TBR1, LMX1B, IRX3 0.02 0.021 0.022 0.024 0.028 0.031 0.032 0.033 0.037 0.038
TBR1, LMX1B, HOXA1 0.031 0.035 0.037 0.037 0.039 0.041 0.048 0.049 0.049 0.049
TBR1, LMX1B, HOXA2 0.021 0.021 0.021 0.025 0.025 0.027 0.031 0.032 0.036 0.059
TBR1, LMX1B, HOXA3 0.02 0.021 0.021 0.022 0.024 0.03 0.032 0.036 0.045 0.049
TBR1, LMX1B, HOXA4 0.02 0.02 0.022 0.023 0.023 0.026 0.027 0.029 0.047 0.056
TBR1, LMX1B, HOXA5 0.021 0.023 0.023 0.023 0.025 0.026 0.029 0.033 0.035 0.038
TBR1, PAX2, GBX2 0.025 0.026 0.03 0.035 0.035 0.037 0.038 0.04 0.047 0.057
TBR1, PAX2, IRX3 0.023 0.03 0.033 0.033 0.033 0.033 0.033 0.034 0.034 0.034
TBR1, PAX2, HOXA1 0.024 0.027 0.029 0.031 0.041 0.041 0.042 0.042 0.043 0.045
TBR1, PAX2, HOXA2 0.024 0.026 0.026 0.027 0.03 0.033 0.038 0.04 0.052 0.054
TBR1, PAX2, HOXA3 0.025 0.027 0.029 0.029 0.03 0.031 0.031 0.034 0.035 0.037
TBR1, PAX2, HOXA4 0.028 0.029 0.029 0.031 0.032 0.032 0.032 0.033 0.037 0.041
TBR1, PAX2, HOXA5 0.029 0.03 0.032 0.032 0.033 0.033 0.038 0.039 0.043 0.046
TBR1, PAX5, GBX2 0.026 0.027 0.029 0.031 0.031 0.033 0.035 0.038 0.038 0.04
TBR1, PAX5, IRX3 0.024 0.024 0.026 0.027 0.03 0.03 0.031 0.034 0.034 0.039
TBR1, PAX5, HOXA1 0.026 0.027 0.03 0.035 0.036 0.037 0.039 0.044 0.048 0.048
TBR1, PAX5, HOXA2 0.02 0.02 0.02 0.022 0.023 0.023 0.024 0.028 0.028 0.03
TBR1, PAX5, HOXA3 0.019 0.02 0.021 0.022 0.022 0.026 0.028 0.03 0.03 0.032
TBR1, PAX5, HOXA4 0.017 0.02 0.021 0.024 0.026 0.027 0.028 0.041 0.042 0.044
TBR1, PAX5, HOXA5 0.02 0.021 0.021 0.023 0.024 0.027 0.031 0.034 0.035 0.036
TBR1, PAX7, GBX2 0.028 0.03 0.036 0.039 0.039 0.041 0.042 0.047 0.048 0.05
TBR1, PAX7, IRX3 0.018 0.025 0.026 0.027 0.027 0.028 0.028 0.03 0.036 0.045
TBR1, PAX7, HOXA1 0.028 0.029 0.03 0.032 0.034 0.035 0.036 0.04 0.041 0.042
TBR1, PAX7, HOXA2 0.017 0.02 0.02 0.021 0.023 0.027 0.034 0.035 0.035 0.058
TBR1, PAX7, HOXA3 0.017 0.018 0.018 0.02 0.024 0.025 0.026 0.028 0.033 0.033
TBR1, PAX7, HOXA4 0.018 0.018 0.019 0.02 0.02 0.021 0.022 0.022 0.022 0.023
TBR1, PAX7, HOXA5 0.021 0.025 0.025 0.028 0.028 0.03 0.031 0.031 0.032 0.035
TBR1, PAX8, GBX2 0.025 0.044 0.044 0.051 0.053 0.054 0.054 0.055 0.056 0.056
TBR1, PAX8, IRX3 0.018 0.02 0.02 0.021 0.028 0.029 0.029 0.03 0.031 0.04
TBR1, PAX8, HOXA1 0.024 0.034 0.037 0.037 0.037 0.038 0.041 0.042 0.047 0.051
TBR1, PAX8, HOXA2 0.018 0.018 0.021 0.022 0.022 0.025 0.025 0.037 0.054 0.057
TBR1, PAX8, HOXA3 0.02 0.02 0.021 0.022 0.025 0.027 0.027 0.033 0.035 0.036
TBR1, PAX8, HOXA4 0.019 0.019 0.022 0.023 0.023 0.031 0.039 0.043 0.058 0.059
TBR1, PAX8, HOXA5 0.021 0.024 0.024 0.025 0.026 0.027 0.035 0.044 0.046 0.051
TBR1, FGF8, GBX2 0.023 0.03 0.032 0.034 0.041 0.042 0.045 0.047 0.05 0.054
TBR1, FGF8, IRX3 0.02 0.023 0.025 0.027 0.027 0.028 0.031 0.034 0.034 0.035
TBR1, FGF8, HOXA1 0.025 0.026 0.029 0.03 0.033 0.037 0.042 0.043 0.043 0.044
TBR1, FGF8, HOXA2 0.024 0.024 0.024 0.024 0.025 0.029 0.03 0.032 0.038 0.051
TBR1, FGF8, HOXA3 0.02 0.025 0.025 0.027 0.027 0.027 0.027 0.03 0.031 0.034
TBR1, FGF8, HOXA4 0.014 0.023 0.023 0.025 0.026 0.026 0.027 0.029 0.031 0.037
TBR1, FGF8, HOXA5 0.018 0.02 0.022 0.022 0.022 0.023 0.024 0.025 0.026 0.029
TBR1, FGF17, GBX2 0.032 0.033 0.039 0.039 0.043 0.044 0.05 0.051 0.053 0.056
TBR1, FGF17, IRX3 0.018 0.019 0.021 0.024 0.029 0.031 0.033 0.033 0.033 0.034
TBR1, FGF17, HOXA1 0.036 0.042 0.042 0.044 0.049 0.051 0.051 0.052 0.053 0.053
TBR1, FGF17, HOXA2 0.02 0.02 0.02 0.021 0.021 0.021 0.022 0.022 0.023 0.023
TBR1, FGF17, HOXA3 0.019 0.022 0.022 0.023 0.024 0.037 0.039 0.04 0.04 0.041
TBR1, FGF17, HOXA4 0.017 0.019 0.021 0.021 0.022 0.022 0.024 0.029 0.03 0.033
TBR1, FGF17, HOXA5 0.021 0.021 0.022 0.025 0.026 0.029 0.029 0.03 0.03 0.033
1 2 3 4 5 6 7 8 9 10
Run index
(sorted best-to-worst)
Cost function value
0.06
0.05
0.04
0.03
0.02
0.01

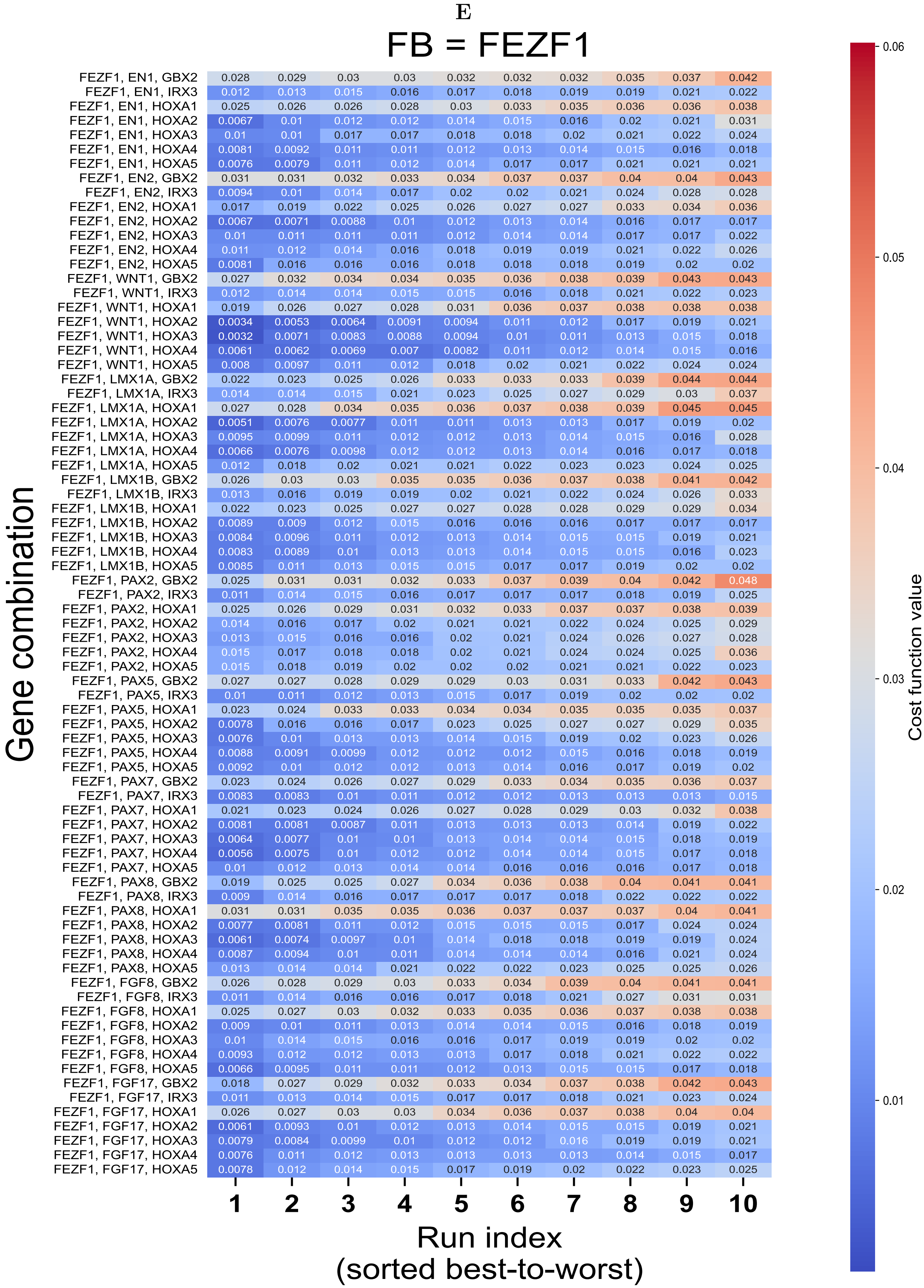
E
FB = FEZF1
Gene combination
Run index
(sorted best-to-worst)
Cost function value
0.06
0.05
0.04
0.03
0.02
0.01
1 2 3 4 5 6 7 8 9 10
FEZF1, EN1, GBX2 0.028 0.029 0.03 0.03 0.032 0.032 0.032 0.035 0.037 0.042
FEZF1, EN1, IRX3 0.012 0.013 0.015 0.016 0.017 0.018 0.019 0.019 0.021 0.022
FEZF1, EN1, HOXA1 0.025 0.026 0.026 0.028 0.03 0.033 0.035 0.036 0.036 0.038
FEZF1, EN1, HOXA2 0.0067 0.01 0.012 0.012 0.014 0.015 0.016 0.02 0.021 0.031
FEZF1, EN1, HOXA3 0.01 0.01 0.017 0.017 0.018 0.018 0.02 0.021 0.022 0.024
FEZF1, EN1, HOXA4 0.0081 0.0092 0.011 0.011 0.012 0.013 0.014 0.015 0.016 0.018
FEZF1, EN1, HOXA5 0.0076 0.0079 0.011 0.012 0.014 0.017 0.017 0.021 0.021 0.021
FEZF1, EN2, GBX2 0.031 0.031 0.032 0.033 0.034 0.037 0.037 0.04 0.04 0.043
FEZF1, EN2, IRX3 0.0094 0.01 0.014 0.017 0.02 0.02 0.021 0.024 0.028 0.028
FEZF1, EN2, HOXA1 0.017 0.019 0.022 0.025 0.026 0.027 0.027 0.033 0.034 0.036
FEZF1, EN2, HOXA2 0.0067 0.0071 0.0088 0.01 0.012 0.013 0.014 0.016 0.017 0.017
FEZF1, EN2, HOXA3 0.01 0.011 0.011 0.011 0.012 0.014 0.014 0.017 0.017 0.022
FEZF1, EN2, HOXA4 0.011 0.012 0.014 0.016 0.018 0.019 0.019 0.021 0.022 0.026
FEZF1, EN2, HOXA5 0.0081 0.016 0.016 0.016 0.018 0.018 0.018 0.019 0.02 0.02
FEZF1, WNT1, GBX2 0.027 0.032 0.034 0.034 0.035 0.036 0.038 0.039 0.043 0.043
FEZF1, WNT1, IRX3 0.012 0.014 0.014 0.015 0.015 0.016 0.018 0.021 0.022 0.023
FEZF1, WNT1, HOXA1 0.019 0.026 0.027 0.028 0.031 0.036 0.037 0.038 0.038 0.038
FEZF1, WNT1, HOXA2 0.0034 0.0053 0.0064 0.0091 0.0094 0.011 0.012 0.017 0.019 0.021
FEZF1, WNT1, HOXA3 0.0032 0.0071 0.0083 0.0088 0.0094 0.01 0.011 0.013 0.015 0.018
FEZF1, WNT1, HOXA4 0.0061 0.0062 0.0069 0.007 0.0082 0.011 0.012 0.014 0.015 0.016
FEZF1, WNT1, HOXA5 0.008 0.0097 0.011 0.012 0.018 0.02 0.021 0.022 0.024 0.024
FEZF1, LMX1A, GBX2 0.022 0.023 0.025 0.026 0.033 0.033 0.033 0.039 0.044 0.044
FEZF1, LMX1A, IRX3 0.014 0.014 0.015 0.021 0.023 0.025 0.027 0.029 0.03 0.037
FEZF1, LMX1A, HOXA1 0.027 0.028 0.034 0.035 0.036 0.037 0.038 0.039 0.045 0.045
FEZF1, LMX1A, HOXA2 0.0051 0.0076 0.0077 0.011 0.011 0.013 0.013 0.017 0.019 0.02
FEZF1, LMX1A, HOXA3 0.0095 0.0099 0.011 0.012 0.012 0.013 0.014 0.015 0.016 0.028
FEZF1, LMX1A, HOXA4 0.0066 0.0076 0.0098 0.012 0.012 0.013 0.014 0.016 0.017 0.018
FEZF1, LMX1A, HOXA5 0.012 0.018 0.02 0.021 0.021 0.022 0.023 0.023 0.024 0.025
FEZF1, LMX1B, GBX2 0.026 0.03 0.03 0.035 0.035 0.036 0.037 0.038 0.041 0.042
FEZF1, LMX1B, IRX3 0.013 0.016 0.019 0.019 0.02 0.021 0.022 0.024 0.026 0.033
FEZF1, LMX1B, HOXA1 0.022 0.023 0.025 0.027 0.027 0.028 0.028 0.029 0.029 0.034
FEZF1, LMX1B, HOXA2 0.0089 0.009 0.012 0.015 0.016 0.016 0.016 0.017 0.017 0.017
FEZF1, LMX1B, HOXA3 0.0084 0.0096 0.011 0.012 0.013 0.014 0.015 0.015 0.019 0.021
FEZF1, LMX1B, HOXA4 0.0083 0.0089 0.01 0.013 0.013 0.014 0.015 0.015 0.016 0.023
FEZF1, LMX1B, HOXA5 0.0085 0.011 0.013 0.015 0.015 0.017 0.017 0.019 0.02 0.02
FEZF1, PAX2, GBX2 0.025 0.031 0.031 0.032 0.033 0.037 0.039 0.04 0.042 0.048
FEZF1, PAX2, IRX3 0.011 0.014 0.015 0.016 0.017 0.017 0.017 0.018 0.019 0.025
FEZF1, PAX2, HOXA1 0.025 0.026 0.029 0.031 0.032 0.033 0.037 0.037 0.038 0.039
FEZF1, PAX2, HOXA2 0.014 0.016 0.017 0.02 0.021 0.021 0.022 0.024 0.025 0.029
FEZF1, PAX2, HOXA3 0.013 0.015 0.016 0.016 0.02 0.021 0.024 0.026 0.027 0.028
FEZF1, PAX2, HOXA4 0.015 0.017 0.018 0.018 0.02 0.021 0.024 0.024 0.025 0.036
FEZF1, PAX2, HOXA5 0.015 0.018 0.019 0.02 0.02 0.02 0.021 0.021 0.022 0.023
FEZF1, PAX5, GBX2 0.027 0.027 0.028 0.029 0.029 0.03 0.031 0.033 0.042 0.043
FEZF1, PAX5, IRX3 0.01 0.011 0.012 0.013 0.015 0.017 0.019 0.02 0.02 0.02
FEZF1, PAX5, HOXA1 0.023 0.024 0.033 0.033 0.034 0.034 0.035 0.035 0.035 0.037
FEZF1, PAX5, HOXA2 0.0078 0.016 0.016 0.017 0.023 0.025 0.027 0.027 0.029 0.035
FEZF1, PAX5, HOXA3 0.0076 0.01 0.013 0.013 0.014 0.015 0.019 0.02 0.023 0.026
FEZF1, PAX5, HOXA4 0.0088 0.0091 0.0099 0.012 0.012 0.012 0.015 0.016 0.018 0.019
FEZF1, PAX5, HOXA5 0.0092 0.01 0.012 0.012 0.013 0.014 0.016 0.017 0.019 0.02
FEZF1, PAX7, GBX2 0.023 0.024 0.026 0.027 0.029 0.033 0.034 0.035 0.036 0.037
FEZF1, PAX7, IRX3 0.0083 0.0083 0.01 0.011 0.012 0.012 0.013 0.013 0.013 0.015
FEZF1, PAX7, HOXA1 0.021 0.023 0.024 0.026 0.027 0.028 0.029 0.03 0.032 0.038
FEZF1, PAX7, HOXA2 0.0081 0.0081 0.0087 0.011 0.013 0.013 0.013 0.014 0.019 0.022
FEZF1, PAX7, HOXA3 0.0064 0.0077 0.01 0.01 0.013 0.014 0.014 0.015 0.018 0.019
FEZF1, PAX7, HOXA4 0.0056 0.0075 0.01 0.012 0.012 0.014 0.014 0.015 0.017 0.018
FEZF1, PAX7, HOXA5 0.01 0.012 0.013 0.014 0.014 0.016 0.016 0.016 0.017 0.018
FEZF1, PAX8, GBX2 0.019 0.025 0.025 0.027 0.034 0.036 0.038 0.04 0.041 0.041
FEZF1, PAX8, IRX3 0.009 0.014 0.016 0.017 0.017 0.017 0.018 0.022 0.022 0.022
FEZF1, PAX8, HOXA1 0.031 0.031 0.035 0.035 0.036 0.037 0.037 0.037 0.04 0.041
FEZF1, PAX8, HOXA2 0.0077 0.0081 0.011 0.012 0.015 0.015 0.015 0.017 0.024 0.024
FEZF1, PAX8, HOXA3 0.0061 0.0074 0.0097 0.01 0.014 0.018 0.018 0.019 0.019 0.024
FEZF1, PAX8, HOXA4 0.0087 0.0094 0.01 0.011 0.014 0.014 0.014 0.016 0.021 0.024
FEZF1, PAX8, HOXA5 0.013 0.014 0.014 0.021 0.022 0.022 0.023 0.025 0.025 0.026
FEZF1, FGF8, GBX2 0.026 0.028 0.029 0.03 0.033 0.034 0.039 0.04 0.041 0.041
FEZF1, FGF8, IRX3 0.011 0.014 0.016 0.016 0.017 0.018 0.021 0.027 0.031 0.031
FEZF1, FGF8, HOXA1 0.025 0.027 0.03 0.032 0.033 0.035 0.036 0.037 0.038 0.038
FEZF1, FGF8, HOXA2 0.009 0.01 0.011 0.013 0.014 0.014 0.015 0.016 0.018 0.019
FEZF1, FGF8, HOXA3 0.01 0.014 0.015 0.016 0.016 0.017 0.019 0.019 0.02 0.02
FEZF1, FGF8, HOXA4 0.0093 0.012 0.012 0.013 0.013 0.017 0.018 0.021 0.022 0.022
FEZF1, FGF8, HOXA5 0.0066 0.0095 0.011 0.011 0.012 0.013 0.015 0.015 0.017 0.018
FEZF1, FGF17, GBX2 0.018 0.027 0.029 0.032 0.033 0.034 0.037 0.038 0.042 0.043
FEZF1, FGF17, IRX3 0.011 0.013 0.014 0.015 0.017 0.017 0.018 0.021 0.023 0.024
FEZF1, FGF17, HOXA1 0.026 0.027 0.03 0.03 0.034 0.036 0.037 0.038 0.04 0.04
FEZF1, FGF17, HOXA2 0.0061 0.0093 0.01 0.012 0.013 0.014 0.015 0.015 0.019 0.021
FEZF1, FGF17, HOXA3 0.0079 0.0084 0.0099 0.01 0.012 0.012 0.016 0.019 0.019 0.021
FEZF1, FGF17, HOXA4 0.0076 0.011 0.012 0.013 0.013 0.013 0.013 0.014 0.015 0.017
FEZF1, FGF17, HOXA5 0.0078 0.012 0.014 0.015 0.017 0.019 0.02 0.022 0.023 0.025

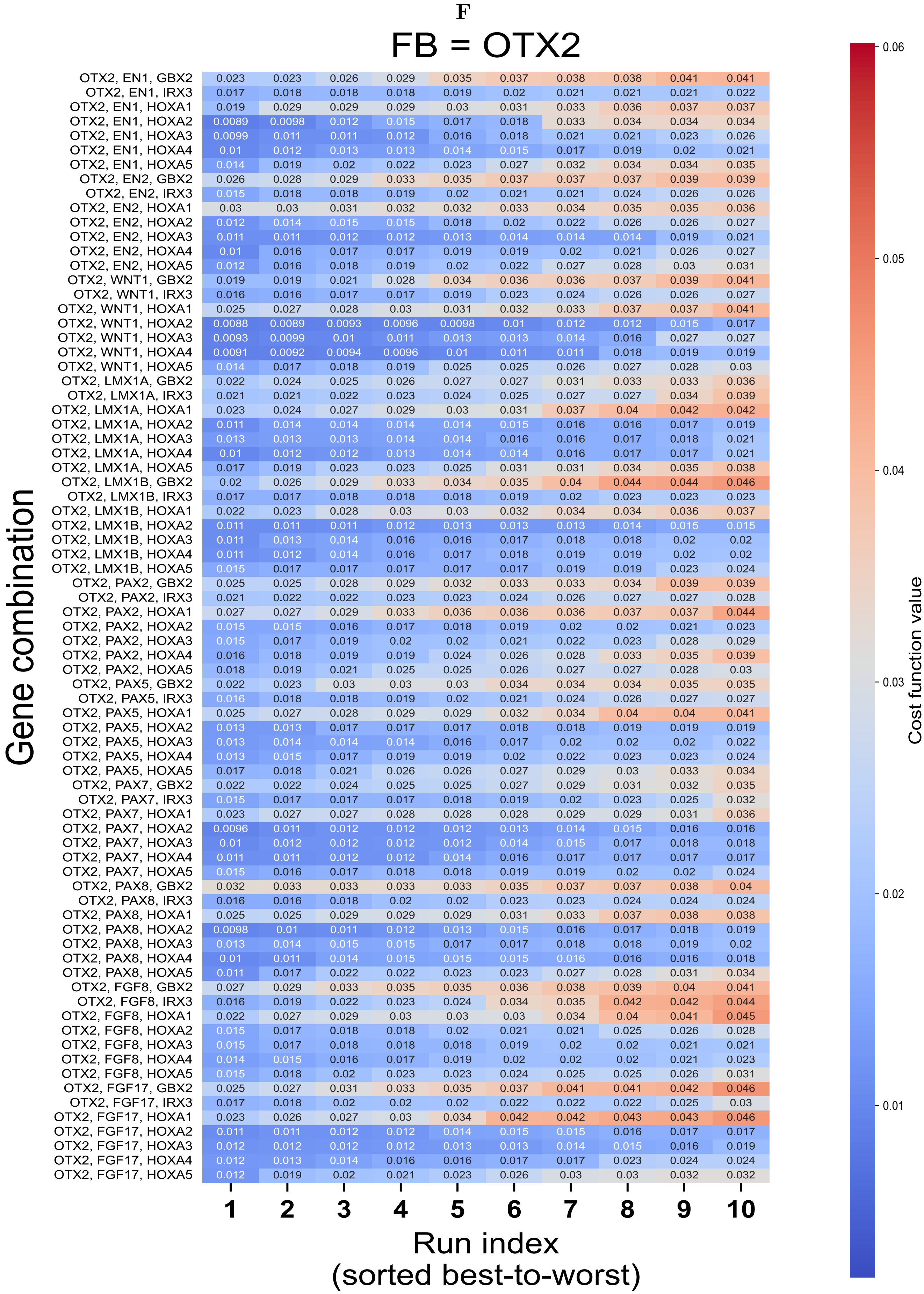
F
FB = OTX2
Gene combination
Run index
(sorted best-to-worst)
1 2 3 4 5 6 7 8 9 10
Cost function value
0.06
0.05
0.04
0.03
0.02
0.01
OTX2, EN1, GBX2 0.023 0.023 0.026 0.029 0.035 0.037 0.038 0.038 0.041 0.041
OTX2, EN1, IRX3 0.017 0.018 0.018 0.018 0.019 0.02 0.021 0.021 0.021 0.022
OTX2, EN1, HOXA1 0.019 0.029 0.029 0.029 0.03 0.031 0.033 0.036 0.037 0.037
OTX2, EN1, HOXA2 0.0089 0.0098 0.012 0.015 0.017 0.018 0.033 0.034 0.034 0.034
OTX2, EN1, HOXA3 0.0099 0.011 0.011 0.012 0.016 0.018 0.021 0.021 0.023 0.026
OTX2, EN1, HOXA4 0.01 0.012 0.013 0.013 0.014 0.015 0.017 0.019 0.02 0.021
OTX2, EN1, HOXA5 0.014 0.019 0.02 0.022 0.023 0.027 0.032 0.034 0.034 0.035
OTX2, EN2, GBX2 0.026 0.028 0.029 0.033 0.035 0.037 0.037 0.037 0.039 0.039
OTX2, EN2, IRX3 0.015 0.018 0.018 0.019 0.02 0.021 0.021 0.024 0.026 0.026
OTX2, EN2, HOXA1 0.03 0.03 0.031 0.032 0.032 0.033 0.034 0.035 0.035 0.036
OTX2, EN2, HOXA2 0.012 0.014 0.015 0.015 0.018 0.02 0.022 0.026 0.026 0.027
OTX2, EN2, HOXA3 0.011 0.011 0.012 0.012 0.013 0.014 0.014 0.014 0.019 0.021
OTX2, EN2, HOXA4 0.01 0.016 0.017 0.017 0.019 0.019 0.02 0.021 0.026 0.027
OTX2, EN2, HOXA5 0.012 0.016 0.018 0.019 0.02 0.022 0.027 0.028 0.03 0.031
OTX2, WNT1, GBX2 0.019 0.019 0.021 0.028 0.034 0.036 0.036 0.037 0.039 0.041
OTX2, WNT1, IRX3 0.016 0.016 0.017 0.017 0.019 0.023 0.024 0.026 0.026 0.027
OTX2, WNT1, HOXA1 0.025 0.027 0.028 0.03 0.031 0.032 0.033 0.037 0.037 0.041
OTX2, WNT1, HOXA2 0.0088 0.0089 0.0093 0.0096 0.0098 0.01 0.012 0.012 0.015 0.017
OTX2, WNT1, HOXA3 0.0093 0.0099 0.01 0.011 0.013 0.013 0.014 0.016 0.027 0.027
OTX2, WNT1, HOXA4 0.0091 0.0092 0.0094 0.0096 0.01 0.011 0.011 0.018 0.019 0.019
OTX2, WNT1, HOXA5 0.014 0.017 0.018 0.019 0.025 0.025 0.026 0.027 0.028 0.03
OTX2, LMX1A, GBX2 0.022 0.024 0.025 0.026 0.027 0.027 0.031 0.033 0.033 0.036
OTX2, LMX1A, IRX3 0.021 0.021 0.022 0.023 0.024 0.025 0.027 0.027 0.034 0.039
OTX2, LMX1A, HOXA1 0.023 0.024 0.027 0.029 0.03 0.031 0.037 0.04 0.042 0.042
OTX2, LMX1A, HOXA2 0.011 0.014 0.014 0.014 0.014 0.015 0.016 0.016 0.017 0.019
OTX2, LMX1A, HOXA3 0.013 0.013 0.013 0.014 0.014 0.016 0.016 0.017 0.018 0.021
OTX2, LMX1A, HOXA4 0.01 0.012 0.012 0.013 0.014 0.014 0.016 0.017 0.017 0.021
OTX2, LMX1A, HOXA5 0.017 0.019 0.023 0.023 0.025 0.031 0.031 0.034 0.035 0.038
OTX2, LMX1B, GBX2 0.02 0.026 0.029 0.033 0.034 0.035 0.04 0.044 0.044 0.046
OTX2, LMX1B, IRX3 0.017 0.017 0.018 0.018 0.018 0.019 0.02 0.023 0.023 0.023
OTX2, LMX1B, HOXA1 0.022 0.023 0.028 0.03 0.03 0.032 0.034 0.034 0.036 0.037
OTX2, LMX1B, HOXA2 0.011 0.011 0.011 0.012 0.013 0.013 0.013 0.014 0.015 0.015
OTX2, LMX1B, HOXA3 0.011 0.013 0.014 0.016 0.016 0.017 0.018 0.018 0.02 0.02
OTX2, LMX1B, HOXA4 0.011 0.012 0.014 0.016 0.017 0.018 0.019 0.019 0.02 0.02
OTX2, LMX1B, HOXA5 0.015 0.017 0.017 0.017 0.017 0.017 0.019 0.019 0.023 0.024
OTX2, PAX2, GBX2 0.025 0.025 0.028 0.029 0.032 0.033 0.033 0.034 0.039 0.039
OTX2, PAX2, IRX3 0.021 0.022 0.022 0.023 0.023 0.024 0.026 0.027 0.027 0.028
OTX2, PAX2, HOXA1 0.027 0.027 0.029 0.033 0.036 0.036 0.036 0.037 0.037 0.044
OTX2, PAX2, HOXA2 0.015 0.015 0.016 0.017 0.018 0.019 0.02 0.02 0.021 0.023
OTX2, PAX2, HOXA3 0.015 0.017 0.019 0.02 0.02 0.021 0.022 0.023 0.028 0.029
OTX2, PAX2, HOXA4 0.016 0.018 0.019 0.019 0.024 0.026 0.028 0.033 0.035 0.039
OTX2, PAX2, HOXA5 0.018 0.019 0.021 0.025 0.025 0.026 0.027 0.027 0.028 0.03
OTX2, PAX5, GBX2 0.022 0.023 0.03 0.03 0.03 0.034 0.034 0.034 0.035 0.035
OTX2, PAX5, IRX3 0.016 0.018 0.018 0.019 0.02 0.021 0.024 0.026 0.027 0.027
OTX2, PAX5, HOXA1 0.025 0.027 0.028 0.029 0.029 0.032 0.034 0.04 0.04 0.041
OTX2, PAX5, HOXA2 0.013 0.013 0.017 0.017 0.017 0.018 0.018 0.019 0.019 0.019
OTX2, PAX5, HOXA3 0.013 0.014 0.014 0.014 0.016 0.017 0.02 0.02 0.02 0.022
OTX2, PAX5, HOXA4 0.013 0.015 0.017 0.019 0.019 0.02 0.022 0.023 0.023 0.024
OTX2, PAX5, HOXA5 0.017 0.018 0.021 0.026 0.026 0.027 0.029 0.03 0.033 0.034
OTX2, PAX7, GBX2 0.022 0.022 0.024 0.025 0.025 0.027 0.029 0.031 0.032 0.035
OTX2, PAX7, IRX3 0.015 0.017 0.017 0.017 0.018 0.019 0.02 0.023 0.025 0.032
OTX2, PAX7, HOXA1 0.023 0.027 0.027 0.028 0.028 0.028 0.029 0.029 0.031 0.036
OTX2, PAX7, HOXA2 0.0096 0.011 0.012 0.012 0.012 0.013 0.014 0.015 0.016 0.016
OTX2, PAX7, HOXA3 0.01 0.012 0.012 0.012 0.012 0.014 0.015 0.017 0.018 0.018
OTX2, PAX7, HOXA4 0.011 0.011 0.012 0.012 0.014 0.016 0.017 0.017 0.017 0.017
OTX2, PAX7, HOXA5 0.015 0.016 0.017 0.018 0.018 0.019 0.019 0.02 0.02 0.024
OTX2, PAX8, GBX2 0.032 0.033 0.033 0.033 0.033 0.035 0.037 0.037 0.038 0.04
OTX2, PAX8, IRX3 0.016 0.016 0.018 0.02 0.02 0.023 0.023 0.024 0.024 0.024
OTX2, PAX8, HOXA1 0.025 0.025 0.029 0.029 0.029 0.031 0.033 0.037 0.038 0.038
OTX2, PAX8, HOXA2 0.0098 0.01 0.011 0.012 0.013 0.015 0.016 0.017 0.018 0.019
OTX2, PAX8, HOXA3 0.013 0.014 0.015 0.015 0.017 0.017 0.018 0.018 0.019 0.02
OTX2, PAX8, HOXA4 0.01 0.011 0.014 0.015 0.015 0.015 0.016 0.016 0.016 0.018
OTX2, PAX8, HOXA5 0.011 0.017 0.022 0.022 0.023 0.023 0.027 0.028 0.031 0.034
OTX2, FGF8, GBX2 0.027 0.029 0.033 0.035 0.035 0.036 0.038 0.039 0.04 0.041
OTX2, FGF8, IRX3 0.016 0.019 0.022 0.023 0.024 0.034 0.035 0.042 0.042 0.044
OTX2, FGF8, HOXA1 0.022 0.027 0.029 0.03 0.03 0.03 0.034 0.04 0.041 0.045
OTX2, FGF8, HOXA2 0.015 0.017 0.018 0.018 0.02 0.021 0.021 0.025 0.026 0.028
OTX2, FGF8, HOXA3 0.015 0.017 0.018 0.018 0.018 0.019 0.02 0.02 0.021 0.021
OTX2, FGF8, HOXA4 0.014 0.015 0.016 0.017 0.019 0.02 0.02 0.02 0.021 0.023
OTX2, FGF8, HOXA5 0.015 0.018 0.02 0.023 0.023 0.024 0.025 0.025 0.026 0.031
OTX2, FGF17, GBX2 0.025 0.027 0.031 0.033 0.035 0.037 0.041 0.041 0.042 0.046
OTX2, FGF17, IRX3 0.017 0.018 0.02 0.02 0.02 0.022 0.022 0.022 0.025 0.03
OTX2, FGF17, HOXA1 0.023 0.026 0.027 0.03 0.034 0.042 0.042 0.043 0.043 0.046
OTX2, FGF17, HOXA2 0.011 0.011 0.012 0.012 0.014 0.015 0.015 0.016 0.017 0.017
OTX2, FGF17, HOXA3 0.012 0.012 0.012 0.012 0.013 0.013 0.014 0.015 0.016 0.019
OTX2, FGF17, HOXA4 0.012 0.013 0.014 0.016 0.016 0.017 0.017 0.023 0.024 0.024
OTX2, FGF17, HOXA5 0.012 0.019 0.02 0.021 0.023 0.026 0.03 0.03 0.032 0.032

Figure S3, Cost function values of the 10 best tri-stable GRN optimization runs, for all 420 tested (FB, MB, HB) gene combinations.
It is clear that the tri-stable GRN can be made to agree better with the data of some gene combinations than others. For example, cost function values are generally lower for FB = FOXG1 or LHX2 than for FB = TBR1. The cost function value of the parameter set used for the simulations in Figure 3, where (FB, MB, HB) = (FOXG1, WNT1, HOXA3), is framed in green.

A

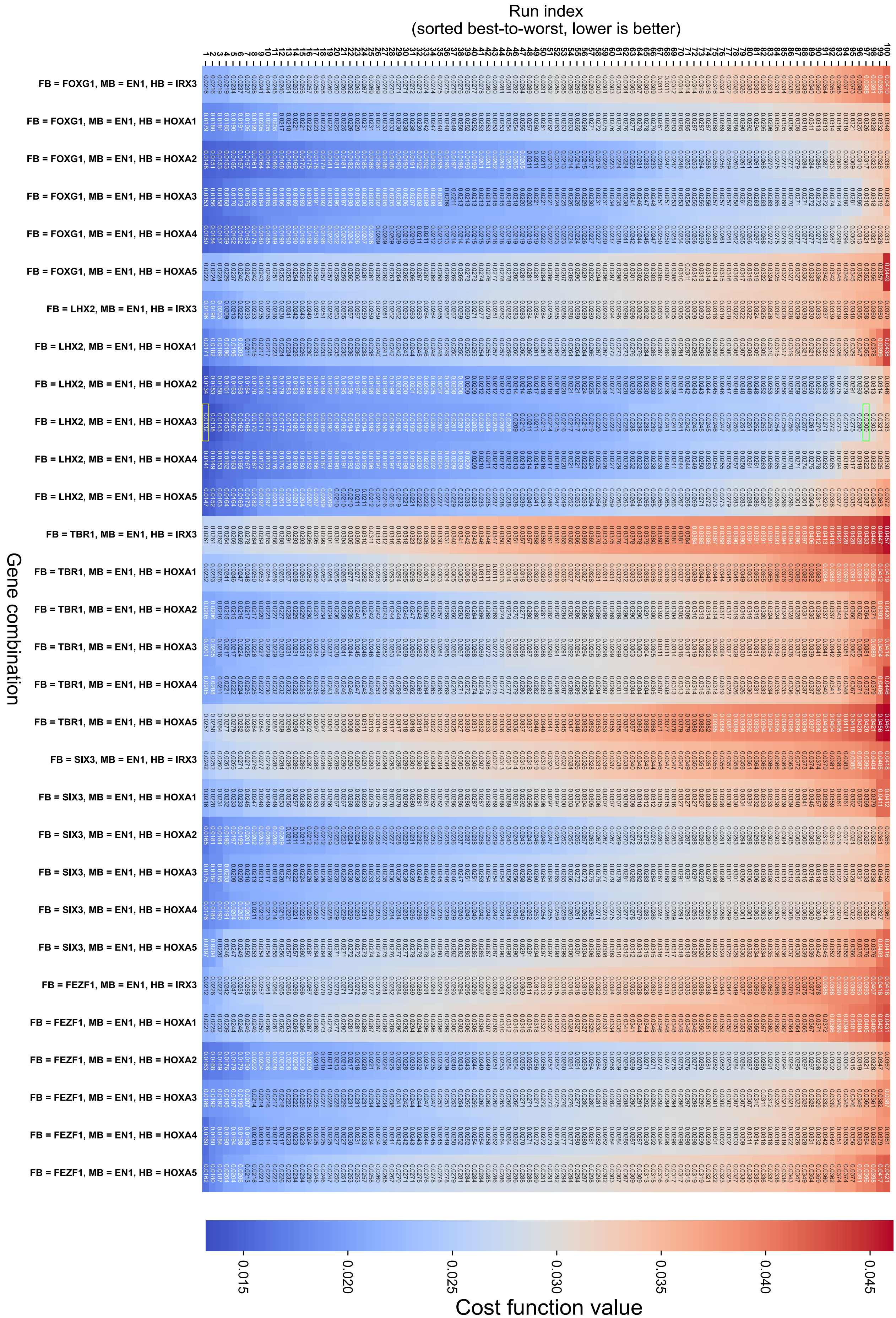
Run index
(sorted best-to-worst, lower is better)
Gene combination
FB = FOXG1, MB = EN1, HB = IRX3
FB = FOXG1, MB = EN1, HB = HOXA1
FB = FOXG1, MB = EN1, HB = HOXA2
FB = FOXG1, MB = EN1, HB = HOXA3
FB = FOXG1, MB = EN1, HB = HOXA4
FB = FOXG1, MB = EN1, HB = HOXA5
FB = LHX2, MB = EN1, HB = IRX3
FB = LHX2, MB = EN1, HB = HOXA1
FB = LHX2, MB = EN1, HB = HOXA2
FB = LHX2, MB = EN1, HB = HOXA3
FB = LHX2, MB = EN1, HB = HOXA4
FB = LHX2, MB = EN1, HB = HOXA5
FB = TBR1, MB = EN1, HB = IRX3
FB = TBR1, MB = EN1, HB = HOXA1
FB = TBR1, MB = EN1, HB = HOXA2
FB = TBR1, MB = EN1, HB = HOXA3
FB = TBR1, MB = EN1, HB = HOXA4
FB = TBR1, MB = EN1, HB = HOXA5
FB = SIX3, MB = EN1, HB = IRX3
FB = SIX3, MB = EN1, HB = HOXA1
FB = SIX3, MB = EN1, HB = HOXA2
FB = SIX3, MB = EN1, HB = HOXA3
FB = SIX3, MB = EN1, HB = HOXA4
FB = SIX3, MB = EN1, HB = HOXA5
FB = FEZF1, MB = EN1, HB = IRX3
FB = FEZF1, MB = EN1, HB = HOXA1
FB = FEZF1, MB = EN1, HB = HOXA2
FB = FEZF1, MB = EN1, HB = HOXA3
FB = FEZF1, MB = EN1, HB = HOXA4
FB = FEZF1, MB = EN1, HB = HOXA5
0.015
0.020
0.025
0.030
0.035
0.040
0.045
Cost function value

B

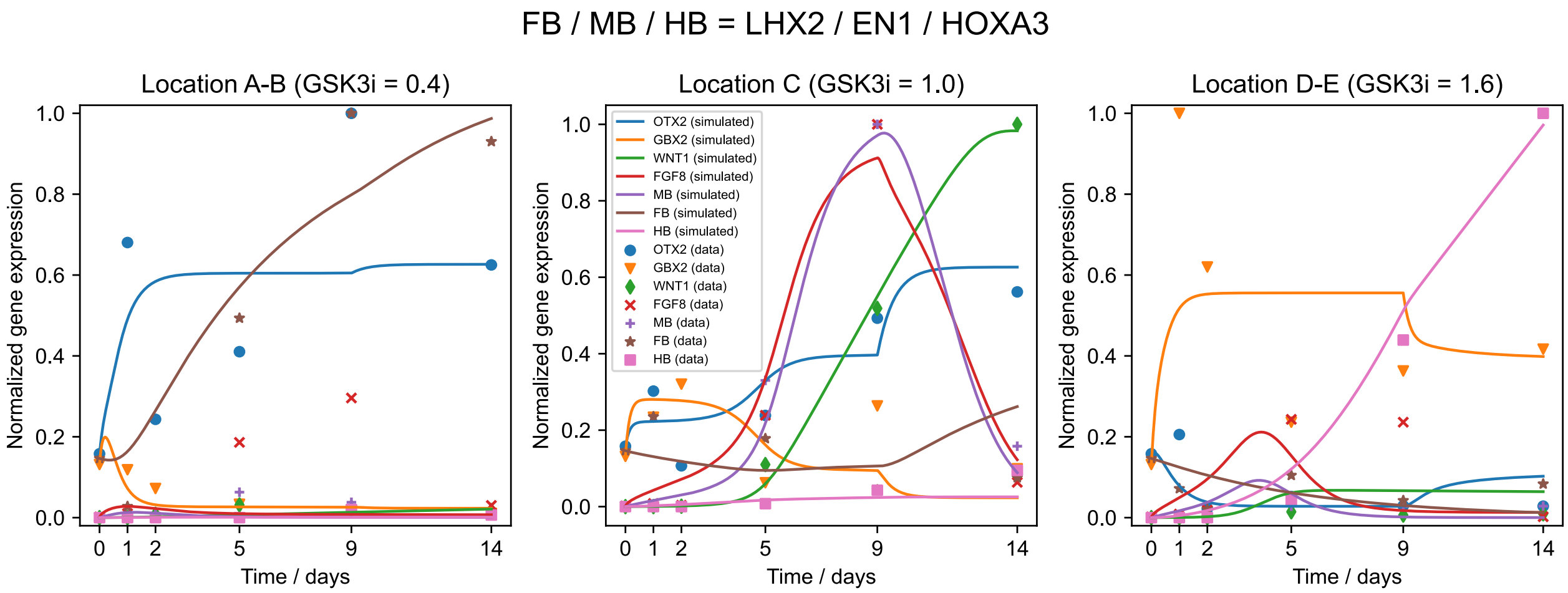

Figure S4, All cost function values and the best fit with data for the combined tri-stable/isthmic organizer GRN.
(**A**) Cost function values of all 100 optimization runs for all (FB, HB) gene combinations where MB = EN1. The cost function value of the parameter set used for the simulations in Figure 4 and Figure 6, where (FB, MB, HB) = (LHX2, EN1, HOXA3), is framed in green. The best agreement with the data was also for this particular gene combination; the cost function value of that parameter set, used for the simulations shown in **B** below, is framed in yellow. (**B**) Simulation of combined tri-stable/isthmic organizer GRN dynamics using the optimal parameter set (lowest cost function value) for the gene combination (FB, MB, HB) = (LHX2, EN1, HOXA3) in **A**. The simulated dynamics are plotted alongside scRNA-seq data for the three GSK3i levels observed during days 0–9, against which the model was optimized. Compared to the results shown in Figure 4, simulations with this parameter set demonstrate improved agreement with experimental data. Specifically, the simulated levels of EN1 and FGF8 decline to low values by day 14 in location C, where only WNT1 remains elevated following the removal of GSK3i at day 9.

Dorsal-ventral topology with both self-activations and Gli repressions

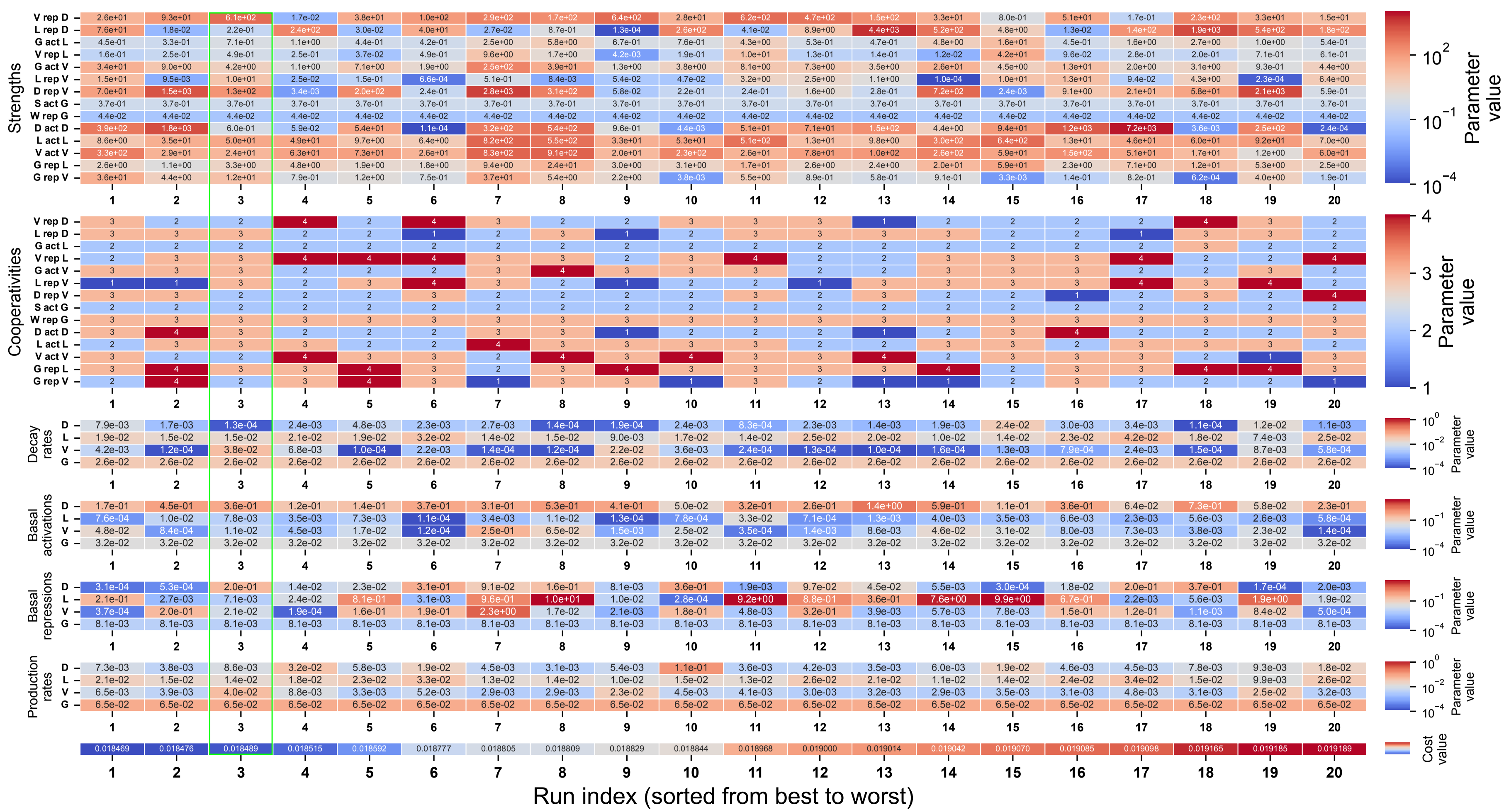

Figure S5, Parameters and cost function values of the 20 best optimization runs for the dorsal-ventral GRN topology with both self-activations and GLI repressions.
Parameters are labeled according to their associated gene regulatory network (GRN) node: D (dorsal), L (lateral), V (ventral), G (GLI), W (WNT), and S (SHH). Interaction strength and cooperativity parameters are denoted as "X act Y" or "X rep Y," indicating whether they correspond to the activation or repression of node Y by node X, respectively. The parameter set used for the simulations in Figure 5 and Figure 6 is framed in green.

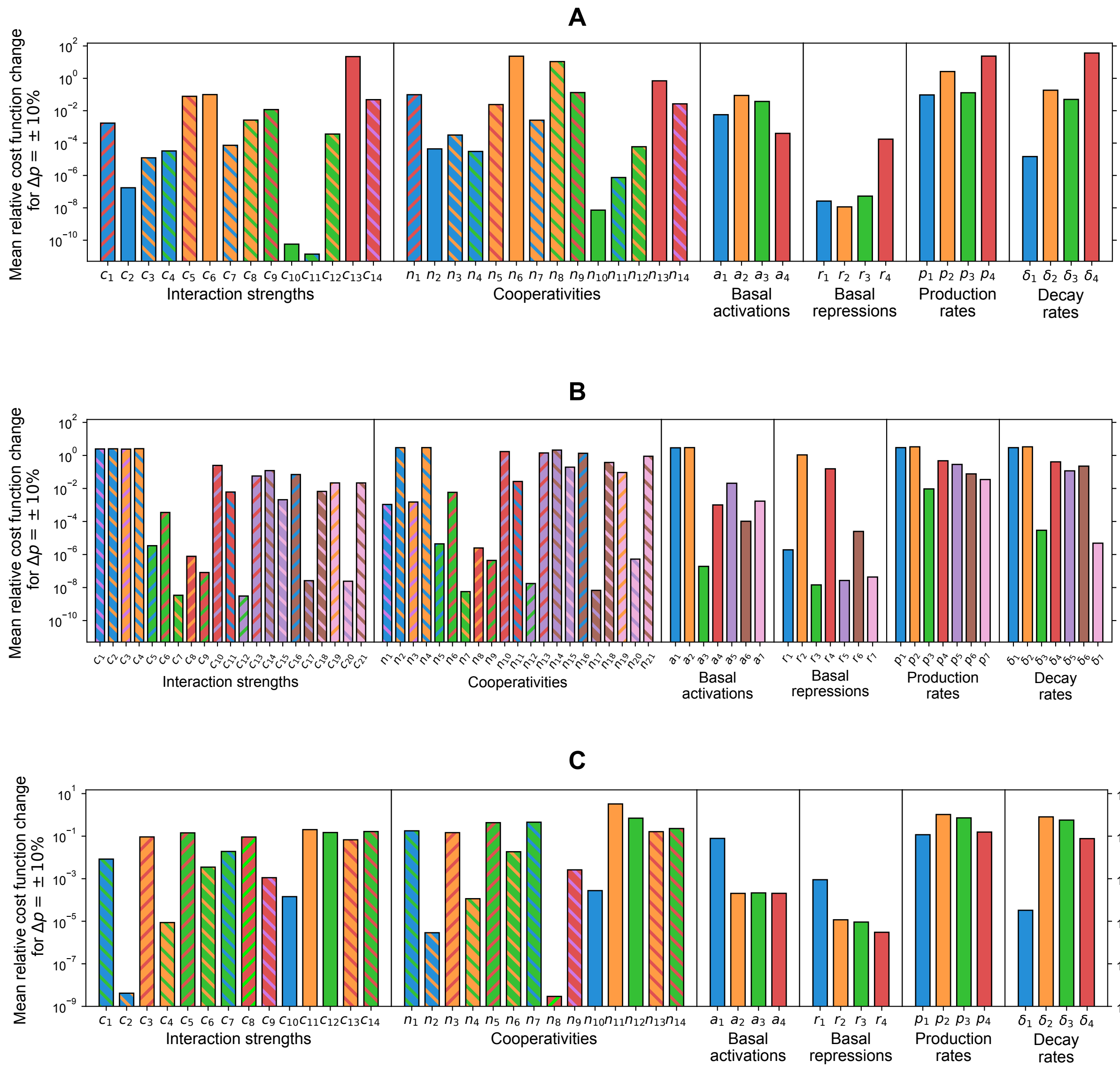

Figure S6, Sensitivity analyses for winning GRN model parameter sets.
Sensitivity was assessed by evaluating the relative changes in the cost function resulting from varying each parameter individually by ±10% (see METHODS). The bars in the plot are color-coded according to the gene regulatory network (GRN) node associated with each parameter. Dashed bars indicate parameters linked to a GRN node that is regulated by another node, with the color of the dashes corresponding to the regulating GRN node. Diagonally upward dashes denote activation, while diagonally downward dashes denote repression. (**A**) Sensitivity analysis of the tri-stable GRN. Variations in the production and degradation rates of GSK3 significantly influence the simulated behavior of the model. Similarly, alterations in the cooperativity of midbrain (MB) self-activation, the repression of MB by hindbrain (HB), and the strength of GSK3 self-activation also exert substantial effects on model dynamics. (**B**) Sensitivity analysis of the combined tri-stable and isthmic organizer GRN. Alterations in parameters associated with OTX2 and GBX2, as well as their regulation by GSK3i, substantially influence the simulated behavior of the model. This sensitivity can be attributed to the critical role of the OTX2-GBX2 regulatory switch in both positioning and maintaining the isthmic organizer. Incorrect function of this switch disrupts the spatial organization of downstream gene expression, leading to pronounced effects on the overall model dynamics. (**C**) Sensitivity analysis of the dorsal-ventral GRN. The simulated behavior of the dorsal-ventral GRN topology, which incorporates both self-activations and repressive GLI interactions, is most significantly influenced by variations in production rates across all nodes. Additionally, changes in the degradation rates of the lateral, ventral, and GLI nodes, as well as alterations in the cooperativity of self-activation for the lateral and ventral nodes, exert substantial effects on model dynamics.
